# Expanding the scope of plant genome engineering with Cas12a orthologs and highly multiplexable editing systems

**DOI:** 10.1038/s41467-021-22330-w

**Published:** 2021-03-29

**Authors:** Yingxiao Zhang, Qiurong Ren, Xu Tang, Shishi Liu, Aimee A. Malzahn, Jianping Zhou, Jiaheng Wang, Desuo Yin, Changtian Pan, Mingzhu Yuan, Lan Huang, Han Yang, Yuxin Zhao, Qing Fang, Xuelian Zheng, Li Tian, Yanhao Cheng, Ysa Le, Bailey McCoy, Lidiya Franklin, Jeremy D. Selengut, Stephen M. Mount, Qiudeng Que, Yong Zhang, Yiping Qi

**Affiliations:** 1 Department of Plant Science and Landscape Architecture, University of Maryland, College Park, MD USA; 2 Department of Biotechnology, School of Life Science and Technology, Center for Informational Biology, University of Electronic Science and Technology of China, Chengdu, China; 3 Food Crop Institute, Hubei Academy of Agricultural Sciences, Wuhan, Hubei China; 4 College of Agriculture, Nanjing Agricultural University, Nanjing, Jiangsu China; 5 Center for Bioinformatics and Computational Biology, University of Maryland, College Park, MD USA; 6 Department of Cell Biology and Molecular Genetics, University of Maryland, College Park, MD USA; 7 Syngenta, Research Triangle Park, NC USA; 8 Institute for Bioscience and Biotechnology Research, University of Maryland, Rockville, MD USA

**Keywords:** Genetic engineering, Genomics, CRISPR-Cas9 genome editing

## Abstract

CRISPR-Cas12a is a promising genome editing system for targeting AT-rich genomic regions. Comprehensive genome engineering requires simultaneous targeting of multiple genes at defined locations. Here, to expand the targeting scope of Cas12a, we screen nine Cas12a orthologs that have not been demonstrated in plants, and identify six, ErCas12a, Lb5Cas12a, BsCas12a, Mb2Cas12a, TsCas12a and MbCas12a, that possess high editing activity in rice. Among them, Mb2Cas12a stands out with high editing efficiency and tolerance to low temperature. An engineered Mb2Cas12a-RVRR variant enables editing with more relaxed PAM requirements in rice, yielding two times higher genome coverage than the wild type SpCas9. To enable large-scale genome engineering, we compare 12 multiplexed Cas12a systems and identify a potent system that exhibits nearly 100% biallelic editing efficiency with the ability to target as many as 16 sites in rice. This is the highest level of multiplex edits in plants to date using Cas12a. Two compact single transcript unit CRISPR-Cas12a interference systems are also developed for multi-gene repression in rice and *Arabidopsis*. This study greatly expands the targeting scope of Cas12a for crop genome engineering.

## Abstract (web summary)

CRISPR-Cas12a is a promising system for targeting AT-rich regions of the genome. Here the authors identify Cas12a orthologs with expanded targeting scope and develop a highly multiplexable editing system in rice.

## Introduction

CRISPR-Cas12a (formerly Cpf1) is a class II type V endonuclease that prefers a thymine-rich protospacer adjacent motif (PAM)^1^, and is the second most commonly used CRISPR system for genome editing^2–4^. Cas12a has shown higher targeting specificity than Cas9 in mammalian cells and plants^5–7^. Unlike Cas9, Cas12a generates staggered ends and larger deletions, making it a suitable nuclease for gene knockout. Moreover, Cas12a only requires a short CRISPR RNA (crRNA) for each target and possesses RNase activity for crRNA array processing, making it an excellent platform for multiplexed editing^8,9^. Cas12a has been used to generate targeted mutations as well as achieve transcriptional regulation in microorganisms^10–12^. Cas12a can also efficiently generate edits in some important industrial *Streptomyces* strains that cannot be edited using SpCas9 due to toxicity^12^. In mammalian systems, Cas12a has been widely used and engineered for genetic manipulation^1,13–15^. Three Cas12a orthologs, LbCas12a, AsCas12a, and FnCas12a, have been used in plants. LbCas12a is most popular due to its high editing activity in rice^9,16–22^, maize^23,24^, *Arabidopsis*^24–26^, tomato^25^, *Nicotiana benthamiana*^25^, lettuce^27^, cotton^28^, and citrus^29^. FnCas12a also mediates efficient genome editing in plants^9,17,20,30^. While most Cas12a studies in plants pursued targeted mutagenesis by non-homologous end joining (NHEJ) DNA repair, precise genome editing based on homology-directed repair (HDR) has also been demonstrated^20,22,31–33^. Furthermore, Cas12a has been repurposed for base editing^15,34^ and transcriptional regulation^15,16,35–37^.

Plant genome editing is an exciting technology to accelerate crop breeding. The versatility and simplicity of the CRISPR-Cas12a system allow simultaneous modification of many genes in elite cultivars, that may be difficult or impractical to achieve with conventional breeding and genetic engineering methods. However, the TTTV (V = A, C, and G) PAM requirement of Cas12a limits its targeting scope. Many attempts have been made to engineer Cas12a nucleases^13–15,38^ or explore novel Cas12a^1,14,39–41^ with relaxed PAMs in vitro and in mammalian cells. In plants, although FnCas12a has been demonstrated to target some VTTV PAMs^17,30^ and engineered LbCas12a can target altered PAMs^17,42^, further improvement is required to edit more relaxed PAMs. The second limitation is that current Cas12a orthologs in use are not effective at lower temperatures^24,43^, though this could partially be addressed by protein engineering^15,26^. Moreover, a multiplexing system with high efficiency and capacity is required to enable versatile genome engineering in plants. Although many multiplexing strategies have been developed in plants using Cas9 and Cas12a (Supplementary Tables 1 and 2), a system that can achieve highly efficient biallelic editing at multiple targets has not been demonstrated. With low biallelic editing efficiency, it is laborious or impractical to obtain multigene knockouts in crops through subsequent breeding. To address these issues, we comprehensively assess nine Cas12a orthologs and engineered their variants in rice for efficient genome editing with expanded targeting ranges. We also compared 12 multiplexed Cas12a systems to identify the most potent one for large-scale plant genome engineering. Collectively, we establish a Cas12a toolbox for efficient multiplexed genome engineering with improved target accessibility and scalability.

## Results

### Genome editing using nine Cas12a orthologs in rice cells

**Fig. 1:**
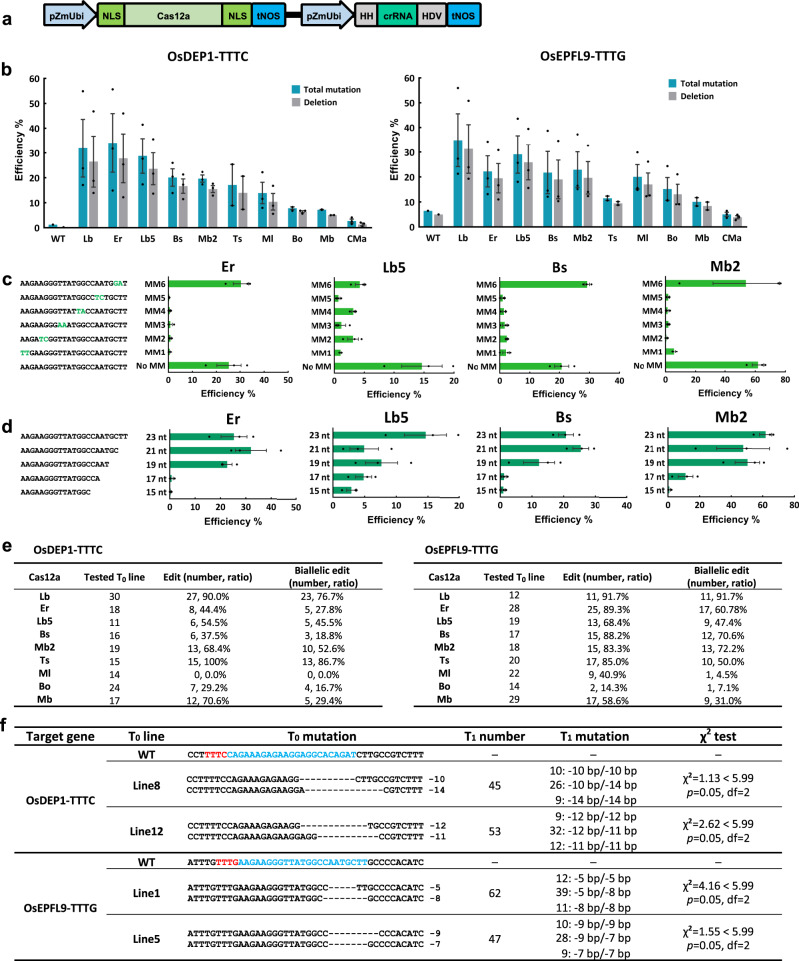
Targeted mutagenesis by Cas12a orthologs at canonical TTTV PAM sites in rice. **a** A schematic diagram of the Cas12a expression system for single site targeting. pZmUbi maize ubiquitin promoter, NLS nuclear localization signal, tNOS nopaline synthase (NOS) terminator, HH hammerhead ribozyme, HDV hepatitis delta virus ribozyme. **b** Targeted mutagenesis efficiencies (percentage) of ten Cas12a orthologs at two TTTV PAM sites in rice protoplasts. WT, protoplasts transformed with water. **c** Targeting specificity of four Cas12a orthologs measured with mismatched crRNAs at the OsEPFL9-TTTG site in rice protoplasts. Mismatched nucleotides in crRNAs are highlighted in green. **d** Protospacer length requirements of four Cas12a orthologs at the OsEPFL9-TTTG site in rice protoplasts. **e** Summary of editing and biallelic editing efficiencies of Cas12a orthologs at two TTTV PAM sites in transgenic rice *T*_0_ lines. **f** Editing analysis of transgenic rice *T*_1_ lines. *χ*^2^ test results indicate that *T*_1_ populations from each tested *T*_0_ line segregated at a 1:2:1 ratio when *α* = 0.05. Data in “**b**–**d**” are presented as mean values ± SEM. *n* = 3 biologically independent samples. Source data are provided as a Source Data file.

To improve Cas12a genome editing in plants, we chose nine Cas12a orthologs that have not been demonstrated in plants (Supplementary Fig. 1). We included eight Cas12a orthologs that showed a preference for TTV PAMs in vitro, including Lb5Cas12a, BsCas12a, Mb2Cas12a, TsCas12a, MlCas12a, BoCas12a, MbCas12a, and CMaCas12a^1,39^. In addition, we included ErCas12a^44^, which is also known as MAD7 (Inscripta, Inc.). The editing activity of these Cas12a orthologs, along with the control LbCas12a, was evaluated in rice protoplasts at two target sites, OsDEP1-TTTC and OsEPFL9-TTTG, using a dual RNA Polymerase II (Pol II) promoter expression system^16^ (Fig. 1a). Editing efficiencies were assessed by high-throughput amplicon sequencing using the Illumina HiSeq platform. All Cas12a orthologs, except CMaCas12a, resulted in targeted mutation frequencies of roughly 10% or greater at both sites (Fig. 1b and Supplementary Data 1). Four Cas12a orthologs (Er, Lb5, Bs, and Mb2) showed comparable editing frequencies to LbCas12a. Targeted mutations were predominately deletions distal from PAMs, centered around the predicted cleavage site^1^ (Fig. 1b and Supplementary Figs. 2 and 3). When mismatched protospacers were used, all four Cas12a orthologs could only tolerate mismatches at the last three base pairs distal from the PAM, indicating high targeting specificity (Fig. 1c, Supplementary Fig. 4, and Supplementary Data 1). Protospacers of 19–23 nt could generate high editing frequencies for ErCas12a and Mb2Cas12a, while longer protospacers were required for Lb5Cas12a and BsCas12a to achieve robust activity (Fig. 1d, Supplementary Fig. 4, and Supplementary Data 1).

### Genome editing using eight Cas12a orthologs in stable transgenic rice

We next assessed these eight Cas12a orthologs (except CMaCas12a) in stable transgenic rice lines at the same two sites. All eight Cas12a orthologs could generate biallelic mutants in rice (Fig. 1e and Supplementary Figs. 5, 6) and most of them (e.g., Er, Lb5, Bs, Mb2, Ts, and Mb) showed medium to high editing frequencies (Fig. 1e). At the OsDEP1-TTTC site, TsCas12a showed 100% editing frequency, which was even higher than LbCas12a; three other Cas12a orthologs (Lb5, Mb2, and Mb) also had high editing frequencies from 54.5 to 70.6% (Fig. 1e). At the OsEPFL9-TTTG site, four Cas12a orthologs (Er, Bs, Mb2, and Ts) showed comparable editing frequencies (83.3–89.5%) to LbCas12a (91.7%) (Fig. 1e). Together, these data suggest the tested Cas12a orthologs can induce medium to high-frequency genome editing in rice except for MlCas12a and BoCas12a. To assess the inheritance of targeted mutations, we examined Mb2Cas12a *T*_1_ populations derived from two *T*_0_ lines for each target site. In all cases, mutations were stably inherited with the expected 1:2:1 Mendelian segregation ratio (Fig. 1f).

### Mb2Cas12a can efficiently target VTTV PAMs

**Fig. 2:**
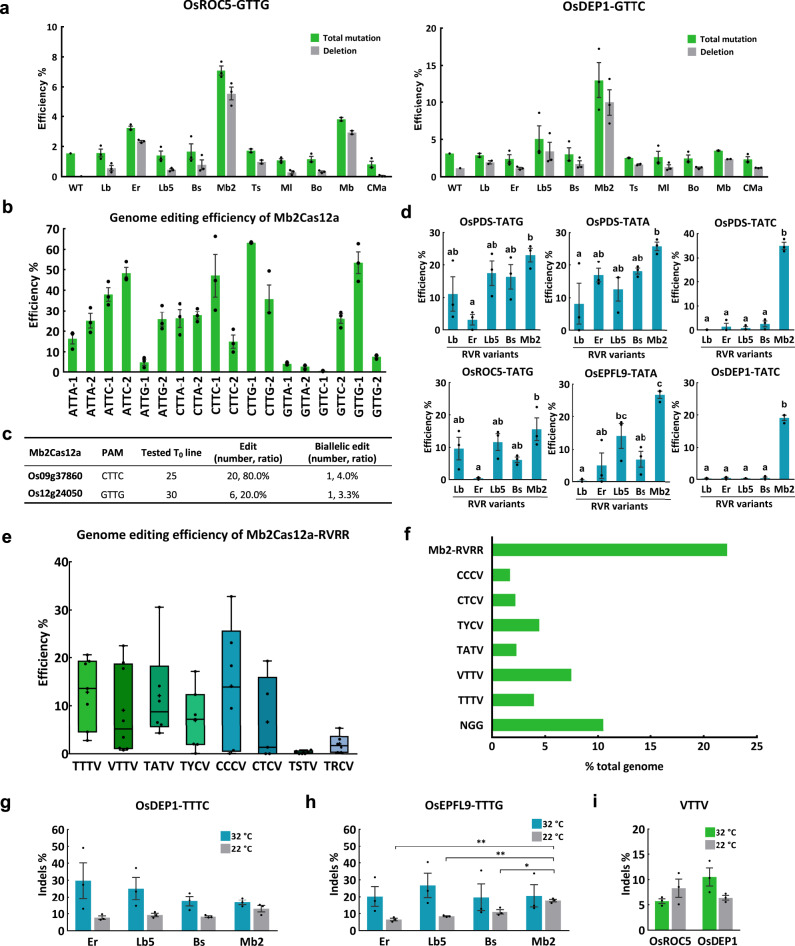
Mb2Cas12a and engineered Cas12a variants broaden the genome editing scope in rice. **a** Targeted mutagenesis efficiencies (percentage) of ten Cas12a orthologs at two VTTV PAM sites in rice protoplasts. WT, protoplasts transformed with water. **b** Targeted mutagenesis efficiencies (percentage) of Mb2Cas12a at 18 VTTV PAM sites in rice protoplasts. **c** Summary of editing and biallelic editing efficiencies by Mb2Cas12a at two VTTV PAM sites in transgenic rice *T*_0_ lines. **d** Comparison of editing efficiencies (percentage) of five Cas12a RVR variants at six TATV PAM sites in rice protoplasts. Treatments with the same letter are not significantly different when *α* = 0.05 by Tukey’s Honest Significant Difference test (two-sided). **e** A box plot showing targeted mutagenesis efficiencies (percentage) of Mb2Cas12a-RVRR variant at 51 canonical and altered PAM sites in rice protoplasts. V = A, C, and G. Y = T and C. S = C and G. R = A and G. Each data point represents the mean of three biological replicates (*n* = 3). The middle line in each box denotes the median. The upper and lower bounds of each box denote the first quartile (25%) and the third quartile (75%), respectively. The top and bottom of whiskers denote the maxima and minima, respectively. **f** Genome-wide analysis of targetable PAM sites by Mb2Cas12a-RVRR variant in rice (*Oryza sativa*). **g** and **h**, Insertion and deletion (Indel) frequencies (percentage) of four Cas12a orthologs at two TTTV PAM sites at 32 and 22 °C in rice protoplasts. One asterisk (*p* < 0.05) and two asterisks (*p* < 0.01) indicate significant differences between two treatments using two-sided Student’s *t*-test. *p* = 0.000583 (Er vs. Mb2); *p* = 0.000456 (Lb5 vs. Mb2); *p* = 0.012092 (Bs vs. Mb2). **i** Indel frequencies (percentage) of Mb2Cas12a at two VTTV PAM sites at 32 and 22 °C in rice protoplasts. Data in **a**, **b**, **d**, **g**–**i** are presented as mean values ± SEM. *n* = 3 biologically independent samples. Source data are provided as a Source Data file.

To assess whether the tested Cas12a orthologs can target shorter PAMs, we measured their editing activity at two relaxed VTTV PAM sites, OsROC5-GTTG and OsDEP1-GTTC. LbCas12a and most of the Cas12a orthologs showed low efficient or no editing (Fig. 2a and Supplementary Data 1). However, Mb2Cas12a displayed editing efficiencies of ~10% at both target sites (Fig. 2a and Supplementary Data 1) and resulted in largely 3–12 bp deletions (Supplementary Fig. 7). We next tested Mb2Cas12a at 18 target sites with two sites for each possible VTTV combination (Supplementary Fig. 8). Mb2Cas12a could efficiently edit 13 out of 18 target sites with mutation frequencies of approximately 15% or greater (Fig. 2b and Supplementary Data 1). Among these 13 sites, ATTA-1 and ATTC-2, which could not be edited by FnCas12a^17^, were edited by Mb2Cas12a with efficiencies of 16.3% and 48.3%, respectively. Moreover, Mb2Cas12a could edit GTTA and GTTC PAM sites, which were not editable by FnCas12a^17^. Further, we found Mb2Cas12a could reliably generate stable knockouts of two target genes at VTTV PAM sites (Fig. 2c and Supplementary Fig. 9). Taken together, Mb2Cas12a can reliably target almost all NTTV PAMs in rice.

### The RVR variant of Mb2Cas12a can efficiently target TATV PAMs

Previously, RVR variants of AsCas12a and LbCas12a were engineered to target TATV PAMs in mammalian cells^13^. We showed that LbCas12a-RVR could only target TATG PAMs while FnCas12a-RVR had no activity at TATV PAMs in rice^17^. To seek Cas12a variants that can truly target all TATV PAMs, we engineered RVR variants of ErCas12a, Lb5Cas12a, BsCas12a, and Mb2Cas12a (Supplementary Fig. 1b), and tested them in rice protoplasts (Supplementary Fig. 10 and Supplementary Data 1). LbCas12b-RVR^17^ was included as a control. RVR variants of Lb5Cas12a, BsCas12a, and Mb2Cas12a showed robust editing at the two TATG PAM sites, as with LbCas12a-RVR (Fig. 2d). All four RVR variants could edit both TATA PAM sites with higher average editing efficiencies than LbCas12a-RVR (Fig. 2d). However, only Mb2Cas12a-RVR could edit the two TATC PAM sites (Fig. 2d). Impressively, Mb2Cas12a-RVR displayed high editing frequencies at all TATV PAM sites, ranging from 15 to 35%, which outperformed all other tested RVR variants.

### The RVRR variant of Mb2Cas12a has a broad target range

To further broaden the target range of Mb2Cas12a, we engineered the RVRR variant by introducing the K625R mutation into its RVR variant (Supplementary Fig. 11). We tested its editing activity at 51 targeting sites with canonical and altered PAMs in rice protoplasts (Supplementary Data 1). Mb2Cas12a-RVRR showed editing activities at TTTV, VTTV, TATV, TYCV, CCCV, and CTCV PAM sites (Y = T and C) (Fig. 2e). The variant however showed low or no editing activity at TSTV and TRCV PAM sites (S = C and G, R = A and G) (Fig. 2e). The RVRR variant of Mb2Cas12a significantly broadens the target range by Cas12a.

### Mb2Cas12a greatly expands the scope of crop genome editing

To assess the targeting scope of Mb2Cas12a and its variants, we conducted genome-wide PAM analysis in rice (*Oryza sativa*). The analysis showed the canonical Cas12a’s TTTV PAM sites only cover 4.0% of the genomes (Fig. 2f). Additional 7.5% and 2.3% genomic area become accessible with VTTV PAMs and TATV PAMs by using Mb2Cas12a and Mb2Cas12a-RVR, respectively (Fig. 2f). Remarkably, all the sites targetable by Mb2Cas12a-RVRR yield 22.2% coverage of the total genome, which is much higher than that of SpCas9 with an NGG PAM requirement (10.5%) (Fig. 2f).

### Mb2Cas12a is tolerant of low temperatures

Cas12a generally has reduced editing activity at lower temperatures^24,43^. We compared the editing activity of four Cas12a orthologs at 32 and 22 °C in rice protoplasts (Supplementary Data 1). At the two TTTV PAM sites, Mb2Cas12a activity was less affected by the low temperature and appeared to have higher Insertion and deletion (Indel) frequencies than ErCas12a, Lb5Cas12a, and BsCas12a (Fig. 2g, h). Editing activities were also detected at 22 °C with Mb2Cas12a at two VTTV PAM sites (Fig. 2i), further supporting Mb2Cas12a is tolerant of low temperature. Next, we tested two Mb2Cas12a variants (v1 and v2) harboring equivalent mutations of enhanced AsCas12a (enAsCas12a)^15^, and found Mb2Cas12a-v2 had improved activity at the OsEPFL9-TTTG PAM site at 22 °C (Supplementary Fig. 12). These data suggest Mb2Cas12a is less temperature sensitive and its activity can be further improved with protein engineering.

### Comparison of ten multiplex Cas12a genome editing systems

**Fig. 3:**
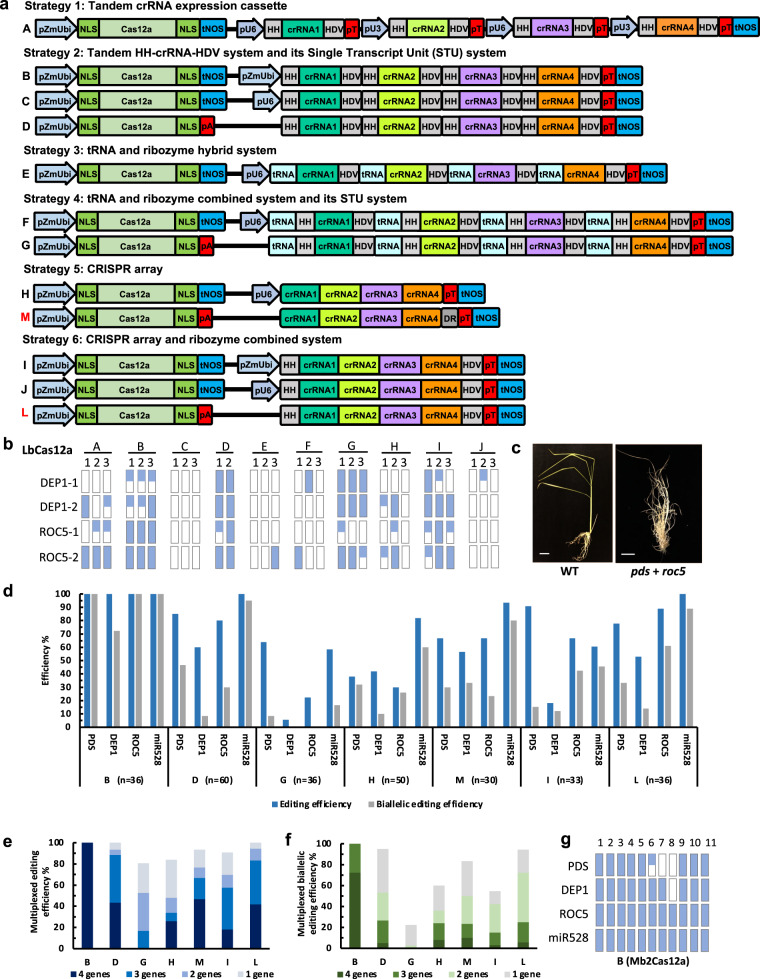
Comparison of 12 multiplex Cas12a genome editing systems in rice. **a** Schematics of 12 multiplexed Cas12a systems, which are classified into six different strategies. The schematics are based on multiplexing four crRNAs. pZmUbi maize ubiquitin promoter, NLS nuclear localization signal, tNOS nopaline synthase (NOS) terminator, pU6 rice U6 promoter, pU3 rice U3 promoter, HH hammerhead ribozyme, HDV hepatitis delta virus ribozyme, pT polyT, pA polyA, DR direct repeat. **b** Comparison of ten multiplexed Cas12a editing systems (A–J) in stable transgenic rice lines, by using LbCas12a to target *OsDEP1* and *OsROC5* with four crRNAs. Approximately three independent *T*_0_ lines were genotyped at each target site for wild type (denoted as an empty rectangle), monoallelic mutant (denoted as a half-filled rectangle) and biallelic mutant (denoted as a fully filled rectangle). **c** An example of a quadruple mutant showing loss-of-function phenotypes for *OsPDS* and *OsROC5*, compared to the wild type (WT). Additional quadruple mutants with similar phenotypes were identified. Size bar, 4 cm. **d** Comparison of seven multiplexed editing systems (B, D, G, H, M, I, and L) in stable transgenic rice lines, using LbCas12a to target four genes (*OsPDS*, *OsDEP1*, *OsROC5*, and *OsmiR528*) with four crRNAs. The number of transgenic *T*_0_ plants assayed are also indicated by “n”. **e** Further analysis of the data in “**d**” for editing efficiencies of each multiplexed LbCas12a system when editing certain defined numbers of genes (4, 3, 2, and 1) simultaneously. **f** Further analysis of the data in “**d**” for biallelic editing efficiencies of each multiplexed LbCas12a system when editing certain defined numbers of genes (4, 3, 2, and 1) simultaneously. **g** Multiplexed editing of four genes with system B by Mb2Cas12a. Genotypes at each target site in 11 *T*_0_ lines are shown with wild type denoted as an empty rectangle, monoallelic mutant denoted as a half-filled rectangle and biallelic mutant denoted as a fully filled rectangle. Source data are provided as a Source Data file.

To further expand the genome engineering scope with targeting multiple sites simultaneously, we sought to develop efficient multiplexed Cas12a systems. We first compared ten multiplexed systems (A through J) that can be grouped into six strategies (Fig. 3a). Strategy 1 utilizes tandem crRNA expression cassettes where each crRNA has its own promoter, either OsU6 or OsU3. The crRNAs are processed by the hammer head (HH) and hepatitis delta virus (HDV) ribozymes^16^. Strategy 2 compares the tandem HH-crRNA-HDV system under three expression conditions: by a Pol II promoter (pZmUbi), by a Pol III promoter (OsU6) or by a single transcript unit (STU) system. Strategy 3 uses a OsU6 to drive a tRNA-crRNA-HDV array, taking advantage of the tRNA’s promoter activity^45^. Given tRNA processing may leave a few extra nucleotides at the 5′ end^45^, Strategy 4 explores the use of a tRNA-HH-crRNA-HDV array to ensure more precise processing. Strategy 5 is the OsU6-driven CRISPR array system that has been previously explored^9^. Strategy 6 employs HH and HDV ribozymes to flank the entire crRNA array. Both pZmUbi and OsU6 promoters are compared in this strategy.

These ten systems were compared in stable transgenic rice lines using LbCas12a and four multiplexed crRNAs to simultaneously target two genes, *OsDEP1* and *OsROC5*. About three independent *T*_0_ lines were genotyped at each target site for each system. Based on the results, we could roughly rank these multiplex systems from high activity to low activity: System B and D > System G and I > System A > System H > System C, E, F, and J (Fig. 3b). The best performing multiplex systems (B and D) used a strong Pol II promoter to express an HH-crRNA-HDV array and resulted in high frequency biallelic mutations in *T*_0_ lines (Fig. 3b and Supplementary Fig. 13). By contract, the least performing multiplex systems (C, E, F, and J) all used the Pol III promoter OsU6 and at most resulted in mutations at one site (Fig. 3b and Supplementary Fig. 13). These results suggest Pol III promoter-based systems are not suitable for expressing multiple crRNAs, even though they were previously used in humans^8^ and plants^9^; this may be because Pol III promoters are evolved to transcribe tRNA and other small RNAs in nature and not suitable for producing long transcripts.

### Comparison of seven refined multiplex Cas12a genome editing systems

We next focused on seven refined multiplexed LbCas12a systems that primarily used Pol II promoters: B, D, G, H, M, I, and L (Fig. 3a). We included two additional STU systems, M and L, with M using an extra direct repeat (DR) at the end of the crRNA array and L using HH and HDV to flank the crRNA array. This time, we used four crRNAs to target four different genes. For the seven systems, we generated 36, 60, 36, 50, 30, 33, and 36 *T*_0_ lines, respectively. While biallelic editing at *OsPDS* and *OsROC5* could be detected based on phenotype (Fig. 3c), we relied on restriction fragment length polymorphism (RFLP) assays and Sanger sequencing to genotype the edits (Fig. 3d and Supplementary Figs. 14–20). We first calculated editing efficiencies at each target site for all the multiplex systems. System B stood out as the most efficient multiplex system, resulting in nearly 100% biallelic mutagenesis at all target sites (Fig. 3d and Supplementary Fig. 14). More impressively, all *T*_0_ lines carried biallelic mutations at three target genes: *OsPDS*, *OsROC5*, and *OsmiR528*. The high editing efficiency of system B was further confirmed with amplicon sequencing using the Hi-TOM platform^46^ (Supplementary Figs. 21 and 22). The second tier of high-efficiency multiplex systems includes systems D, M, and L, where editing frequencies were over 50% for every target site. The remaining three systems, I, H, and G, had relatively low multiplex editing frequencies.

To gain further insights about the multiplexing ability of these systems, we calculated their editing frequencies when editing defined numbers of genes (4, 3, 2, and 1) simultaneously. System B again stood out since it achieved the highest multiplexed editing when targeting four genes (Fig. 3e). The second-tier systems, D, M, and L, were clustered together with 40–50% quadruple editing frequencies (Fig. 3e). A similar trend has been observed when we calculated the biallelic editing efficiencies (Fig. 3f). We further applied the highly efficient multiplex system B to Mb2Cas12a and found 8 out of 11 *T*_0_ lines carried biallelic edits at all four target sites (Fig. 3g and Supplementary Fig. 23). We then used Mb2Cas12a-RVRR coupled with multiplex system B to target seven sites with canonical and altered PAMs simultaneously. All sites were edited with efficiencies ranging from 5.1 to 31.1% in rice protoplasts (Supplementary Fig. 24 and Supplementary Data 1). Taken together, we identified system B, a dual Pol II promoter and tandem HH-crRNA-HDV system, as the most efficient multiplex Cas12a system (Fig. 3a).

To investigate whether multiplexed genome editing by Mb2Cas12a can cause off-target mutagenesis, we amplified and sequenced 27 potential off-target sites with four or fewer mismatches or bulges within the protospacers in two transgenic *T*_0_ lines. No off-target mutations were detected (Supplementary Table 3), suggesting high specificity of Mb2Cas12a during multiplexed genome editing.

### Multitrait engineering within one generation

**Fig. 4:**
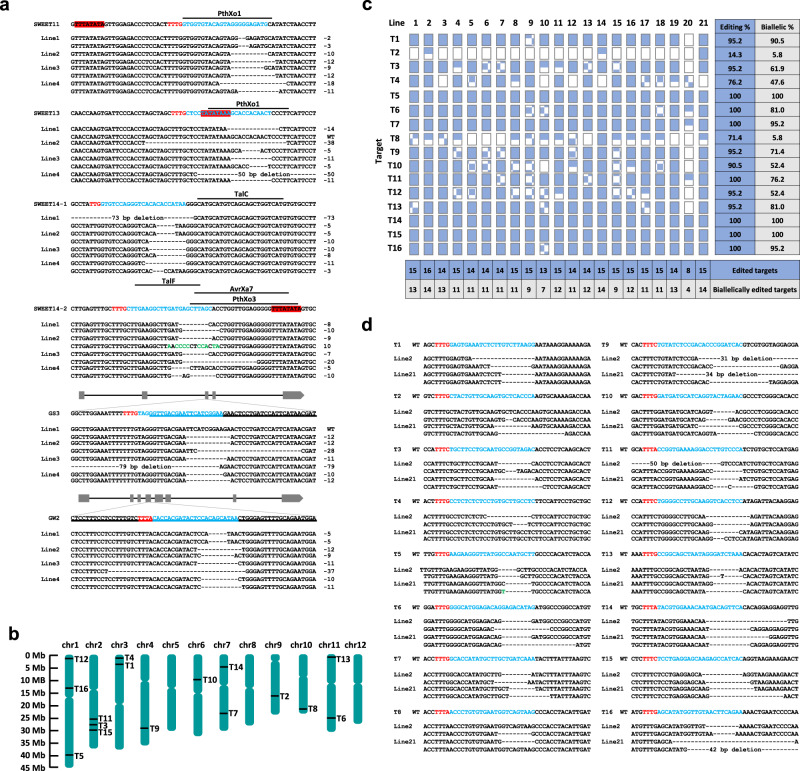
Highly efficient large-scale biallelic genome editing in rice. **a** Simultaneous editing of six sites by Mb2Cas12a for multiplexed engineering of quantitative traits in rice. Genotypes of four independent *T*_0_ lines are shown, indicating all the target sites are nearly edited biallelically. The PAM sites are highlighted in red and the target sequences are highlighted in blue. The TATA boxes are painted in red. The TAL effector binding sites in the promoters of the *SWEET* genes are indicated by solid black lines above the sequences. Exons of *GS3* and *GW2* are underlined. **b** Schematics of simultaneous editing by LbCas12a at 16 different target sites (T1–T16) across nine rice chromosomes (chr). **c** Genotypes of 21 tested *T*_0_ lines at 16 target sites, with wild type denoted as empty rectangles, monoallelic mutants denoted as half-filled rectangles, biallelic mutants denoted as fully filled rectangles, and chimeras denoted as doted rectangles. **d** Genotypes of *T*_0_ Line 2 and Line 21 at 16 target sites, where 14 sites were biallelically edited in both lines.

We next applied the multiplex system B for simultaneous editing of five trait genes to enhance yield and blight disease resistance in rice. We generated a Cas12a vector multiplexing six crRNAs to edit the coding sequences of *OsGS3* (*GRAIN SIZE3*)^47^ and *OsGW2* (*GRAIN WIDTH and WEIGHT2*)^48^, as well as the promoters of three *SWEET* genes, *SWEET11*, *SWEET13*, and *SWEET14*. *OsGS3* and *OsGW2* are negatively correlated with grain size, and grain width and weight, respectively. Transcription activator-like effectors (TALEs) from *Xanthomonas oryzae* pv. *oryzae* can bind to the *SWEET* promoter region, causing bacterial leaf blight^49^. Mb2Cas12a was chosen since one target site contains a VTTV PAM. Four crRNAs were designed to destroy all the TALE binding sites within the *SWEET* promoters, including PthXo1, TalC, TalF, AvrXa7, and PthXo3^49,50^ (Fig. 4a). High biallelic editing efficiencies at all six target sites in 8 out of 10 individual *T*_0_ lines were observed (Fig. 4a and Supplementary Fig. 25). All the targeted TAL effector binding sites were mutated by large deletions in all these lines, except for TalC. Expression of the *SWEET* genes was not compromised in these promoter-edited lines (Supplementary Fig. 26), indicating that the promoter function of the *SWEET* genes was not disrupted. These results reinforced the practical application of this promoter editing strategy as demonstrated with TAL effector nuclease (TALEN)^51^ and Cas9^49^. Simultaneous editing of *OsGS3* and *OsGW2* was also achieved in these lines (Fig. 4a), which is expected to contribute to increased yield^52^. Off-target analysis of two multiplex edited *T*_0_ lines showed no edits at potential off-target sites (Supplementary Table 3). Together, we demonstrated the use of a multiplexed Mb2Cas12a system for simultaneous biallelic editing of different trait genes in rice within one generation.

### Large-scale biallelic genome editing

Since the multiplex system B showed nearly 100% biallelic editing frequencies at four and six target sites (Figs. 3 and 4a), we would like to see how it performs when scaled up. To this end, we generated a single LbCas12a T-DNA vector to simultaneously target 16 genomic sites across nine chromosomes in rice (Fig. 4b). We assessed editing at all sites in 21 *T*_0_ lines with RFLP assays and the Hi-TOM sequencing platform^46^ (Supplementary Figs. 27–31). We found 20 out of 21 *T*_0_ lines contained edits at 13 or more sites with seven or more biallelic edits (Fig. 4c). Eleven *T*_0_ lines had edits at 15 sites, and one line (Line 2) had all 16 sites edited (Fig. 4c). The average editing and biallelic editing efficiencies for each target were 89.3% and 69.8%, respectively, indicating a high multiplexed editing frequency. These data suggest that multiplex system B can enable highly efficient large-scale biallelic genome editing in rice, with the ability to edit 16 sites and biallelically edit 14 sites simultaneously (Fig. 4d).

### Multiplexed transcriptional repression by compact STU dCas12a-SRDX systems

Previously, we demonstrated transcriptional repression of single genes in plants with dCas12a-SRDX^16,24^. The STU systems represent the most compact expression systems for simultaneous and coordinated repression of multiple genes, which can potentially reduce the difficulties in cloning and transformation due to large constructs, as well as avoid gene silencing. Therefore, we chose two top performing STU systems, D and M (Fig. 3), for multiplexed transcriptional repression based on dLbCas12a-SRDX. First, we tested the systems in rice by simultaneously targeting four genes. For each system, two constructs were designed, and each construct contained four crRNAs targeting the promoters of four chosen genes (Supplementary Fig. 32a). Simultaneous transcriptional repression was observed in transformed rice protoplasts, especially when antisense strand was targeted with system M with the target genes repressed to ~20% of the control level (Supplementary Fig. 32b). We further tested the systems in *Arabidopsis* by targeting two tandemly arrayed genes, *At3g48090* and *At3g48080*, which encode two *EDS1* homologs (Supplementary Fig. 32c). Simultaneous transcriptional repression of both genes was observed in multiple *T*_1_ lines with either the D or M STU system (Supplementary Fig. 32d, e), and the repression effects were transmitted into the *T*_2_ generation (Supplementary Fig. 32f).

## Discussion

As a promising genome editing system, CRISPR-Cas12a is constantly under improvement in areas such as editing activaties^15,26^, PAM requirements^13,15^, and multiplexing capabilities^8,37^. Only three Cas12a orthologs, LbCas12a, FnCas12a, and AsCas12a, have been previously applied for plant genome editing^9,16–30^. AsCas12a, which has low nuclease activities at lower temperatures^24,43^, could be improved by protein engineering^15^ and directed evolution (e.g., Alt-R Cas12a (Cpf1) Ultra, IDT). Hence, it is important to identify more functional Cas12a orthologs, as they can directly or indirectly (e.g., through protein engineering) contribute to the expansion and improvement of the Cas12a toolbox. Toward this, four Cas12a orthologs, MbCas12a, Mb3Cas12a, HkCas12a, and ErCas12a, were recently demonstrated in mammalian cells^14,39,41,44^. In this study, we identified eight Cas12a orthologs that can mediate genome editing in rice. Six of them, ErCas12a, Lb5Cas12a, BsCas12a, Mb2Cas12a, TsCas12a, and MbCas12a, showed robust editing by generating biallelic mutants in rice. Mb2Cas12a can efficiently edit relaxed VTTV PAM sites, and its RVR variant can robustly edit TATV PAM sites in rice. Moreover, Mb2Cas12a-RVRR can edit TTTV, VTTV, TATV, TYCV, CCCV, and CTCV PAM sites, greatly expanding the targeting scope. These functional Cas12a orthologs have also provided a rich Cas12a resource for future improvement through protein engineering.

Despite many previous efforts on developing multiplex Cas12a systems in plants^9,25,53–57^, a highly efficient multiplexed biallelic editing system remains elusive. For example, Cas12a mediated multiplexed editing was previously reported in rice at limited sites with biallelic editing frequencies up to 28%^9^, 33%^57^, 43%^58^, 67%^55^, respectively. By comparing 12 multiplexed Cas12a systems, here we successfully identified one multiplex system that enabled high-efficiency large-scale biallelic genome editing. This system B uses a Pol II promoter to express a tandem HH-crRNA-HDV array. Using system B, we achieved simultaneous biallelic editing at 14 independent loci in the rice genome at a high frequency. We did not observe decreased editing efficiency when increasing the distance between the promoter and the crRNA (T1 is the closest and T16 is the farthest to the promoter) (Fig. 4c). We envision that its multiplexing capacity could be far beyond this, which requires future testing. Further, we applied two compact STU dCas12a-SRDX systems for multiplexed transcriptional repression in rice and *Arabidopsis*, providing an upgrade from singular transcriptional repression systems based on Cas12a^16,24^. Assembly of these useful multiplexed Cas12a systems is based on modular Golden Gate and Gateway vectors (Supplementary Table 4) and streamlined cloning strategies (Supplementary Fig. 33).

In conclusion, these systems have greatly expanded the targeting scope of Cas12a, and they collectively represent very powerful tools for crop breeding and engineering. A toolbox for using these Cas12a reagents has been established and is available to all researchers.

## Methods

### Plant material and growth condition

Rice (*Oryza sativa*) plants used in this study were the Japonica cultivar *Nipponbare* and *Kitaake*. Seedlings of 14–16-day old grown on ½ MS medium^59^ in dark at 28 °C were used for protoplast isolation^60^. Rice calli induced from mature rice embryos, were cultured on the N6D medium^61^ under light at 32 °C and used for rice stable transformation. *Arabidopsis thaliana* (Col-0) plants grown at 22 °C were used for the gene repression study.

### Vector construction

All vectors used in this study were constructed based on a three-way Gateway cloning system. T-DNA vectors used for single target editing were constructed using the previously described method^60,62^. To express Cas12a nuclease in attL1-attR5 entry vectors, three gBlocks were synthesized using rice codon optimized sequences for each Cas12a ortholog and assembled with the pYPQ230 (LbCas12a) backbone using the NEBuilder HiFi DNA Assembly Cloning Kit (New England BioLabs). To generate RVR and RVRR variants, Mb2Cas12a-v1 and Mb2Cas12a-v2, mutations were introduced using the Q5^®^ Site-Directed Mutagenesis Kit (New England BioLabs). To express crRNA in attL5-attL2 entry vectors, duplexed oligonucleotides were phosphorylated, annealed and ligated into pYPQ141-ZmUbi-RZ-Lb (for LbCas12a) and pYPQ141-ZmUbi-RZ-Fn (for all other Cas12a orthologs) at the *Esp3*I (*BsmB*I) site. These two types of entry vectors were further assembled with the destination vector pYPQ203^63^ through LR reactions.

T-DNA vectors used for multiplexed genome editing and gene transcriptional regulation were constructed using the following method. The attL1-attR5 entry vectors included previously described pYPQ230 and pYPQ284 (Mb2Cas12a). To generate pYPQ230-STU to express Cas12a and its crRNAs as a single transcript unit (STU), the *NOS* terminator in pYPQ230 was replaced by a polyA signal at the *Aat*II and *BspE*I sites. Similarly, to generate pYPQ233-STU, the *NOS* terminator in pYPQ233 (LbCas12a-SRDX) was replaced by a polyA signal. To express four crRNAs in attL5-attL2 entry vectors, the cloning method for each multiplexing system is as follows: (A) crRNA1 and 3 were cloned into pYPQ131C-RZ-Lb (Addgene #134347) while crRNA2 and 4 were cloned into pYPQ131D-RZ-Lb (Addgene #134348) at the *Esp3*I site^33^. All four crRNA expression cassettes were PCR amplified using primers flanked by *Bsa*I recognition and cutting sites that are compatible with Golden Gate assembly. Four PCR products were assembled with recipient vector pYPQ144 (Addgene #69296)^63^ using Golden Gate reactions. (B–D) To make Golden Gate recipient vector pYPQ144-pT, a polyT signal was inserted into pYPQ144 at the *Spe*I and *EcoR*I sites. To make Golden Gate recipient vector pYPQ144-ZmUbi-pT, pZmUbi was cut off from pYPQ141-ZmUbi-RZ-Lb with *Afl*II and *BamH*I and cloned into pYPQ144-pT. To make Golden Gate recipient vector pYPQ144C-pT, OsU6 promoter was PCR amplified from pYPQ131C (Addgene #69284)^63^ and cloned into pYPQ144-pT at the *Xba*I and *BamH*I sites. The crRNA1–4 were cloned into pYPQ131-STU-Lb/Fn, pYPQ132-STU-Lb/Fn, pYPQ133-STU-Lb/Fn, and pYPQ134-STU-Lb/Fn, respectively. These four vectors were assembled with recipient vectors pYPQ144-ZmUbi-pT, pYPQ144C-pT, and pYPQ144-pT using Golden Gate reactions for multiplexing systems B, C, and D, respectively. (E) To generate an empty vector for crRNA expression using the tRNA and ribozyme hybrid system, a gBlock containing OsU6 promoter, tRNA, crRNA direct repeat and cloning site, as well as the HDV ribozyme was synthesized and cloned into pYPQ131C at the *BamH*I and *Nco*I sites. This vector is named as pYPQ131C-tZ-Lb. The crRNA1–4 were then cloned into pYPQ131C-tZ-Lb, and all four crRNA expression cassettes without promoters were PCR amplified using primers flanked by *Bsa*I recognition and cutting sites that are compatible with Golden Gate assembly. Four PCR products were assembled with recipient vector pYPQ144-pT using Golden Gate reactions. (F and G) To generate an empty vector for crRNA expression using the tRNA and ribozyme combined system, a gBlock containing OsU6 promoter, tRNA, HH ribozyme, crRNA direct repeat and cloning site, as well as the HDV ribozyme was synthesized and cloned into pYPQ131C at the *BamH*I and *Nco*I sites. This vector is named as pYPQ131C-tDZ-Lb. The crRNA1–4 were cloned into pYPQ131C-tDZ-Lb, and all four crRNA expression cassettes without promoters were PCR amplified using primers flanked by *Bsa*I recognition and cutting sites that are compatible with Golden Gate assembly. Four PCR products were assembled with recipient vector pYPQ144C-pT and pYPQ144-pT using Golden Gate reactions for multiplexing systems F and G, respectively. (H) The CRISPR array driven by OsU6 promoter as well as a polyT signal at the end was synthesized and ligated into pYPQ144-pT at the *EcoR*I and *BamH*I sites. (M) The CRISPR array without promoter but with an extra crRNA direct repeat, and a polyT signal at the end was synthesized and ligated into pYPQ141-pT at the *EcoR*I and *BamH*I sites. (I and L) CRISPR array flanked by HH and HDV ribozymes as well as a polyT signal at the end was synthesized and ligated into pYPQ144-ZmUbi-pT and pYPQ141-pT at *EcoR*I and *BamH*I sites for multiplexing systems I and L, respectively. (J) CRISPR array flanked by HH and HDV ribozymes, driven by OsU6 promoter as well as a polyT signal at the end, was synthesized and ligated into pYPQ144-pT at the *EcoR*I and *BamH*I sites. The attL1-attR5 entry vectors used to edit six and 16 sites were assembled using the same method described previously for multiplexing system B, followed by a higher order assembly using restriction digestion and ligation. The attL1-attR5 entry vectors and attL5-attL2 entry vectors were further assembled with the destination vector pYPQ203 through LR reactions.

All vectors used for single target editing, as well as for multiplexed genome editing and gene transcriptional regulation using top performing multiplexing systems (B, D, I, L, and M), were deposited to Addgene. The Addgene plasmid numbers can be found in Supplementary Table 4. Information for all primers and gBlocks used in this study can be found in Supplementary Data 2. Information for all the T-DNA vectors used in this study can be found in Supplementary Data 3. A toolbox for multiplexed genome engineering using Cas12a has been established in this study and the detailed user manual is included in Supplementary Methods.

### Rice protoplast transformation

Rice protoplasts were isolated and transformed according to a previously published protocol^60^. Briefly, 14–16-day old rice leaves grown in the dark were cut into 0.5–1.0 mm strips and incubated in the enzyme solution at 28 °C for 8 h without light. The digested cells were filtered by 75 μm cell strainer and washed by W5 buffer twice. 30 μg plasmid DNA was mixed with 200 μl protoplasts (2 × 10^6^ ml^−1^) re-suspended in MMG buffer (400 μl protoplasts were used for the gene repression experiment). Equal amount of polyethylene glycol (PEG) transformation buffer was then added, and the entire mixture was incubated for 30 min at room temperature. The reactions were stopped by adding 900 μl W5 buffer. Protoplasts were collected by centrifugation and transferred into 12-well culture plates. Plates were incubated at 32 or 22 °C in dark for 2 days. The protoplasts were collected by centrifugation for DNA or RNA extraction. Protoplasts transformed with water were used as the wild type (WT) control for all genome editing experiments.

### Rice stable transformation

Rice was transformed using the *Agrobacterium*-mediated method as described in the published protocols with slight modifications^61,64,65^. Briefly, *Agrobacterium tumefaciens* strain EHA105 harboring binary vectors was used to inoculate rice calli. Inoculated calli were co-cultured with *Agrobacterium* for 3 days, washed and moved to selection medium containing 50 mg l^−1^ hygromycin. After 4 weeks, resistant calli were moved to regeneration medium I to induce shoot growth. Small shoots were further transferred to regeneration medium II to obtain full transgenic plants. DNA was extracted from young leaves of *T*_0_ plants using the CTAB method for genotyping^66^.

### Mutation analysis by RFLP and SSCP

To analyze mutation results using the restriction fragment length polymorphism (RFLP) method, the targeted genomic regions were amplified, and the PCR products were digested with restriction enzymes whose cutting sites are overlapping with the expected editing sites. Digested products were visualized on 2% TAE agarose gels. Mutation frequencies were quantified based on band intensity using Image Lab™ Software (Bio-Rad Laboratories) and ImageJ (https://imagej.nih.gov/ij/) for protoplast assays. For stable transgenic rice lines, any samples with undigested bands were considered edited, while any samples without detectable digested bands were considered biallelically edited in the RFLP analysis. To analyze mutation results in transgenic rice plants using the single-strand conformational polymorphism (SSCP) method^67^, amplicons were denatured for 5 min at 95 °C and immediately put on ice to minimize self-annealing. Denatured PCR amplicons were electrophoresed on 15 % non-denaturing polyacrylamide gels at 45 mA, 120–200 V. After 6 h, polyacrylamide gels were stained using argentation.

### Sanger sequencing and deep sequencing

PCR amplicons from stable transgenic rice were subjected to Sanger sequencing. DNA sequences were decoded using DSDecodeM^68^. PCR amplicons with sequencing barcodes generated from protoplast assay were first submitted for quality check, followed by sequencing using an Illumina HiseqXten-PE150 system. Sequencing data were then analyzed by CRISPRMatch^69^ for editing frequencies and profiles. For off-target analysis, amplicons from Line 10 (multiplexed genome editing by Mb2Cas12a targeting four sites) and Line 1 (multiplexed genome editing by Mb2Cas12a targeting six sites for quantitative traits) were pooled together, while amplicons from Line 11 (multiplexed genome editing by Mb2Cas12a targeting four sites) and Line 2 (multiplexed genome editing by Mb2Cas12a targeting six sites for quantitative traits) were pooled together. Potential off-target sites were sequenced using the Illumina HiSeq2500 system and analyzed using CRISPResso2^70^. To assess the editing results for multiplexed genome editing at 4 sites and 16 sites using system B, PCR amplicons were barcoded using the Hi-TOM^46^ primers and pooled into two and four samples for Illumina HiSeq2500, respectively. Data were analyzed using the Hi-TOM online tool (https://doi.org/www.hi-tom.net/hi-tom/) with a 12.5% filter threshold.

### *Arabidopsis* stable transformation

*Arabidopsis thaliana* wild type plants *Col-0* were transformed with *Agrobacterium tumefaciens* GV3101 using the flora dip method^71^. T_1_ generation seeds were collected and sterilized with 50% bleach and 0.05% Tween. After 3 days of vernalization at 4 °C, seeds were plated on ½ MS medium supplemented with 15 mg l^−1^ hygromycin. After a week, hygromycin resistant plants were transferred to ½ MS medium to recover for another week. Individual plants were treated as single transgenic lines. Plants transformed with a *GUS* gene and a hygromycin resistance gene were used as controls. Leaf tissue was collected from each line along with control plants for qRT-PCR analysis. Seeds collected from these lines were treated using the same method to obtain the T_2_ generation plants. Three plants of each line were used for qRT-PCR analysis.

### Gene expression analysis using qRT-PCR

Total RNA from rice and *Arabidopsis* leaf tissue or rice protoplast was extracted using TRIzol™ Reagent (Thermo Fisher Scientific) following the manufacturer’s instructions with slight modifications. DNA was then removed with DNase I (New England BioLabs) and the complementary DNA (cDNA) was synthesized using the SuperScript^TM^ III First-Strand Synthesis System (Thermo Fisher Scientific). Applied Biosystems™ SYBR™ Green PCR Master Mix (Thermo Fisher Scientific) was used for qRT-PCR. Three technical replicates were carried out for each sample using a CFX96 Touch™ Real-Time PCR Detection System (Bio-Rad Laboratories). *AtEf1α* was used as the reference gene for *Arabidopsis* and *OsTubulin* was used as the reference gene for rice. Protoplasts transformed with the T-DNA vector containing LbCas12a without crRNAs were used as controls for rice. Relative expression level of each gene was calculated using the 2^−ΔΔCt^ method.

### Genome-wide PAM analysis

The rice cultivar Nipponbare genome sequence (Oryza_sativa_nipponbare_v7.0_all.con) was obtained from the Rice Genome Annotation Project (rice.plantbiology.msu.edu/pub/data/Eukaryotic_Projects/o_sativa/annotation_dbs/pseudomolecules/). PAM sequence and its reverse complementary sequence were used to search the FASTA files using a regular expression search in Perl (version 5.26).

### Statistical analysis

Pearson’s *χ*^2^ test was used to show whether there is a significant difference between the expected frequencies and the observed frequencies of edits in transgenic rice *T*_1_ populations when *α* = 0.05. Student’s *t*-test was used for pairwise comparison. One asterisk (*p* < 0.05) and two asterisks (*p* < 0.01) indicate significant differences between two treatments. Tukey’s Honest Significant Difference (HSD) test was used for multiple comparisons. Treatments with the same letter are not significantly different when *α* = 0.05.

### Reporting summary

Further information on research design is available in the Nature Research Reporting Summary linked to this article.

## Supplementary information

Supplementary Information

Description of Additional Supplementary Files

Supplementary Data 1

Supplementary Data 2

Supplementary Data 3

Reporting Summary

## Data Availability

Next-generation sequencing (NGS) data have been deposited to the National Center for Biotechnology information (NCBI) database under Sequence Read Archive (SRA) BioProject “PRJNA595844”. The 35 Golden Gate and Gateway compatible vectors for the new Cas12a toolbox are available at Addgene (Supplementary Table 4). Source data are provided with this paper. Any other relevant data are available from the authors upon reasonable request. Source data are provided with this paper.

## Source data

Source Data
