# Supplementary material for: Expanding the scope of plant genome engineering with Cas12a orthologs and highly multiplexable editing systems
Source: Nat Commun. 2021 Mar 29;12:1944. doi: 10.1038/s41467-021-22330-w (PMC8007695; doi:10.1038/s41467-021-22330-w)
Supplement: Supplementary file 1 — Supplementary Information [file 41467_2021_22330_MOESM1_ESM.pdf]

Expanding the scope of plant genome engineering with Cas12a orthologs and highly multiplexable editing systems

Yingxiao Zhang[1,ǂ], Qiurong Ren[2,ǂ], Xu Tang[2,ǂ], Shishi Liu[2], Aimee A. Malzahn[1], Jianping Zhou[2], Jiaheng Wang[2], Desuo Yin[1,3], Changtian Pan[1], Mingzhu Yuan[2], Lan Huang[2], Han Yang[2], Yuxin Zhao[2], Qing Fang[2], Xuelian Zheng[2], Li Tian[2], Yanhao Cheng[1,4], Ysa Le[1], Bailey McCoy[1], Lidiya Franklin[1], Jeremy D. Selengut[5], Stephen M. Mount[6], Qiudeng Que[7], Yong Zhang[2*], Yiping Qi[1,8*]

[1]Department of Plant Science and Landscape Architecture, University of Maryland, College Park, Maryland 20742, USA;
[2]Department of Biotechnology, School of Life Science and Technology, Center for Informational Biology, University of Electronic Science and Technology of China, Chengdu 610054, China;
[3]Food Crop Institute, Hubei Academy of Agricultural Sciences, Wuhan, Hubei 430064, China;
[4]College of Agriculture, Nanjing Agricultural University, Nanjing, Jiangsu 210095, China;
[5]Center for Bioinformatics and Computational Biology, University of Maryland, College Park, Maryland 20742, USA
[6]Department of Cell Biology and Molecular Genetics, University of Maryland, College Park, Maryland 20742, USA;
[7]Syngenta, Research Triangle Park, North Carolina 27709, USA;
[8]Institute for Bioscience and Biotechnology Research, University of Maryland, Rockville, Maryland 20850, USA.

[ǂ]These authors contributed equally to this work.

***Corresponding authors:**

Yiping Qi, Email: yiping@umd.edu
Yong Zhang, Email: zhangyong916@uestc.edu.cn

Supplementary Methods

Supplementary Figures

Supplementary Tables

Supplementary Methods

Cas12a multiplexing toolbox user manual

Cas12a multiplexing toolbox is designed to assemble T-DNA vectors for multiplexed genome editing and transcriptional repression in plants. This toolbox is designed based on a three-way Gateway cloning system. The attL5-attL2 entry vectors are used to express multiple crRNAs and the attL1-attR5 entry vectors are used to express Cas12a or Cas12a-repressor fusion proteins. The construction of the attL5-attL2 entry vectors is described in **STEP 1** and **2** and the final assembly of two entry vectors with the destination vector is described in **STEP 3**. Gateway™ LR Clonase™ II Enzyme Mix and *Esp3*I (*BsmB*I) are from Thermo Fisher Scientific. All other reagents are from New England Biolabs. Equivalent reagents can also be used for this toolbox.

STEP 1 Choose multiplexing system and prepare crRNAs

If multiplexing system B or D is chosen, please follow **STEP 1A**.

If multiplexing system I, L or M is chosen, please follow **STEP 1B**.

STEP 1A

- Synthesize crRNA as DNA oligonucleotides as follows: forward oligo: 5'-TAGATNNNNNNNNNNNNNNNNNNNNNNN-3'; reverse oligo: 5'-GGCCNNNNNNNNNNNNNNNNNNNNNNNA-3'). Phosphorylate and anneal oligos using the following recipe:

Oligonucleotides phosphorylation

Component	Volume
crRNA forward oligo (100 μM)	1 μl
crRNA reverse oligo (100 μM)	1 μl
T4 Ligase Buffer (10 X)	1 μl
T4 PNK (10 U μl^{-1})	0.5 μl (5 U)
Nuclease-free Water	6.5 μl
Total	10 μl

- Incubate reactions at 37℃ for 30 min and place them in boiling water. Let reactions cool down to room temperature to allow two oligos to anneal together.
- At the same time, digest crRNA cloning vectors using the following recipe:

Empty crRNA cloning vector digestion

Component	Volume
pYPQ131/2/3/4-STU-As/Fn/Lb	20 µl (2 µg)
Buffer Tango (10 X)	5 µl
DTT (10 mM)	5 µl
*Esp*3I (*BsmB*I) (10 U µl^{-1})	2 µl (20 U)
Nuclease-free Water	18 µl
Total	50 µl

- Incubate reactions at 37℃ overnight (preferred) followed by gel purification to remove undigested plasmid. Please choose the corresponding crRNA direct repeat for the Cas12a that will be used for assembly. If less than four crRNAs will be used, only digest empty crRNA cloning vectors for those crRNAs. For instance, if only two crRNAs will be used, only digest pYPQ131-STU-As/Fn/Lb and pYPQ132-STU-As/Fn/Lb. If more than four crRNAs will be used, divide them into groups of four or less. The crRNAs from each group can be assembled together first and ligated with other groups in **STEP 2**.
- Ligate crRNAs to digested empty crRNA cloning vectors using the following recipe:

crRNA expression vector ligation

Component	Volume
*Esp*3I (*BsmB*I) linearized pYPQ131/2/3/4-STU-As/Fn/Lb	2 µl (50 ng)
Diluted annealed oligos (1:200 dilution)	2 µl
T4 DNA Ligase Buffer (10X)	2 µl
T4 DNA Ligase (400 U µl^{-1})	1 µl (400 U)
Nuclease-free Water	13 µl
Total	20 µl

- Incubate reactions at room temperature for two hours followed by *E.coli* transformation. Plate *E.coli* on LB medium supplemented with 10 mg l^{-1} tetracycline. Confirm correct clones by Sanger sequencing.

STEP 1B

- If multiplexing system I or L is chosen, synthesize DNA fragment using the following configuration:

  ***BamH*I site—HH ribozyme—direct repeat—crRNA1—direct repeat—crRNA2············ direct repeat—crRNAn—HDV ribozyme—*EcoR*I site**

- If multiplexing system M is chosen, synthesize DNA fragment using the following configuration:

BamHI site—direct repeat—crRNA1—direct repeat—crRNA2··········· direct repeat—crRNAn—direct repeat—EcoRI site

STEP 2 Assemble crRNAs

If multiplexing system B or D is chosen, please follow **STEP 2A**.

If multiplexing system I, L or M is chosen, please follow **STEP 2B**.

STEP 2A

- Assemble all crRNAs with the recipient vector using the Golden Gate reaction as follows:

Golden Gate reaction to assemble four crRNAs

Component	Volume
pYPQ131-STU-As/Fn/Lb-crRNA1	1 μl (100 ng)
pYPQ132-STU-As/Fn/Lb-crRNA2	1 μl (100 ng)
pYPQ133-STU-As/Fn/Lb-crRNA3	1 μl (100 ng)
pYPQ134-STU-As/Fn/Lb-crRNA4	1 μl (100 ng)
pYPQ144-ZmUbi-pT or pYPQ144	1 μl (100 ng)
T4 DNA Ligase Buffer (10 X)	2 μl
*Bsa*I	2 μl (20 U)
T4 DNA Ligase (400 U μl^{-1})	2 μl (800 U)
Nuclease-free Water	9 μl
Total	20 μl

- Incubate reactions in a thermocycler with the following procedure:

Temperature (℃)	Time (min)	Cycles
37	5	10
16	10	
50	5	1
80	5	1

- Transform reactions into *E.coli* and plate on LB medium supplemented with 50 mg l^{-1} spectinomycin. Before plating, spread 50 μl of 20 mg ml^{-1} X-gal and 50 μl of 0.1 M IPTG on LB plates for blue-white screen. Confirm correct clones by digestion and Sanger sequencing. Use pYPQ144-ZmUbi-pT for multiplexing system B and pYPQ144 for multiplexing system D. If less than four crRNAs will be used, choose different recipient vectors. For instance, if only two crRNAs will be used, use pYPQ142-ZmUbi or pYPQ142 as the recipient vector.

- If more than four crRNAs will be used, divide them into groups of four or less. The crRNAs from each group can be assembled together using the same method, followed by a higher order assembly using restriction digestion and ligation. Here is an example to assemble 16 crRNAs using multiplexing system B and D:

<table>
<tr><th></th><th>Multiplexing system B</th><th>Multiplexing system D</th></tr>
<tr><td>STEP 1A</td><td>Divide crRNAs into four groups, each group has four crRNAs.
Clone crRNAs into pYPQ131/2/3/4-STU-As/Fn/Lb.</td><td>Divide crRNAs into four groups, each group has four crRNAs.
Clone crRNAs into pYPQ131/2/3/4-STU-As/Fn/Lb.</td></tr>
<tr><td>STEP 2A</td><td>Assemble crRNAs from each group into one vector using Golden Gate reactions. crRNA1-4 will be assembled into pYPQ144-ZmUbi-pT. crRNAs from other groups will be assembled into pYPQ144. The following four vectors will be obtained:
pYPQ144-ZmUbi-crRNA1-4
pYPQ144-crRNA5-8
pYPQ144-crRNA9-12
pYPQ144-crRNA13-16</td><td>Assemble crRNAs from each group into one vector using Golden Gate reactions. Use pYPQ144 as the recipient vector for all groups. The following four vectors will be obtained:
pYPQ144-crRNA1-4
pYPQ144-crRNA5-8
pYPQ144-crRNA9-12
pYPQ144-crRNA13-16</td></tr>
<tr><td>STEP 2A Higher order assembly</td><td>• Cut off crRNA9-12 from pYPQ144-crRNA9-12 using NcoI and SpeI and ligate into pYPQ144-crRNA13-16 at the NcoI and XbaI sites to make pYPQ-crRNA9-16.
• Cut off crRNA5-8 from pYPQ144-crRNA5-8 using NcoI and SpeI and ligate into pYPQ-crRNA9-16 at the NcoI and XbaI sites to make pYPQ-crRNA5-16.
• Cut off ZmUbi-crRNA1-4 from pYPQ144-ZmUbi-crRNA1-4 using BbsI and SpeI and ligate into pYPQ144-crRNA5-16 at the BbsI and XbaI sites to make pYPQ-ZmUbi-crRNA1-16.</td><td>• Cut off crRNA9-12 from pYPQ144-crRNA9-12 using NcoI and SpeI and ligate into pYPQ144-crRNA13-16 at the NcoI and XbaI sites to make pYPQ-crRNA9-16.
• Cut off crRNA5-8 from pYPQ144-crRNA5-8 using NcoI and SpeI and ligate into pYPQ-crRNA9-16 at the NcoI and XbaI sites to make pYPQ-crRNA5-16.
• Cut off crRNA1-4 from pYPQ144-crRNA1-4 using NcoI and SpeI and ligate into pYPQ144-crRNA5-16 at the NcoI and XbaI sites to make pYPQ-crRNA1-16.</td></tr>
</table>

STEP2B

- If multiplexing system I is chosen, digest synthesized DNA fragment using *BamH*I and *EcoR*I and ligate into pYPQ144-ZmUbi-pT.

- If multiplexing system L or M is chosen, digest synthesized DNA fragment using *BamH*I and *EcoR*I and ligate into pYPQ144.

STEP3 Assemble T-DNA vectors

- Assemble attL5-attL2 entry vectors and attL1-attR5 entry vectors with the destination vector pYPQ203 by LR reactions as follows:

LR reaction

**Component**	Volume
attL1-attR5 entry vector	1.5 µl (150 ng)
attL5-attL2 entry vector	1 µl (100 ng)
Destination vector pYPQ203	2 µl (200 ng)
LR Clonase II	1 µl
Total	5.5 µl

- Incubate reactions at room temperature for at least one hour. Transform reactions into *E.coli* and plate on LB medium supplemented with 50 mg l^{-1} kanamycin. Final T-DNA vectors can be confirmed by *EcoR*I digestion.
- The destination vector harbors a promoter (pZmUbi) for Cas12a expression and a selective marker (*HygR*) for plant regeneration. These components can be modified to suit different plant species and transformation methods.
- Please choose attL1-attR5 entry vectors according to the following instructions:

Purpose	Multiplexing system	Cas12a	attL1-attR5 entry vectors
**Genome editing**	B and I	LbCas12a	pYPQ230
		AsCas12a	pYPQ220
		FnCas12a	pYPQ239
		Mb2Cas12a	pYPQ284
	D, L and M	LbCas12a	pYPQ230-STU
		FnCas12a	pYPQ239-STU
**Transcriptional repression**	B and I	LbCas12a	pYPQ233
		AsCas12a	pYPQ223
	D, L and M	LbCas12a	pYPQ233-STU
		AsCas12a	pYPQ223-STU

Please see **Supplementary Table 4** for the Addgene plasmid numbers of all vectors. Please see **Supplementary Fig. 33** for an illustration of the toolbox to assemble four crRNAs.

a

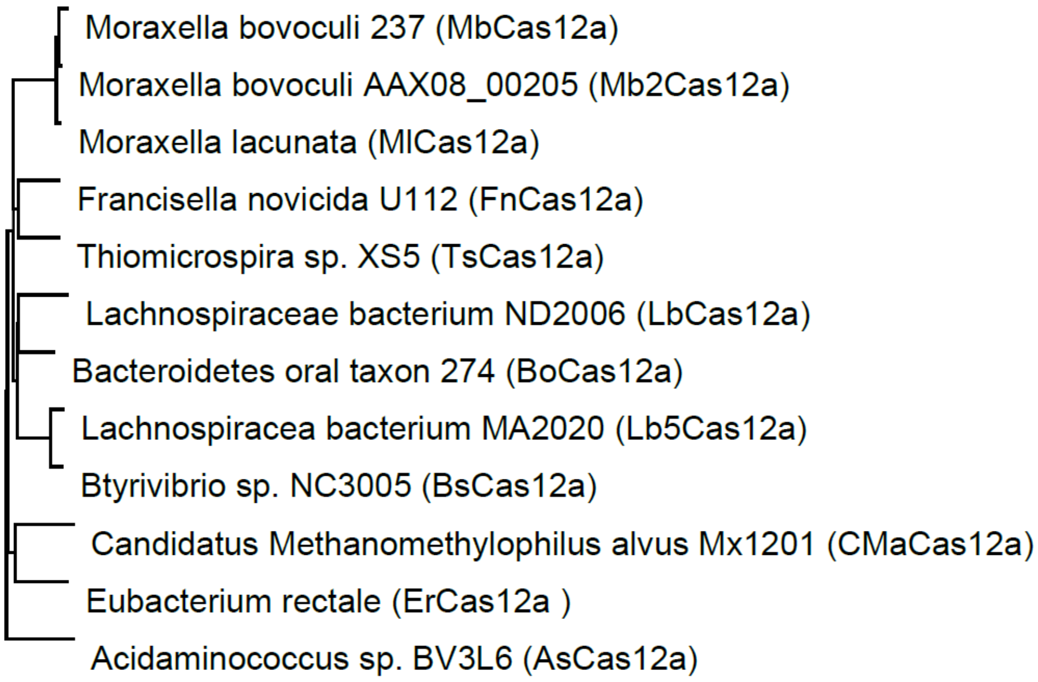

b

AsCas12a	529	EKFKLNFQMPTLASGWDVNKEKNNGAILFVKNGLYYLGIM	568
LbCas12a	519	DKFKLYFQNPQFMGGWDKDKETDYRATILRYGSKYYLAIM	558
FnCas12a	594	EKFKLNFENSTLANGWDKNKEPDNTAILFIKDDKYYLGVM	633
BoCas12a	551	KKIKLNFENSTLMDGWDLNKEPDNTTVIFCKDGLYYLGIM	590
CMaCas12a	502	DKIKVNLKFPTLADGWDLNKERDNKAAILRKDGKYYLAIL	541
MbCas12a	563	EKYKLNFGNPTLLNGWDLNKEKDNFGVILQKDGCYYLALL	602
MlCas12a	550	EKYKLNFGNPTLLNGWDLNKEKDNFGVILQKDGCYYLALL	589
TsCas12a	562	DKFKINFDNNTLLSGWDANKETANASILFKKDGLYYLGIM	601
Lb5Cas12a	499	EKVKLNFNRSTLLNGWDRNKETDNLGVLLLKDGKYYLGIM	538
Lb5Cas12a-RVR	499	EKVKLNFNRSTLLRGWDRNVETDRLGVLLLKDGKYYLGIM	538
BsCas12a	499	EKVKLNFNKSTLLNGWDKNKETDNLGILFFKDGKYYLGIM	538
BsCas12a-RVR	499	EKVKLNFNKSTLLRGWDKNVETDRLGILFFKDGKYYLGIM	538
ErCas12a	516	KKIKLNFGIPTLADGWSKSKEYSNNAIILMRDNLYYLGIF	555
ErCas12a-RVR	516	KKIKLNFGIPTLARGWSKSVEYSRNAIILMRDNLYYLGIF	555
Mb2Cas12a	550	EKYKLNFGNPTLLNGWDLNKEKDNFGVILQKDGCYYLALL	589
Mb2Cas12a-RVR	550	EKYKLNFGNPTLLRGWDLNVEKDRFGVILQKDGCYYLALL	589

Supplementary Fig. 1. Sequence relationship of 12 Cas12a orthologs. a, A phylogenetic tree of 12 Cas12a orthologs based on protein sequence alignment. **b,** protein sequence alignment of Cas12a orthologs and their RVR variants.

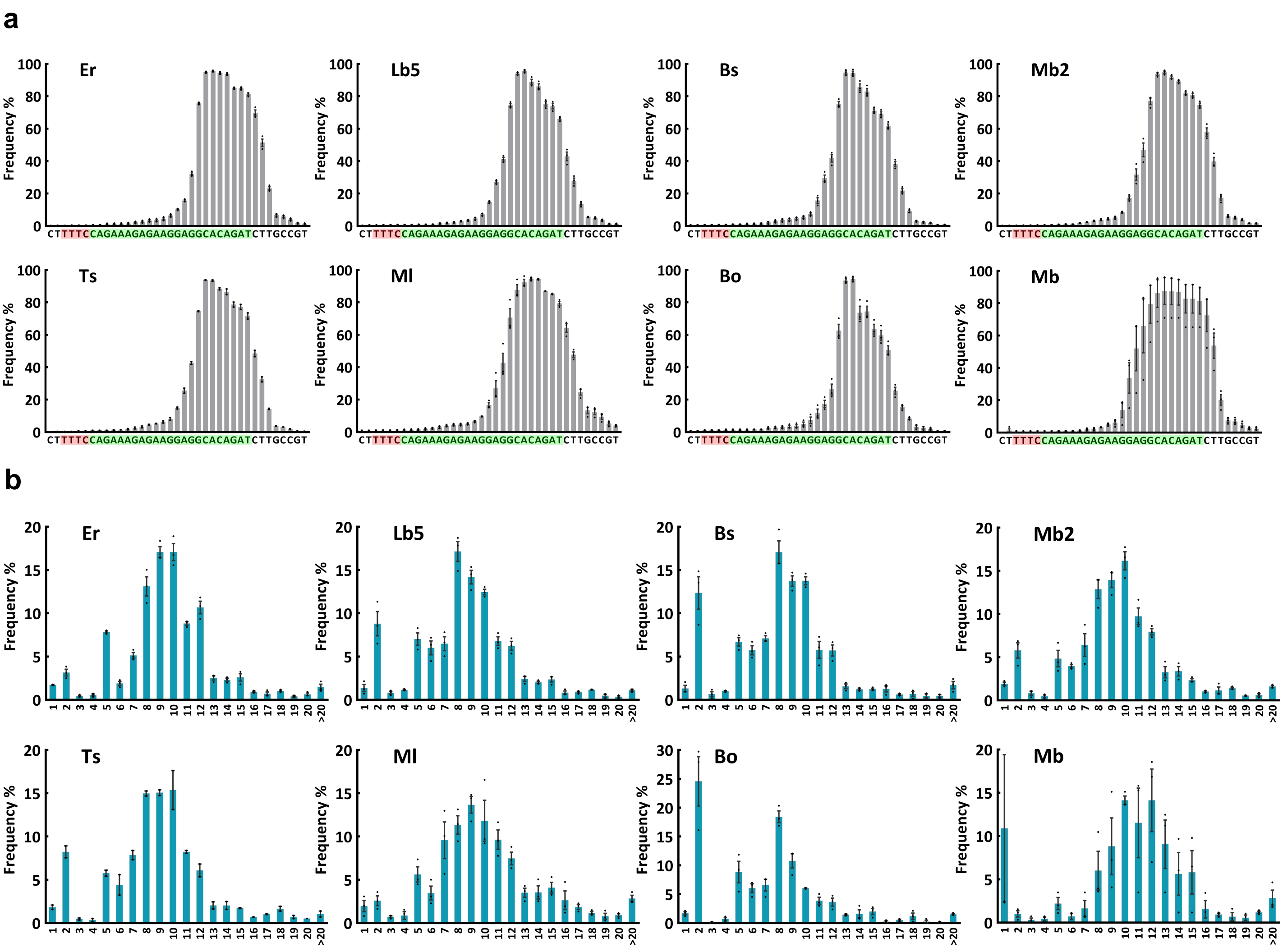

Supplementary Fig. 2. Deletion position and size profiles of eight Cas12a orthologs at the OsDEP1-TTTC site. a, Deletion position. Frequencies (in percentage shown in the y-axis) were calculated using the number of reads with deletions at each nucleotide position divided by the number of all the reads with deletions. PAM sequence is highlighted in red and protospacer sequence is highlighted in green. **b,** Deletion size. Frequencies (in percentage shown in the y-axis) were calculated using the number of reads with N bp deletions divided by the number of all the reads with deletions. Data are presented as mean values ± SEM. n=3 biologically independent samples.

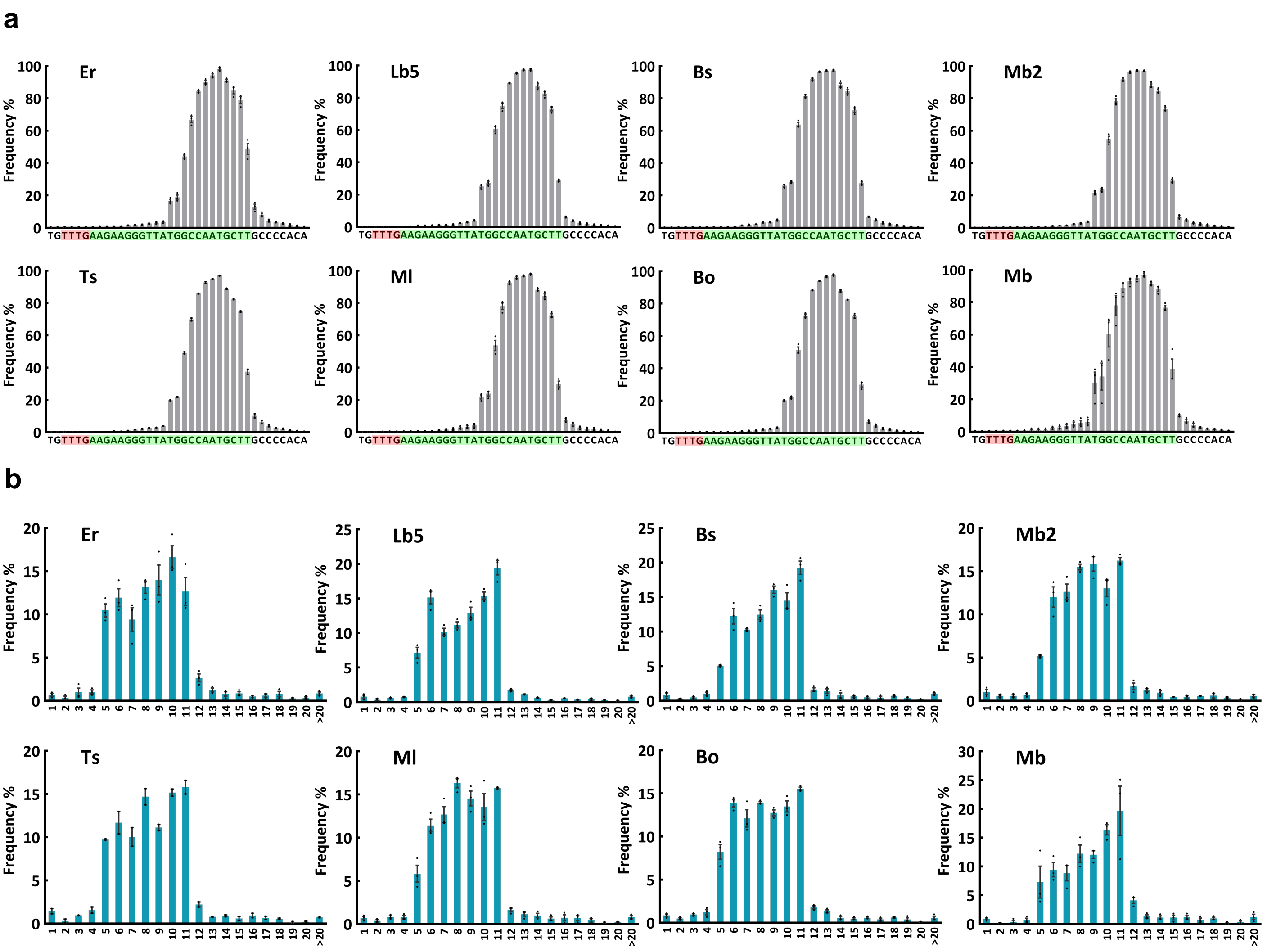

Supplementary Fig. 3. Deletion position and size profiles of eight Cas12a orthologs at the OsEPFL9-TTTG site. a, Deletion position. Frequencies (in percentage shown in the y-axis) were calculated using the number of reads with deletions at each nucleotide position divided by the number of all the reads with deletions. PAM sequence is highlighted in red and protospacer sequence is highlighted in green. **b,** Deletion size. Frequencies (in percentage shown in the y-axis) were calculated using the number of reads with N bp deletions divided by the number of all the reads with deletions. Data are presented as mean values ± SEM. n=3 biologically independent samples.

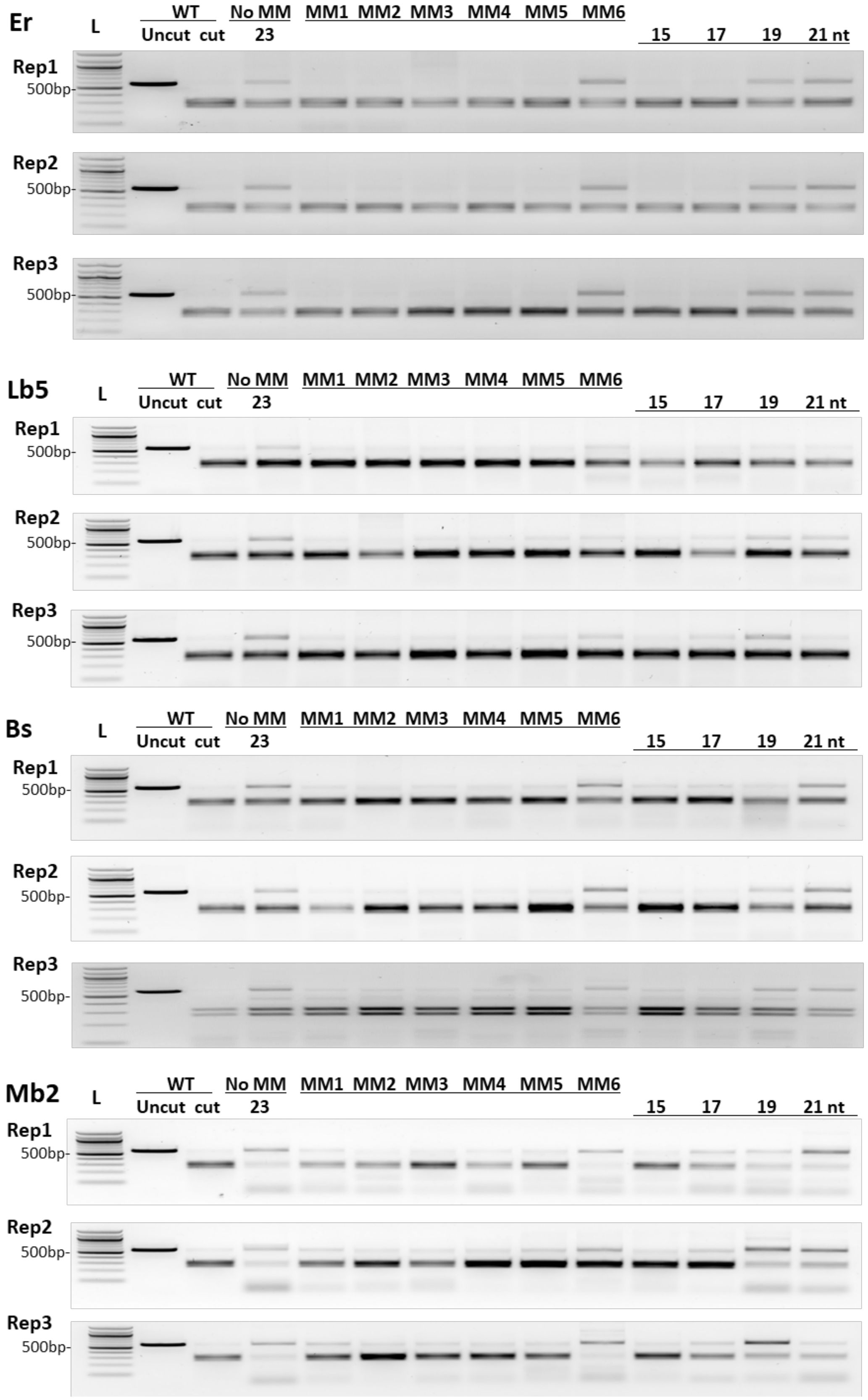

Supplementary Fig. 4. Restriction fragment length polymorphism (RFLP) analysis of specificity and protospacer length requirement of Cas12a orthologs. L, NEB 100 bp DNA Ladder. WT, wild type rice protoplast. Uncut, PCR amplicon of target sites before restriction enzyme digestion. Cut, PCR amplicon of target sites after restriction enzyme digestion. MM, mismatch. Protospacer length (nt) is indicated by numbers. Each experiment includes three biological replicates. Source data are provided as a Source Data file.

OsDEP1-TTTC

```
WT     CCTTTTCCAGAAAGAGAAGGAGGCACAGATCTTGCCGTCTTT
```

LbCas12a

```
Line1  CCTTTTCCAGAAAGAGAAGGAG--------CTTGCCGTCTTT   -8
       CCTTTTCCAGAAAGAGAAGGA--------TCTTGCCGTCTTT   -8
Line5  CCTTTTCCAGAAAGAGAAGGAGG-------CTTGCCGTCTTT   -7
       CCTTTTCCAGAAAGAGAAGGAGG-----------CCGTCTTT   -11
Line6  CCTTTTCCAGAAAGAGAAGGAG---------------TCTTT   -15
       CCTTTTCCAGAAAGAGAAGGAGG-----ATCTTGCCGTCTTT   -5
Line7  CCTTTTCCAGAAAGAGAAGGAGGCACAGATCTTGCCGTCTTT   WT
       CCTTTTCCAGAAAGAGAAGGAGCC------CTTGCCGTCTTT   -6/1
Line10 CCTTTTCCAGAAAGAGAAGGAGGCACAGATCTTGCCGTCTTT   WT
       CCTTTTCCAGAAAGAGAAGG-------------GCCGTCTTT   -13
Line12 CCTTTTCCAGAAAGAGAAGGAGG--------TTGCCGTCTTT   -8
       CCTTTTCCAGAAAGAGAAGGAG---------------TCTTT   -15
Line13 CCTTTTCCAGAAAGAGAAGGAGG-----------CCGTCTTT   -11
       CCTTTTCCAGAAAGAGAAGGA---------CTTGCCGTCTTT   -9
Line16 CCTTTTCCAGAAAGAGAAGGAGGCACA--TCTTGCCGTCTTT   -2
       CCTTTTCCAGAAAGAGAAGGAGA-----ATCTTGCCGTCTTT   -5/1
Line21 CCTTTTCCAGAAAGAGAAGGAGGCA------TTGCCGTCTTT   -6
       CCTTTTCCAGAAAGAGAAGGAG---------TTGCCGTCTTT   -9
Line22 CCTTTTCCAGAAAGAGAA-----------TCTTGCCGTCTTT   -11
       CCTTTTCCAGAAAGAGAAGGAGGCA---ATCTTGCCGTCTTT   -3
Line24 CCTTTTCCAGAAAGAGAAGGAG---------TTGCCGTCTTT   -9
       CCTTTTCCAGAAAGAGAAGGAGG-------CTTGCCGTCTTT   -7
```

ErCas12a

```
Line3  CCTTTTCCAGAAAGAGAAGGAGG-----ATCTTGCCGTCTTT   -5
       CCTTTTCCAGAAAGAGAAGGAGG-----------CCGTCTTT   -11
Line4  CCTTTTCCAGAAAGAGAAGGAG---------TTGCCGTCTTT   -9
       CCTTTTCCAGAAAGAGAAGGAG-----------GCCGTCTTT   -11
Line9  CCTTTTCCAGAAAGAGAAGGAGGCACAGATCTTGCCGTCTTT   WT
       CCTTTTCCAGAAAGAGAAGGAGGCA-------TGCCGTCTTT   -8
Line14 CCTTTTCCAGAAAGAGAAGGAGGCACAGATCTTGCCGTCTTT   WT
       CCTTTTCCAGAAAGAGAAGGAG------------CCGTCTTT   -12
Line16 CCTTTTCCAGAAAGAGAAGGAG------------CCGTCTTT   -12
       CCTTTTCCAGAAAGAGAAGGAG--A---------CCGTCTTT   -11
Line18 CCTTTTCCAGAAAGAGAAGGAG----------------CTTT   -16
       CCTTTTCCAGAAAGAGAAGG-------------GCCGTCTTT   -13
```

Lb5Cas12a

```
Line1  CCTTTTCCAGAAAGAGAAGGA-----------TGCCGTCTTT   -11
       CCTTTTCCAGAAAGAGAAGGA---------CTTGCCGTCTTT   -9
Line5  CCTTTTCCAGAAAGAGAAGGA-------------CCGTCTTT   -13
       CCTTTTCCAGAAAGAGAAGGA------GATCTTGCCGTCTTT   -6
Line6  CCTTTTCCAGAAAGAGAAGGA--------TCTTGCCGTCTTT   -8
       CCTTTTCCAGAAAGAGAAGGAGGCACAGATCTTGCCGTCTTT   WT
```

BsCas12a

```
Line1  CCTTTTCCAGAAAGAGAAGGA-----------TGCCGTCTTT   -11
       CCTTTTCCAGAAAGAGAAGGA---------CTTGCCGTCTTT   -9
Line5  CCTTTTCCAGAAAGAGAAGGAG---------TTGCCGTCTTT   -9
       CCTTTTCCAGAAAGAGAAGGAGG--------TTGCCGTCTTT   -8
Line13 CCTTTTCCAGAAAGAGAAGGAGGCACAGATCTTGCCGTCTTT   WT
       CCTTTTCCAGAAAGAGAAGGA--------TCTTGCCGTCTTT   -8
Line15 CCTTTTCCAGAAAGAGAA------------CTTGCCGTCTTT   -12
       CCTTTTCCAGAAAGAGAAGGAGGCACAGATCTTGCCGTCTTT   WT
Line16 CCTTTTCCAGAAAGAGAAGGAGGCACAGATCTTGCCGTCTTT   WT
       CCTTTTCCAGAAAGAGAAGGAGG-----ATCTTGCCGTCTTT   -5
```

OsDEP1-TTTC

```
WT     CCTTTTCCAGAAAGAGAAGGAGGCACAGATCTTGCCGTCTTT
```

Mb2Cas12a

```
Line4  CCTTTTCCAGAAAGAGAAGGAGGCACAGATCTTGCCGTCTTT   WT
       CCTTTTCCAGAAAGAGAAGGAG----------TGCCGTCTTT   -9
Line5  CCTTTTCCAGAAAGAGAAGGAGG------TCTTGCCGTCTTT   -6
       CCTTTTCCAGAAAGAGAAGGA----------TTGCCGTCTTT   -10
Line6  CCTTTTCCAGAAAGAGAAGGA--------TCTTGCCGTCTTT   -8
       CCTTTTCCAGAAAGAGAAGGAGG-----------CCGTCTTT   -11
Line8  CCTTTTCCAGAAAGAGAAGG----------CTTGCCGTCTTT   -10
       CCTTTTCCAGAAAGAGAAGGA--------------CGTCTTT   -14
Line9  CCTTTTCCAGAAAGAGAAGG--------------CCGTCTTT   -14
       CCTTTTCCAGAAAGAGAAGGAGGCA----TCTTGCCGTCTTT   -4
Line12 CCTTTTCCAGAAAGAGAAGG------------TGCCGTCTTT   -12
       CCTTTTCCAGAAAGAGAAGGAGG-----------CCGTCTTT   -11
Line16 CCTTTTCCAGAAAGAGAAGGAGGCACAGATCTTGCCGTCTTT   WT
       CCTTTTCCAGAAAGAGAAGGAGGCA------TTGCCGTCTTT   -6
```

TsCas12a

```
Line1  CCTTTTCCAGAAAGAGAAGGAGGC---------GCCGTCTTT   -9
       CCTTTTCCAGAAAGAGAAGGAGGCACAGAT--TGCCGTCTTT   -2
Line3  CCTTTTCCAGAAAGAGAAGGAGG--CAGATCTTGCCGTCTTT   -2
       CCTTTTCCAGAAAGAGAAGGAGG-------------------   -27
Line4  CCTTTTCCAGAAAGAG----------------TGCCGTCTTT   -16
       CCTTTTCCAGAAAGAGAAGG------AGATCTTGCCGTCTTT   -6
Line5  CCTTTTCCAGAAAGAGAAGGAGG-------GGTGCCGTCTTT   -7/2
       CCTTTTCCAGAAAGAGAAGGAGG-------GGTGCCGTCTTT   -7/2
Line6  CCTTTTCCAGAA--------------------TGCCGTCTTT   -20
       CCTTTTCCAGAAAGAGAAGGAG----------TGCCGTCTTT   -10
Line7  CCTTTTCCAGAA----------------------CCGTCTTT   -22
       CCTTTTCCAGAACGAGAAGG------------TGCCGTCTTT   -12/1
Line8  CCTTTTCCAGAAAGAGA-------------CTTGCCGTCTTT   -13
       CCTTTTCCAGAAAGAGAAGG-----------TTGCCGTCTTT   -11
Line9  CCTTTTCCAGAAAGAGAAGGAGG-----ATCTTGCCGTCTTT   -5
       CCTTTTCCAGAAAGAGAAGGAGG-----ATCTTGCCGTCTTT   -5
```

BoCas12a

```
Line5  CCTTTTCCAGAAAGAGAAGGAGGCACAGATCTTGCCGTCTTT   WT
       CCTTTTCCAGAAAGAGAAGGAG---------------TCTTT   -14
Line6  CCTTTTCCAGAAAGAGAAGGAG------------------TT   -18
       CCTTTTCCAGAAAGAGAAGGAG---------TTGCCGTCTTT   -9
Line7  CCTTTTCCAGAAAGAGAAGGAG----------TGCCGTCTTT   -10
       CCTTTTCCAGAAAGAGAAGGAGGCACAGATCTTGCCGTCTTT   WT
Line13 CCTTTTCCAGAAAGAGAA------------CTTGCCGTCTTT   -12
       CCTTTTCCAGAAAGAGAAGGAGG--------TTGCCGTCTTT   -8
Line14 CCTTTTCCAGAAAGAGAA------------CTTGCCGTCTTT   -12
       CCTTTTCCAGAAAGAGAAGGAGG--------TTGCCGTCTTT   -8
Line15 CCTTTTCCAGAAAGAGAAGGA--------TCTTGCCGTCTTT   -8
       CCTTTTCCAGAAAGAGAAGGA----CAGATCTTGCCGTCTTT   -4
Line16 CCTTTTCCAGAAAGAGAAGGAGGCACAGATCTTGCCGTCTTT   WT
       CCTTTTCCAGAAAGAGAAGGA---------CTTGCCGTCTTT   -9
```

MbCas12a

```
Line5  CCTTTTCCAGAAAGAGAAGGAGGCACAGATCTTGCCGTCTTT   WT
       CCTTTTCCAGAAAGAGAAGGAG----------TGCCGTCTTT   -10
Line6  CCTTTTCCAGAAAGAGAAGGAGGCACAGATCTTGCCGTCTTT   WT
       CCTTTTCCAGAAAGAGAAGGAG----------TGCCGTCTTT   -10
Line7  CCTTTTCCAGAAAGAGAAGG-------------GCCGTCTTT   -13
       CCTTTTCCAGAAAGAGAAGGA-----------TGCCGTCTTT   -11
Line9  CCTTTTCCAGAAAGAGAAGG--------------CCGTCTTT   -14
       CCTTTTCCAGAAAGAGAAGGAGGC---------GCCGTCTTT   -9
Line11 CCTTTTCCAGAAAGAGAAGGAGGCACAGATCTTGCCGTCTTT   WT
       CCTTTTCCAGAA--------------------TGCCGTCTTT   -20
Line13 CCTTTTCCAGAAAGAGAAGGAGGC--------TGCCGTCTTT   -8
       CCTTTTCCAGAAAGAGAAGGAG------ATCTTGCCGTCTTT   -6
Line15 CCTTTTCCAGAAAGAGAAGG-------GATCTTGCCGTCTTT   -7
       CCTTTTCCAGAAAGAGAAGG-----------------TCTTT   -17
Line17 CCTTTTCCAGAAAGAGAAG------------------TCTTT   -18
       CCTTTTCCAGAAAGAGAAGG------------TGCCGTCTTT   -12
```

Supplementary Fig. 5. Genotypes of T_0 rice plants edited by Cas12a orthologs at the OsDEP1-TTTC site. PAM sequences are in red. Protospacer sequences are in blue. Substituted nucleotides are in green.

```
OsEPFL9-TTTG

WT     ATTTGTTTGAAGAAGGGTTATGGCCAATGCTTGCCCCACATCTA
```

LbCas12a

```
Line1  ATTTGTTTGAAGAAGGGTTATGGCC-----TTGCCCCACATCTA  -5
       ATTTGTTTGAAGAAGGGTTATGGCC--------------ATCTA  -14
Line4  ATTTGTTTGAAGAAGGGTTAT-----------GCCCCACATCTA  -11
       ATTTGTTTGAAGAAGGGTTAT----------TGCCCCACATCTA  -10
Line5  ATTTGTTTGAAGAAGGGTTATGGC-AATGCTTGCCCCACATCTA  -1
       ATTTGTTTGAAGAAGGGTTATGGC----------CCCACATCTA  -10
Line6  ATTTGTTTGAAGAAGGGTTATGGCCA-----TGCCCCACATCTA  -5
       ATTTGTTTGAAGAAGGGTTATGGCC------TGCCCCACATCTA  -6
Line7  ATTTGTTTGAAGAAGGGTTAT------TGCTTGCCCCACATCTA  -6
       ATTTGTTTGAAGAAGGGTTATGGCCA-----TGCCCCACATCTA  -5
Line8  ATTTGTTTGAAGAAGGGTTATGGCC-------GCCCCACATCTA  -7
       ATTTGTTTGAAGAAGGGTTATGGCC-------GCCCCACATCTA  -7
Line10 ATTTGTTTGAAGAAGGGTTATGGC------TTGCCCCACATCTA  -6
       ATTTGTTTGAAGAAGGGTTAT------------CCCCACATCTA  -12
Line11 ATTTGTTTGAAGAAGGGTTATGGCC-----TTGCCCCACATCTA  -5
       ATTTGTTTGAAGAAGGGTTATGGCC-------GCCCCACATCTA  -7
```

ErCas12a

```
Line3  ATTTGTTTGAAGAAGGGTTATGGCCAATGCTTGCCCCACATCTA   WT
       ATTTGTTTGAAGAAGGGTTATG--CA------GCCCCACATCTA  -8
Line7  ATTTGTTTGAAGAAGGGTTATGGCCAATGCTTGCCCCACATCTA   WT
       ATTTGTTTGAAGAAGGGTTATGGC--------GCCCCACATCTA  -8
Line8  ATTTGTTTGAAGAAGGGTTATGGC--------GCCCCACATCTA  -8
       ATTTGTTTGAAGAAGGGTTATGGC--------GCCCCACATCTA  -8
Line11 ATTTGTTTGAAGAAGGGTTATGGCC-----TTGCCCCACATCTA  -5
       ATTTGTTTGAAGAAGGGTTATGGCC----------CCACATCTA  -10
Line12 ATTTGTTTGAAGAAGGGTTATGGC-------TGCCCCACATCTA  -7
       ATTTGTTTGAAGAAGGGTTATGGC--------GCCCCACATCTA  -8
Line14 ATTTGTTTGAAGAAGGGTTATGGCCAATGCTTGCCCCACATCTA   WT
       ATTTGTTTGAAGAAGGGTTATGG---------GCCCCACATCTA  -9
Line15 ATTTGTTTGAAGAAGGGTTATGGCCA------GCCCCACATCTA  -6
       ATTTGTTTGAAGAAGGGTTAT---------------------TA  -21
Line19 ATTTGTTTGAAGAAGGGTTATGG---------GCCCCACATCTA  -9
       ATTTGTTTGAAGAAGGGTTATGGCC-----TTGCCCCACATCTA  -5
Line21 ATTTGTTTGAAGAAGGGTTATGG--------TGCCCCACATCTA  -8
       ATTTGTTTGAAGAAGGGTTATGGCC-------GCCCCACATCTA  -7
Line17 ATTTGTTTGAAGAAGGGTTATGGCCAATGCTTGCCCCACATCTA   WT
       ATTTGTTTGAAGAAGGGTTATGGCC----------CCACATCTA  -10
Line18 ATTTGTTTGAAGAAGGGTTATGGCCAATGCTTGCCCCACATCTA   WT
       ATTTGTTTGAAGAAGGGTTATGGCCAA-----GCCCCACATCTA  -5
Line23 ATTTGTTTGAAGAAGGGTTATGGCC-----TTGCCCCACATCTA  -5
       ATTTGTTTGAAGAAGGGTTATGGC--------GCCCCACATCTA  -8
Line25 ATTTGTTTGAAGAAGGGTTATGGCCAATGCTTGCCCCACATCTA   WT
       ATTTGTTTGAAGAAGGGTTATGGC------TTGCCCCACATCTA  -6
Line26 ATTTGTTTGAAGAAGGGTTATGGCCAA-------CCCACATCTA  -7
       ATTTGTTTGAAGAAGGGTTATGGC------TTGCCCCACATCTA  -6
Line27 ATTTGTTTGAAGAAGGGTTATGGCCAATGCTTGCCCCACATCTA   WT
       ATTTGTTTGAAGAAGGGTTATGGCC-----------CACATCTA  -11
```

Lb5Cas12a

```
Line1  ATTTGTTTGAAGAAGGGTTATGGCCAATGC---------ATCTA  -9
       ATTTGTTTGAAGAAGGGTTATGG---------GCCCCACATCTA  -9
Line2  ATTTGTTTGAAGAAGGGTTATGG---------GCCCCACATCTA  -9
       ATTTGTTTGAAGAAGGGTTATG---------TGCCCCACATCTA  -9
Line3  ATTTGTTTGAAGAAGGGTTATGG---------GCCCCACATCTA  -9
       ATTTGTTTGAAGAAGGGTTATGG--------TGCCCCACATCTA  -8
Line5  ATTTGTTTGAAGAAGGGTTATGGCCAATGCTTGCCCCACATCTA   WT
       ATTTGTTTGAAGAAGGGTTATG---------TGCCCCACATCTA  -9
Line6  ATTTGTTTGAAGAAGGGTTATGGCCAATGCTTGCCCCACATCTA   WT
       ATTTGTTTGAAGAAGGGTTATGGC--------GCCCCACATCTA  -8
Line7  ATTTGTTTGAAGAAGGGTTATGGCCAATGCTTGCCCCACATCTA   WT
       ATTTGTTTGAAGAAGGGTTATGGCC-----TTGCCCCACATCTA  -5
Line18 ATTTGTTTGAAGAAGGGTTATGGC-AATGCTTGCCCCACATCTA  -1
       ATTTGTTTGAAGAAGGGTTATGGC------TTGCCCCACATCTA  -6
Line19 ATTTGTTTGAAGAAGGGTTATGGCCAATGCTTGCCCCACATCTA   WT
       AT--------------------------------CCCACATCTA  -32
```

BsCas12a

```
Line8  ATTTGTTTGAAGAAGGGTTATGGC--------GCCCCACATCTA  -8
       ATTTGTTTGAAGAAGGGTTATGGCC-------GCCCCACATCTA  -7
Line9  ATTTGTTTGAAGAAGGGTTATGGCC------TGCCCCACATCTA  -6
       ATTTGTTTGAAGAAGGGTTATGGCC---------CCCACATCTA  -9
Line10 ATTTGTTTGAAGAAGGGTTATG-----------CCCCACATCTA  -11
       ATTTGTTTGAAGAAGGGTTATG----------GCCCCACATCTA  -10
Line11 ATTTGTTTGAAGAAGGGTTATGGC------TTGCCCCACATCTA  -6
       ATTTGTTTGAAGAAGGGTTATGGC-------TGCCCCACATCTA  -7
Line12 ATTTGTTTGAAGAAGGGTTATGG-------TTGCCCCACATCTA  -7
       ATTTGTTTGAAGAAGGGTTATGGC--ATGCTTGCCCCACATCTA  -2
Line13 ATTTGTTTGAAGAAGGGTTATGG-------TTGCCCCACATCTA  -7
       ATTTGTTTGAAGAAGGGTTATGGC--ATGCTTGCCCCACATCTA  -2
Line14 ATTTGTTTGAAGAAGGGTTATG-----------CCCCACATCTA  -11
       ATTTGTTTGAAGAAGGGTTATGGC-------TGCCCCACATCTA  -7
Line15 ATTTGTTTGAAGAAGGGTTATG-----------CCCCACATCTA  -11
       ATTTGTTTGAAGAAGGGTTATGGC-------TGCCCCACATCTA  -7
Line16 ATTTGTTTGAAGAAGGGTTATGGCCAATGCTTGCCCCACATCTA   WT
       ATTTGTTTGAAGAAGGGTTATG-----------CCCCACATCTA  -11
Line17 ATTTGTTTGAAGAAGGGTTATGGCCAATGCTTGCCCCACATCTA   WT
       ATTTGTTTGAAGAAGGGTTATG-----------CCCCACATCTA  -11
```


```
OsEPFL9-TTTG

WT     ATTTGTTTGAAGAAGGGTTATGGCCAATGCTTGCCCCACATCTA
```


Mb2Cas12a

```
Line1  ATTTGTTTGAAGAAGGGTTATGGCC-----TTGCCCCACATCTA  -5
       ATTTGTTTGAAGAAGGGTTATGGC--------GCCCCACATCTA  -8
Line5  ATTTGTTTGAAGAAGGGTTATGGCC---------CCCACATCTA  -9
       ATTTGTTTGAAGAAGGGTTATGGCC-------GCCCCACATCTA  -7
Line7  ATTTGTTTGAAGAAGGGTTATGGCC---------CCCACATCTA  -9
       ATTTGTTTGAAGAAGGGTTATGGC--------G--CCACATCTA  -10
Line11 ATTTGTTTGAAGAAGGGTTATGGCCA-----TGCCCCACATCTA  -5
       ATTTGTTTGAAGAAGGGTT-----------TTGCCCCACATCTA  -8
Line15 ATTTGTTTGAAGAAGGGTTATGG--------TGCCCCACATCTA  -8
       ATTTGTTTGAAGAAGGGTTATGG--------TGCCCCACATCTA  -8
Line17 ATTTGTTTGAAGAAGGGTTATGG-----GCTTGCCCCACATCTA  -5
       ATTTGTTTGAAGAAGGGTTATG------GCTTGCCCCACATCTA  -6
```

TsCas12a

```
Line1  ATTTGTTTGAAGAAGGGTTATGG--------TGCCCCACATCTA  -8
       ATTTGTTTGAAGAAGGGTTATGG--------TGCCCCACATCTA  -8
Line5  ATTTGTTTGAAGAAGGGTTATGG----------CCCCACATCTA  -10
       ATTTGTTTGAAGAAGGGTTATGGCCAATGCTTGCCCCACATCTA   WT
Line6  ATTTGTTTGAAGAAGGGTTATGGC----------CCCACATCTA  -10
       ATTTGTTTGAAGAAGGGTTATGGC--------GCCCCACATCTA  -8
Line7  ATTTGTTTGAAGAAGGGTTATGGC--------GCCCCACATCTA  -8
       ATTTGTTTGAAGAAGGGTTATGGC--------GCCCCACATCTA  -8
Line8  ATTTGTTTGAAGAAGGGTTATGGC--------GCCCCACATCTA  -8
       ATTTGTTTGAAGAAGGGTTATGGC--------GCCCCACATCTA  -8
Line9  ATTTGTTTGAAGAAGGGTTATGG-------TTGCCCCACATCTA  -7
       ATTTGTTTGAAGAAGGGTTATGGCCAATGCTTGCCCCACATCTA   WT
Line10 ATTTGTTTGAAGAAGGGTTATGGCC----CTTGCCCCACATCTA  -4
       ATTTGTTTGAAGAAGGGTTATGGCC-------GCCCCACATCTA  -7
Line11 ATTTGTTTGAAGAAGGGTTATGG--------TGCCCCACATCTA  -8
       ATTTGTTTGAAGAAGGGTTATGG--------TGCCCCACATCTA  -8
Line12 ATTTGTTTGAAGAAGGGTTATGGCC---GCTTGCCCCACATCTA  -3
       ATTTGTTTGAAGAAGGGTTATGGCCAATGCTTGCCCCACATCTA   WT
Line13 ATTTGTTTGAAGAAGGGTTATGGC------TTGCCCCACATCTA  -6
       ATTTGTTTGAAGAAGGGTTATGGCCAATGCTTGCCCCACATCTA   WT
Line14 ATTTGTTTGAAGAAGGGTTATGGCC----------CCACATCTA  -10
       ATTTGTTTGAAGAAGGGTTATGGCCAATGCTTGCCCCACATCTA   WT
Line16 ATTTGTTTGAAGAAGGGTTAT-------GCTTGCCCCACATCTA  -7
       ATTTGTTTGAAGAAGGGTTATGGCCAATGCTTGCCCCACATCTA   WT
Line17 ATTTGTTTGAAGAAGGGTTATG----------GCCCCACATCTA  -6
       ATTTGTTTGAAGAAGGGTTATGGCCAATGCTTGCCCCACATCTA   WT
Line18 ATTTGTTTGAAGAAGGGTTATG--------TTGCCCCACATCTA  -8
       ATTTGTTTGAAGAAGGGTTATG-------CTTGCCCCACATCTA  -7
```

MlCas12a

```
Line4  ATTTGTTTGAAGAAGGGTTATGGCCAA-----GCCCCACATCTA  -5
       ATTTGTTTGAAGAAGGGTTATGGCCAATGCTTGCCCCACATCTA   WT
Line7  ATTTGTTTGAAGAAGGGT---------------CCCCACATCTA  -15
       ATTTGTTTGAAGAAGGGTTATGGCCAATGCTTGCCCCACATCTA   WT
Line8  ATTTGTTTGAAGAAGGGT---------------CCCCACATCTA  -15
       ATTTGTTTGAAGAAGGGTTATGG--------TGCCCCACATCTA  -8
Line9  ATTTGTTTGAAGAAGGGT---------------CCCCACATCTA  -15
       ATTTGTTTGAAGAAGGGTTATGGCCAATGCTTGCCCCACATCTA   WT
Line13 ATTTGTTTGAAGAAGGGTTATGGC------TTGCCCCACATCTA  -6
       ATTTGTTTGAAGAAGGGTTATGGCCAATGCTTGCCCCACATCTA   WT
Line14 ATTTGTTTGAAGAAGGGTTATGGCC----------CCACATCTA  -10
       ATTTGTTTGAAGAAGGGTTATGGCCAATGCTTGCCCCACATCTA   WT
```

BoCas12a

```
Line11 ATTTGTTTGAAGAAGGGTTATGG---------GCCCCACATCTA  -9
       ----------47 bp deletion----------------TCTA  -47
Line12 ATTTGTTTGAAGAAGGGTTATGGCCAATGCTTGCCCCACATCTA   WT
       ATTTGTTTGAAGAAGGGTTATGGC--------GCCCCACATCTA  -8
```

MbCas12a

```
Line1  ATTTGTTTGAAGAAGGGTTATGGC--------GCCCCACATCTA  -8
       ATTTGTTTGAAGAAGGGTTATGGCCAATGCTTGCCCCACATCTA   WT
Line9  ATTTGTTTGAAGAAGGGTTATGGCC---------CCCACATCTA  -9
       ATTTGTTTGAAGAAGGGTTATGGCCAATGCTTGCCCCACATCTA   WT
Line11 ATTTGTTTGAAGAAGGGTTATGGCC---------CCCACATCTA  -9
       ATTTGTTTGAAGAAGGGTTATG-----TGCTTGCCCCACATCTA  -5
Line12 ATTTGTTTGAA-----------35 bp deletion--------  -35
       ATTTGTTTGAAGAAGGGTTATGGCCAATGCTTGCCCCACATCTA   WT
Line13 ATTTGTTTGAAGAAGGGTTAT---------TTGCCCCACATCTA  -9
       ATTTGTTTGAAGAAGGGTTATGGCC--------CCCCACATCTA  -8
Line14 ATTTGTTTGAAGAAGGGTTAT----------TGCCCCACATCTA  -10
       ATTTGTTTGAAGAAGGGTTATGGCCAATGCTTGCCCCACATCTA   WT
Line15 ATTTGTTTGAAGAAGGGTTAT------TGCTTGCCCCACATCTA  -6
       ATTTGTTTGAAGAAGGGTTATGGCCAATGCTTGCCCCACATCTA   WT
Line27 ATTTGTTTGAAGAAGGGTTATGG---------GCCCCACATCTA  -9
       ATTTGTTTGAAGAAGGGTTATGGCC------TGCCCCACATCTA  -6
Line29 ATTTGTTTGAAGAAGGGTT-----------TTGCCCCACATCTA  -11
       ATTTGTTTGAAGAAGGGTTATGG---------GCCCCACATCTA  -9
```

Supplementary Fig. 6. Genotypes of T_0 rice plants edited by Cas12a orthologs at the OsEPFL9-TTTG site. PAM sequences are in red. Protospacer sequences are in blue.

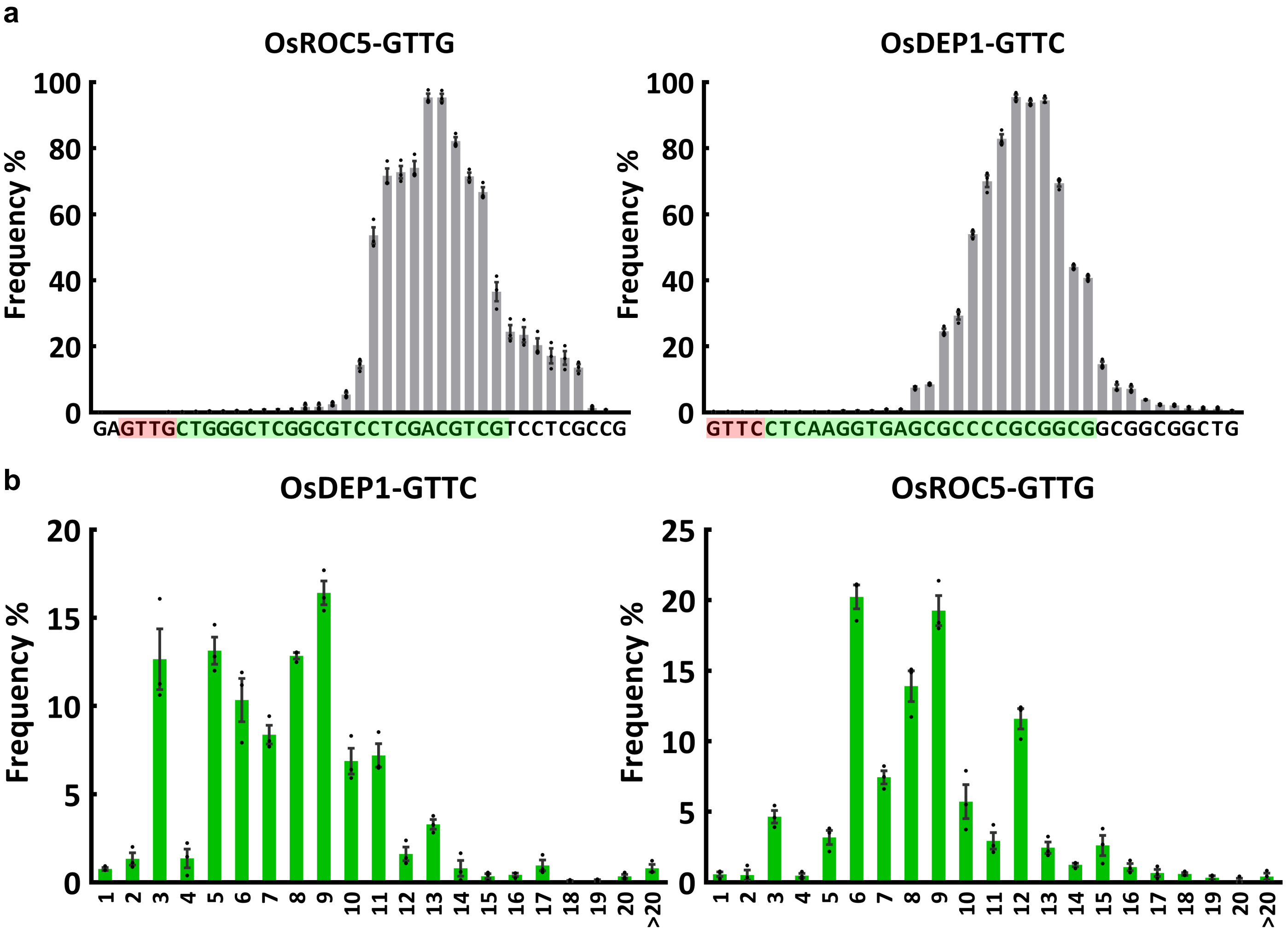

Supplementary Fig. 7. Deletion position and size profiles of Mb2Cas12a at OsROC5-GTTG and OsDEP1-GTTC sites. a, Deletion position. Frequencies (in percentage shown in the y-axis) were calculated using the number of reads with deletions at each nucleotide position divided by the number of all the reads with deletions. PAM sequence is highlighted in red and protospacer sequence is highlighted in green. **b,** Deletion size. Frequencies (in percentage shown in the y-axis) were calculated using the number of reads with N bp deletions divided by the number of all the reads with deletions. Data are presented as mean values ± SEM. n=3 biologically independent samples.

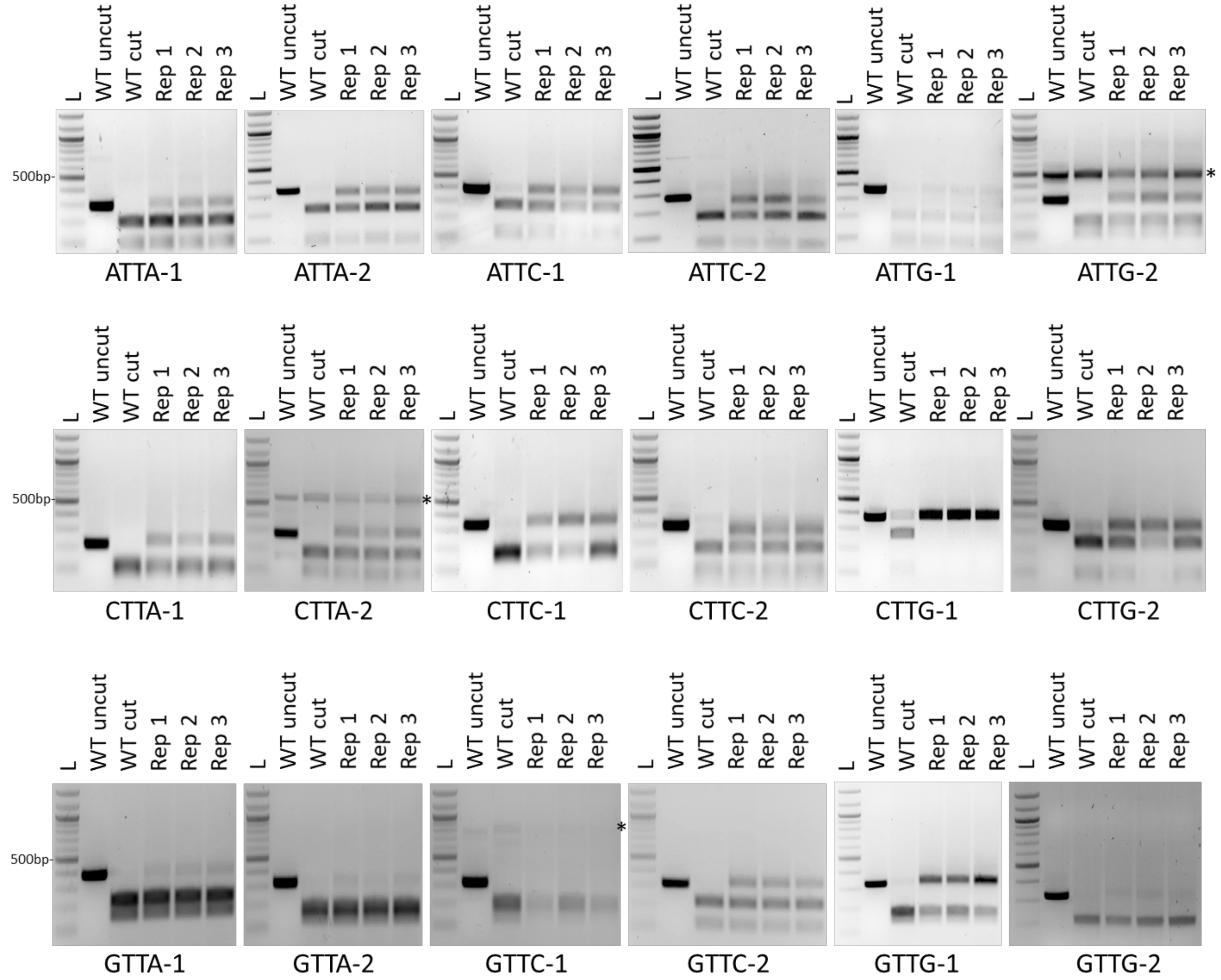

Supplementary Fig. 8. Restriction fragment length polymorphism (RFLP) analysis of genome editing at VTTV PAMs using Mb2Cas12a. L, NEB 100 bp DNA Ladder. WT, wild type rice protoplast. Uncut, PCR amplicon of target sites before restriction enzyme digestion. Cut, PCR amplicon of target sites after restriction enzyme digestion. Asterisks indicate non-specific amplifications. Each experiment includes three biological replicates. Source data are provided as a Source Data file.

```
Os09g37860-CTTC

   WT   ATTCGCTCCTTCTCCTGAGGAGCAAGAGCCATCACAGGCAAGAAA

Line1   ATTCGCTCCTTCTCCTGAGGAGCAAGAGCCATCACAGGCAAGAAA   WT
        ATTCGCTCCTTCTCCTGAGGAGCAAG-----------GCAAGAAA  -11
Line2   ATTCGCTCCTTCTCCTGAGGAGCAAGAGCCATCACAGGCAAGAAA   WT
        ATTCGCTCCTTCTCCTGAGGAGCAAG---CATCACAGGCAAGAAA  -3
Line3   ATTCGCTCCTTCTCCTGAGGAGCAAGAGCCATCACAGGCAAGAAA   WT
        ATTCGCTCCTTCTCCTGAGGAGCAAG-----------GCAAGAAA  -11
Line4   ATTCGCTCCTTCTCCTGAGGAGCAAGAGCCATCACAGGCAAGAAA   WT
        ATTCGCTCCTTCTCCTG---------------------CAAGAAA  -21
Line5   ATTCGCTCCTTCTCCTGAGGAGCAAGAGCCATCACAGGCAAGAAA   WT
        -----------37 bp deletion----CATCACAGGCAAGAAA  -37
Line8   ATTCGCTCCTTCTCCTGAGGAGCAAGAGCCATCACAGGCAAGAAA   WT
        ATTCGCTCCTTCTCCTGAGGAGCAA---------CAGGCAAGAAA  -9
        ATTCGCTCCTTCTCCTGAGGAGCA--AGCCATCACAGGCAAGAAA  -2
Line9   ATTCGCTCCTTCTCCTGAGGAGCAAGAGCCATCACAGGCAAGAAA   WT
        ATTCGCTCCTTCTCCTGAGGAGCAAG---CATCACAGGCAAGAAA  -3
Line10  ATTCGCTCCTTCTCCTGAGGAGCAAGAGCCATCACAGGCAAGAAA   WT
        ATTCGCTCCTTCTCCTGAGGAGCAAGA--------AGGCAAGAAA  -8
Line12  ATTCGCTCCTTCTCCTGAGGAGCAAGAGCCATCACAGGCAAGAAA   WT
        ATTCGCTCCTTCTCCTGAGGAGCAAG-G------CAGGCAAGAAA  -7
Line13  ATTCGCTCCTTCTCCTGAGGAGCAAGAGCCATCACAGGCAAGAAA   WT
        ATTCGCTCCTTCTCCTGAGGAGCAAGA-------CAGGCAAGAAA  -7
Line14  ATTCGCTCCTTCTCCTGAGGAGCAAGAGCCATCACAGGCAAGAAA   WT
        ATTCGCTCCTTCTCCTGAGGAGCAAGA--------AGGCAAGAAA  -8
Line15  ATTCGCTCCTTCTCCTGAGGAGCAAGAGCCATCACAGGCAAGAAA   WT
        ATTCGCTCCTTCTCCTGAGGAGCAAGA--------AGGCAAGAAA  -8
Line16  ATTCGCTCCTTCTCCTGAGGAGCAAGAGCCATCACAGGCAAGAAA   WT
        ATTCGCTCCTTCTCCTGAGGAGCAAGA--------AGGCAAGAAA  -8
Line17  ATTCGCTCCTTCTCCTGAGGAGCAAGAGCCATCACAGGCAAGAAA   WT
        ATTCGCTCCTTCTCCTGAGGAGCAAGA-------CAGGCAAGAAA  -7
Line18  ATTCGCTCCTTCTCCTGAGGAGCAAGAGCCATCACAGGCAAGAAA   WT
        ATTGCCATAAGTATATGAG---------------------AGAAA  -21/11
Line19  ATTCGCTCCTTCTCCTGAGGAGCAAGAGCCATCACAGGCAAGAAA   WT
        ATTGCCATAAGTATATGAG---------------------AGAAA  -21/11
Line20  ATTCGCTCCTTCTCCTGAGGAGCAAGAGCCATCACAGGCAAGAAA   WT
        ATTGCCATAAGTATATGAG---------------------AGAAA  -21/11
Line22  ATTCGCTCCTTCTCCTGAGGAGCAAGAGCCATCACAGGCAAGAAA   WT
        ATTCGCTCCTTCTCCTGAGGAGCAAGA--------AGGCAAGAAA  -8
Line24  ATTCGCTCCTTCTCCTGAGGAGCAAGAGCCATCACAGGCAAGAAA   WT
        ATTCGCTCCTTCTCCTGAGGAGCAAG-----------GCAAGAAA  -11
Line25  ATTCGCTCCTTCTCCTGAGGAGCAAGAGC----ACAGGCAAGAAA  -4
        ATTCGCTCCTTCTCCTGAGGAGCAAGAGCCATCACAG----GAAA  -4
```

```
Os12g24050-GTTG

   WT   CTGAACGTTGAGCATATGGTTGTAACTTCAGAAGTACCAAATCAC

Line3   CTGAACGTTGAGCATATGGTTGTAACTTCAGAAGTACCAAATCAC   WT
        CTGAACGTTGAGCATATGGT---------------ACCAAATCAC  -15
Line5   CTGAACGTTGAGCATATGGTTGTA--------AGTACCAAATCAC  -8
        CTGAACGTTGAGCATATGGTTGTA----------TACCAAATCAC  -10
Line19  CTGAACGTTGAGCATATGGTTGTAACTTCAGAAGTACCAAATCAC   WT
        CTGAACGTTGAGCATATGGTTGTAA--------GTACCAAATCAC  -8
Line21  CTGAACGTTGAGCATATGGTTGTAACTTCAGAAGTACCAAATCAC   WT
        CTGAACGTTGAGCATATGGTTGTA------------CCAAATCAC  -12
Line22  CTGAACGTTGAGCATATGGTTGTAACTTCAGAAGTACCAAATCAC   WT
        CTGAACGTTGAGCATATGGTTGTA------------CCAAATCAC  -12
Line24  CTGAACGTTGAGCATATGGTTGTAACTTCAGAAGTACCAAATCAC   WT
        CTGAACGTTGAGCATATGGT---------------ACCAAATCAC  -15
```

Supplementary Fig. 9. Genotypes of T_0 rice plants edited by Mb2Cas12a at two VTTV sites. PAM sequences are in red. Protospacer sequences are in blue. Substituted nucleotides are in green.

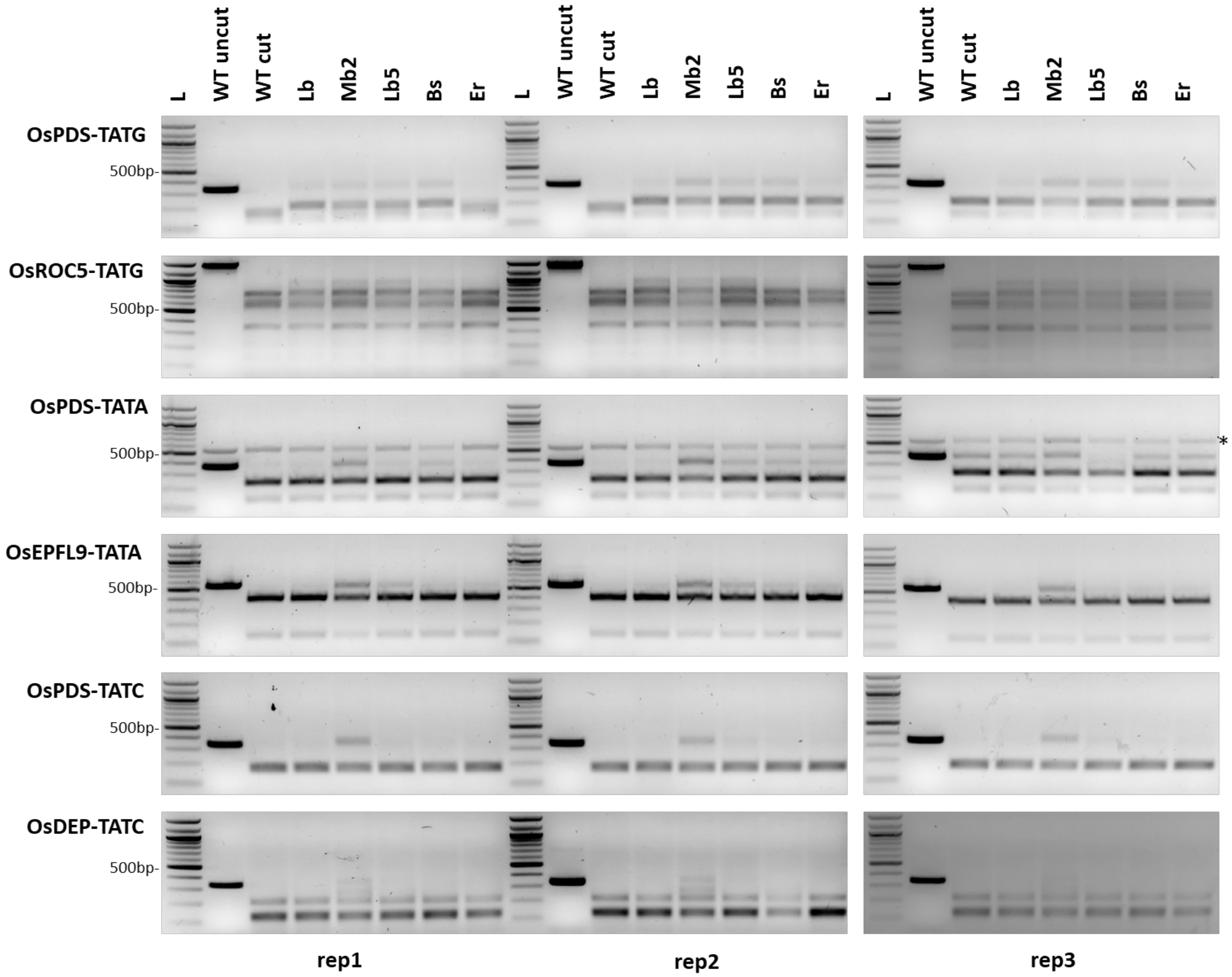

Supplementary Fig. 10. Restriction fragment length polymorphism (RFLP) analysis of genome editing using RVR variants of Cas12a orthologs in rice protoplasts. L, NEB 100 bp DNA Ladder. WT, wild type rice protoplast. Uncut, PCR amplicon of target sites before restriction enzyme digestion. Cut, PCR amplicon of target sites after restriction enzyme digestion. The asterisk indicates non-specific amplifications. Each experiment includes three biological replicates. Source data are provided as a Source Data file.

```
Mb2Cas12a       550   EKYKLNFGNPTLLNGWDLNKEKDNFGVILQKDGCYYLALL   589
Mb2Cas12a-RVR   550   EKYKLNFGNPTLLRGWDLNVEKDRFGVILQKDGCYYLALL   589
Mb2Cas12a-RVRR  550   EKYKLNFGNPTLLRGWDLNVEKDRFGVILQKDGCYYLALL   589
                                   N563R  K569V N573R

Mb2Cas12a       590   DKAHKKVFDNAPNTGKNVYQKMVYKLLPGPNKMLPKVFFA   629
Mb2Cas12a-RVR   590   DKAHKKVFDNAPNTGKNVYQKMVYKLLPGPNKMLPKVFFA   629
Mb2Cas12a-RVRR  590   DKAHKKVFDNAPNTGKNVYQKMVYKLLPGPNKMLPRVFFA   629
                                                         K625R
```

Supplementary Fig. 11. Sequence alignment showing the mutated amino acid residues in Mb2Cas12a-RVR and Mb2Cas12a-RVRR variants.

a

```
Mb2Cas12a      161   EKFSTYFTGFHDNRKNMYSD   180
Mb2Cas12a-v1   161   EKFSTYFTGFHRNRKNMYSD   180
Mb2Cas12a-v2   161   EKFSTYFTGFHRNRKNMYSD   180
                                D172R

Mb2Cas12a      561   LLNGWDLNKEKDNFGVILQK   580
Mb2Cas12a-v1   561   LLNGWDLNKEKDNFGVILQK   580
Mb2Cas12a-v2   561   LLRGWDLNREKDNFGVILQK   580
                       N563R   K569R
```

b

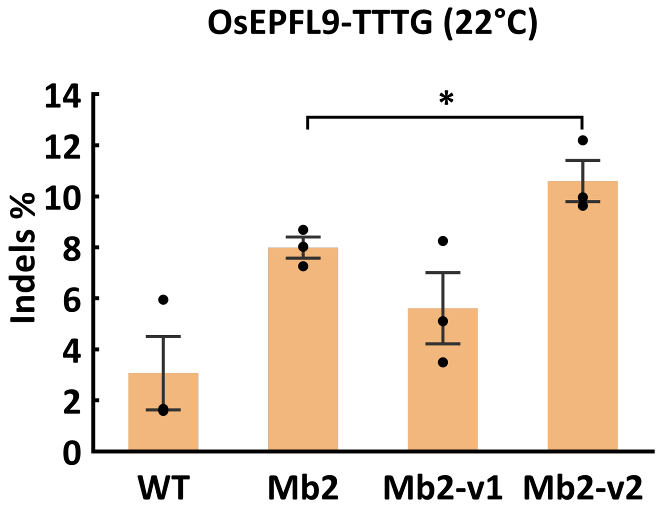

Supplementary Fig. 12. Genome editing using Mb2Cas12a variants in rice protoplasts. a, Sequence alignment showing the mutated amino acid residues in Mb2Cas12a-v1 and Mb2Cas12a-v2 compared to the Mb2Cas12a. **b**, Insertion and deletion (Indel) frequencies of MbCas12a and its variants at the OsEPFL9-TTTG site in rice protoplast. Indel frequencies were measured by next generation sequencing of PCR amplicons. Data are presented as mean values ± SEM. n=3 biologically independent samples. The asterisk indicates significant differences (p=0.045013<0.05) between two treatments using two-sided Student's t-test.

OsDEP1-crRNA01

```
WT     CATGTCTTTGCTACTGTTGCAAGTGCTCACCCAAGTGCAAAAG

B-1    CATGTCTTTGCTACTGTTGCAAGTG------CAAGTGCAAAAG -6
       CATGTCTTTGCTACTGTTGCAAGTGCTCACCCAAGTGCAAAAG  WT
B-2    CATGTCTTTGCTACTGTTG------------CAAGTGCAAAAG -12
       CATGTCTTTGCTACTGTTGCAAGTGCTCACCCAAGTGCAAAAG  WT
B-3    CATGTCTTTGCTACTGTTGCA-----------AAGTGCAAAAG -11
       CATGTCTTTGCTACTGTTGCAAGTGCTCACCCAAGTGCAAAAG  WT

D-1    -------------------------------CAAGTGCAAAAG -36
       CATGTCTTTGCTACTGTTGCAA----------AAGTGCAAAAG -10
D-2    CATGTCTTTGCTACTGTTG------------CAAGTGCAAAAG -12
       CATGTCTTTGCTACTGTTGCAAGT----ACCCAAGTGCAAAAG -4

F-2    CATGTCTTTGCTACTGTTGCAAGT---------AGTGCAAAAG -9
       CATGTCTTTGCTACTGTTGCAAGTG--CACCCAAGTGCAAAAG -2

G-1    ---------------------------------AGTGCAAAAG -512/40
       ------------------------------CCAAGTGCAAAAG -501/46
G-2    --------------------------------AAGTGCAAAAG -505
       -------------------------------CAAGTGCAAAAG -502/1
G-3    -------------------------------------CAAAAG -510
       -------------------------------------CAAAAG -508

I-1    CATGTCTTTGCTAC-------------------AGTGCAAAAG -19
       CATGTCTTTGCTACTGT------------------AGCAAAAG -18
I-2    CATGTCTTTGCTACTGTTGCAA-------------TGCAAAAG -13
       CATGTCTTTGCTACTGTTGCAAGTGCTCACCCAAGTGCAAAAG  WT

J-2    CATGTCTTTGCTACTGT------------------TGCAAAAG -18
       CATGTCTTTGCTACTGTTGCAAGTGCTCACCCAAGTGCAAAAG  WT
```

OsDEP1-crRNA02

```
WT     TTTCCTTTTCCAGAAAGAGAAGGAGGCACAGATCTTGCCGTCT

A-1    TTTCCTTTTCCAGAAAGAGAAGGA-----------TGCCGTCT -11
       TTTCCTTTTCCAGAAAGAGAAGGAG--------CTTGCCGTCT -8
A-3    TTTCCTTTTCCAGAAAGAG------------------------ -33
       TTTCCTTTTCCAGAAAGAGAAGGAGGCACAGATCTTGCCGTCT  WT

B-1    TTTCCTTTTCCAGAAAGAGAAGGAGGC------CTTGCCGTCT -6
       TTTCCTTTTCCAGAAAGAGAAGGAGGCACAGATCTTGCCGTCT  WT
B-2    TTTCCTTTTCCAGAAAGAGAAGGAG--------CTTGCCGTCT -8
       TTTCCTTTTCCAGAAAGAGAAGGAGGCACAGATCTTGCCGTCT  WT
B-3    TTTCCTTTTCCAGAAAGAGAAGGA----------TTGCCGTCT -10
       TTTCCTTTTCCAGAAAGAGAAGGAG---------TTGCCGTCT -9

D-1    TTTCCTTTTCCAGAAAGAGAAGGAG-----------GCCGTCT -11
       TTTCCTTTTCCAGAAAGAGAAGGAG-----GATCTTGCCGTCT -5
D-2    TTTCCTTTTCCAGAAAGAGAAGGA-----------TGCCGTCT -11
       TTTCCTTTTCCAGAAAGAGAAGGAGGCACAG--CTTGCCGTCT -2

G-1    TTTCCTTTTCCAGAAAG-------------------------- -512/40
       TTTCCTTTTCCAGAAAGAGAAGGAG------------------ -501/46
G-2    TTTCCTTTTCCAGAAAGAGAAG--------------------- -505
       TTTCCTTTTCCAGAAAGAGAAGGAGG----------------- -502/1
G-3    TTTCCTTTTCCAGAAAGAGAAGG-------------------- -510
       TTTCCTTTTCCAGAAAGAGAAGGAG------------------ -508

H-1    TTTCCTTTTCCAGAAAGAGAAGGAGGCAC--ATCTTGCCGTCT -2
       TTTCCTTTTCCAGAAAGAGAAGGAGGCACAGATCTTGCCGTCT  WT
H-2    TTTCC-------------------------------------- -38
       TTTCCTTTTCCAGAAAGAGAAGGAGGC--------TGCCGTCT -8

I-1    TTTCCTTTTCCAGAAAGAGAAGGAG--------CTTGCCGTCT -8
       TTTCCTTTTCCAGAAAGAGAAG---ATTTTGACATTGCCGTCT -12/9
I-2    TTTCCTTTTCCAGAAAGAGAAGGAG----------TGCCGTCT -10
       TTTCCTTTTCCAGAAAGAGAAGGAG-----GATCTTGCCGTCT -5
I-3    TTTCCTTTTCCAGAAAGAGAAGGAG------------------ -23
       TTTCCTTTTCCAGAAAGAGAAGGAG-------TCTTGCCGTCT -7
```

OsROC5-crRNA01

```
WT     GCGCCATTTCTGCTTCCTGCAATGCCGGTAGACACCTCCTCAA

A-2    GCGCCATTTCTGCTTCCTGCAATGC-----GACACCTCCTCAA -5
       GCGCCATTTCTGCTTCCTGCAATGCCGGTAGACACCTCCTCAA  WT
A-3    GCGCCATTTCTGCTTCCTG-------------CACCTCCTCAA -13
       GCGCCATTTCTGCTTCCTGCAATGCCGGTAGACACCTCCTCAA  WT

B-1    GCGCCATTTCTGCTTCCTGCAATG-----------CTCCTCAA -11
       GCGCCATTTCTGCTTCCTGCAAT--------ACACCTCCTCAA -8
B-2    GCGCCATTTCTGCTTCCTGCAATG------------TCCTCAA -12
       GCGCCATTTCTGCTTCCTGCAATGCCG-------CCTCCTCAA -7
B-3    GCGCCATTTCTGCTTCCTGCAAT---------CACCTCCTCAA -9
       GCGCCATTTCTGCTTCCTGCAATG-------ACACCTCCTCAA -7

D-1    GCGCCATTTCTGCTTCCTG-------------CACCTCCTCAA -13
       GCGCCATTTCTGCTTCCTGCAATGCCGGTAGACACCTCCTCAA  WT
D-2    GCGC---------------------------ACACCTCCTCAA -27
       GCGCCATTTCTGCTTCCTGCAATG-------ACACCTCCTCAA -7

G-1    GCGCCATTTCTGCTTCCTGCAAT-------GACACCTCCTCAA -7
       GCGCCATTTCTGCTTCCTGCAATGCCGGTAGACACCTCCTCAA  WT

H-2    GCGCCATTTCTGCTTCCTGCAAT----------ACCTCCTCAA -10
       GCGCCATTTCTGCTTCCTGCAATGCCGGTAGACACCTCCTCAA  WT

I-1    GCGCCATTTCTGCTTCCTGCAAT-------GACACCTCCTCAA -7
       GCGCCATTTCTGCTTCCTGCAATGCCGGTAGACACCTCCTCAA  WT
I-2    GCGCCATTTCTGCTTCCTGCAA-------------CTCCTCAA -13
       GCGCCATTTCTGCTTCCTGCAATG---------ACCTCCTCAA -9
I-3    GCGCCATTTCTGCTTCCTGCAAT-----------CCTCCTCAA -11
       GCGCCATTTCTGCTTCCTGCAATGCCGGTAGACACCTCCTCAA  WT
```

OsROC5-crRNA02

```
WT     TCCGGTTTTGTAAGCAGCTGGCTGAGGGTGCATGGGCAGTAGT

A-1    TCCGGTTTTGTAAGCAGCT------------------AGTAGT -18
       TCCGGTTTTGTAAGCAGCT------------------AGTAGT -18
A-2    TCCGGTTTTGTAAGCAGCTGGCTG-----------GCAGTAGT -11
       TCCGGTTTTGTAAGCAGCTGGCTG-------ATGGGCAGTAGT -7
A-3    TCCGGTTTTGTAAGCAGCTGGCTG----------GGCAGTAGT -10
       TCCGGTTTTGTAAGCAGCTGGCTGA--------GGGCAGTAGT -8

B-1    TCCGGTTTTGTAAGCAGCTGGCTGA------ATGGGCAGTAGT -6
       TCCGGTTTTGTAAGCAGCTGGCT----GTGCATGGGCAGTAGT -4
B-2    TCCGGTTTTGTAAGCAGCTGGCTG-----------GCAGTAGT -11
       TCCGGTTTTGTAAGCAGCTGGCTG----------GGCAGTAGT -10
B-3    TCCGGTTTTGTAAGCAGCTGGCTG----------GGCAGTAGT -10
       TCCGGTTTTGTAAGCAGCTGGCTG--------TGGGCAGTAGT -8

D-1    TCCGGTTTTGTAAGCAGCTGGCTG--------TGGGCAGTAGT -8
       TCCGGTTTTGTAAGCAGCTGGCTGA------ATGGGCAGTAGT -6
D-2    TCCGGTTTTGTAAGCAGCTGGCTG----------GGCAGTAGT -10
       TCCGGTTTTGTAAGCAGCTGGCTGAGGG---ATGGGCAGTAGT -3

E-3    TCCGGTTTTGTAAGCAGCTGGCT---------TGGGCAGTAGT -9
       TCCGGTTTTGTAAGCAGCTGGCT--GGGTGCATGGGCAGTAGT -2

F-1    TCCGGTTTTGTAAGCAGCTGGCTGA---------GGCAGTAGT -9
       TCCGGTTTTGTAAGCAGCTGGCTG------CATGGGCAGTAGT -6

G-1    TCCGGTTTTGTAAGCAGCTGGCT-----------------AGT -17
       TCCGGTTTTGTAAGCAGCTGGCT----------GGGCAGTAGT -10
G-2    TCCGGTTTTGTAAG----------------------------- -34
       TCCGGTTTTGTAAGCAGCTG---------GCATGGGCAGTAGT -9
G-3    TCCGGTTTTGTAAGCAGCTGGCTGAGGG--------CAGTAGT -8
       TCCGGTTTTGTAAGCAGCTGGCTGAGGGTGCATGGGCAGTAGT  WT

H-1    TCCGGTTTTGTAAGCAGCTGGCTGAGG---------CAGTAGT -9
       TCCGGTTTTGTAAGCAGCTGGCTGAGGGTGCATGGGCAGTAGT  WT
H-2    TCCGGTTTTGTAAGCAGCTGGCTGA-------TGGGCAGTAGT -7
       TCCGGTTTTGTAAGCAGCTGGCTGA-------TGGGCAGTAGT -7

I-1    TCCGGTTTTGTAAGCAGCTGGCTG----------GGCAGTAGT -10
       TCCGGTTTTGTAAGCAGCTGGCTGAGGGTGCATGGGCAGTAGT  WT
I-2    TCCGGTTTTGTAAGCAGCTGGCTG-----------GCAGTAGT -11
       TCCGGTTTTGTAAGCAGCTGGCTG-----------GCAGTAGT -11
I-3    TCCGGTTTTGTAAGCAGCTGGCTG----------GGCAGTAGT -10
       TCCGGTTTTGTAAGCAGCTGGCTGA------ATGGGCAGTAGT -6
```

Supplementary Fig. 13. Genotypes of T_0 rice plants for the ten multiplexing systems. PAM sequences are in red. Protospacer sequences are in blue. Substituted nucleotides are in green.

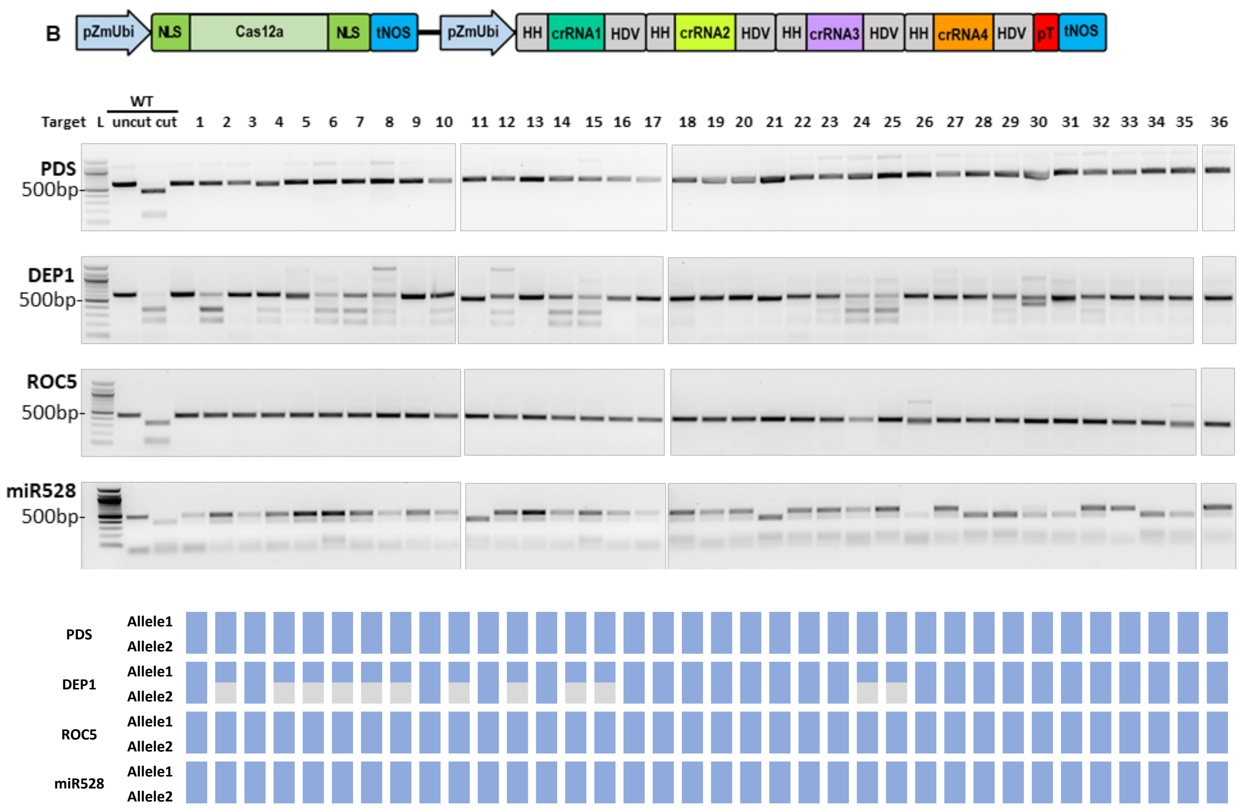

Supplementary Fig. 14. Analysis of multiplexing system B at four target genes with 36 T_0 lines. An illustration of the multiplexing strategy is shown in the upper panel. The middle panel shows RFLP based genotyping data at four target sites among independent lines. L, NEB 100 bp DNA Ladder. WT, wild type rice plants. Uncut, PCR amplicon of target sites before restriction enzyme digestion. Cut, PCR amplicon of target sites after restriction enzyme digestion. The lower panel is a summary table showing editing (blue) or non-editing (gray) of both alleles based on RFLP and Sanger sequencing. Rice stable transformation was not repeated. Source data are provided as a Source Data file.

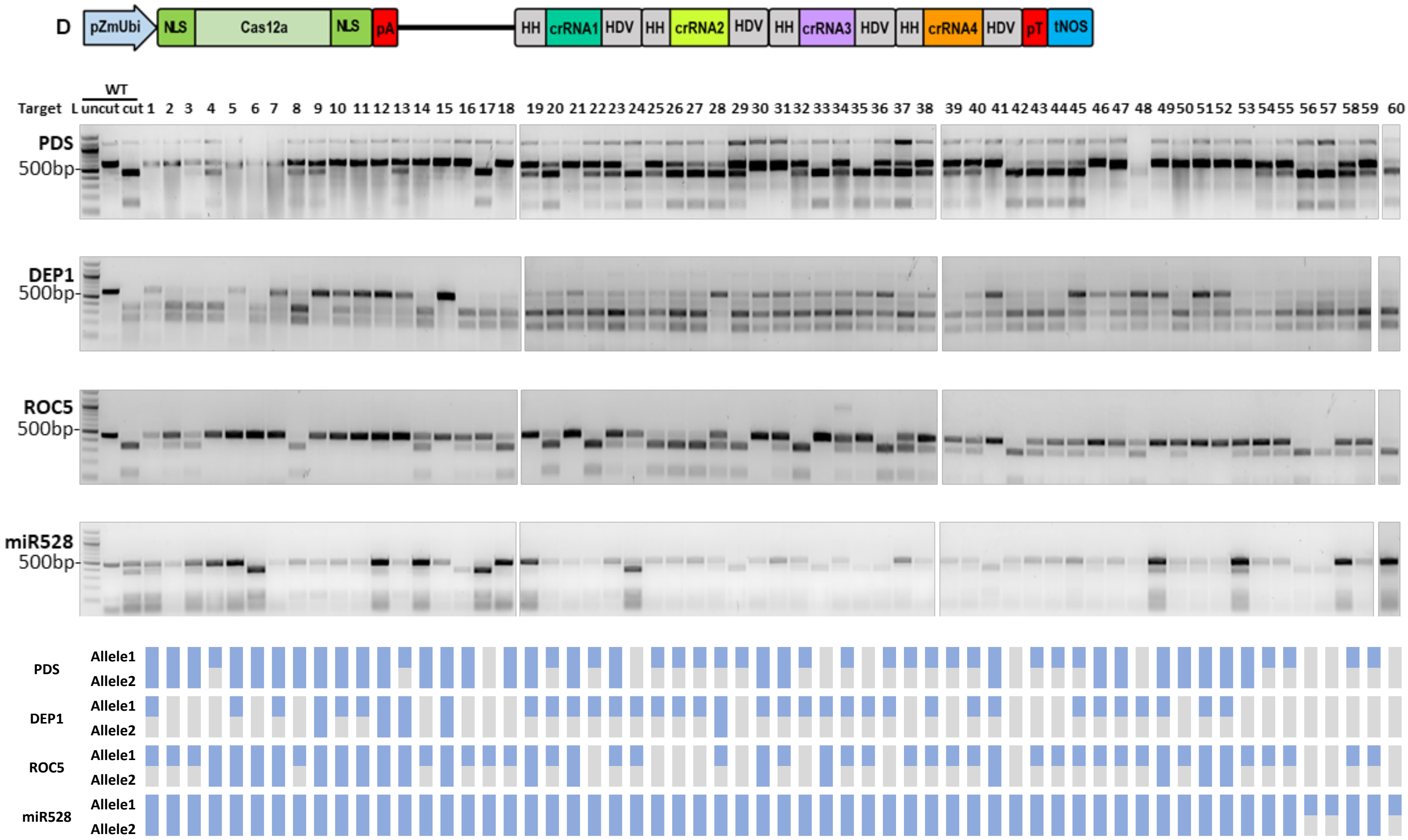

Supplementary Fig. 15. Analysis of multiplexing system D at four target genes with 60 T_0 lines. An illustration of the multiplexing strategy is shown in the upper panel. The middle panel shows RFLP based genotyping data at four target sites among independent lines. L, NEB 100 bp DNA Ladder. WT, wild type rice plants. Uncut, PCR amplicon of target sites before restriction enzyme digestion. Cut, PCR amplicon of target sites after restriction enzyme digestion. The lower panel is a summary table showing editing (blue) or non-editing (gray) of both alleles based on RFLP and Sanger sequencing. Rice stable transformation was not repeated. Source data are provided as a Source Data file.

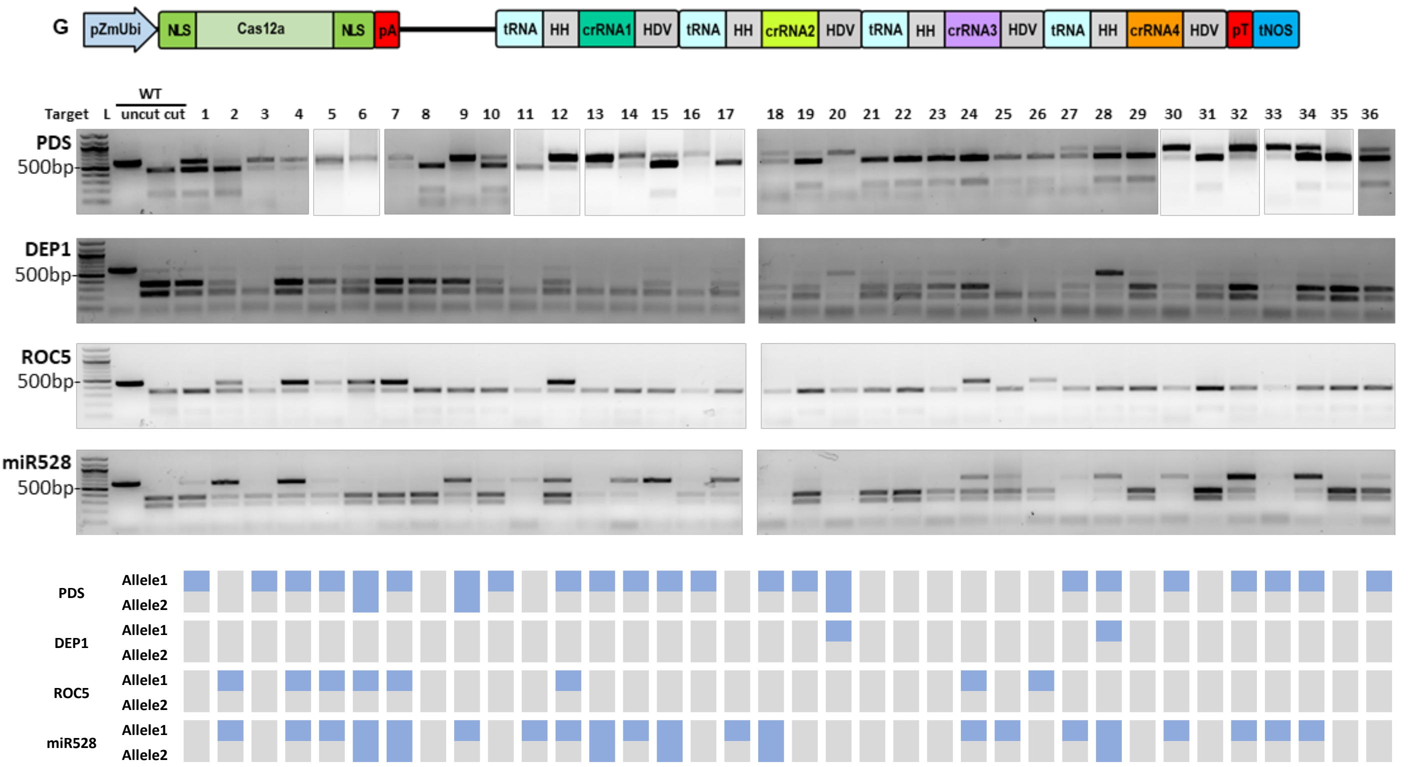

Supplementary Fig. 16. Analysis of multiplexing system G at four target genes with 36 T_0 lines. An illustration of the multiplexing strategy is shown in the upper panel. The middle panel shows RFLP based genotyping data at four target sites among independent lines. L, NEB 100 bp DNA Ladder. WT, wild type rice plants. Uncut, PCR amplicon of target sites before restriction enzyme digestion. Cut, PCR amplicon of target sites after restriction enzyme digestion. The lower panel is a summary table showing editing (blue) or non-editing (gray) of both alleles based on RFLP and Sanger sequencing. Rice stable transformation was not repeated. Source data are provided as a Source Data file.

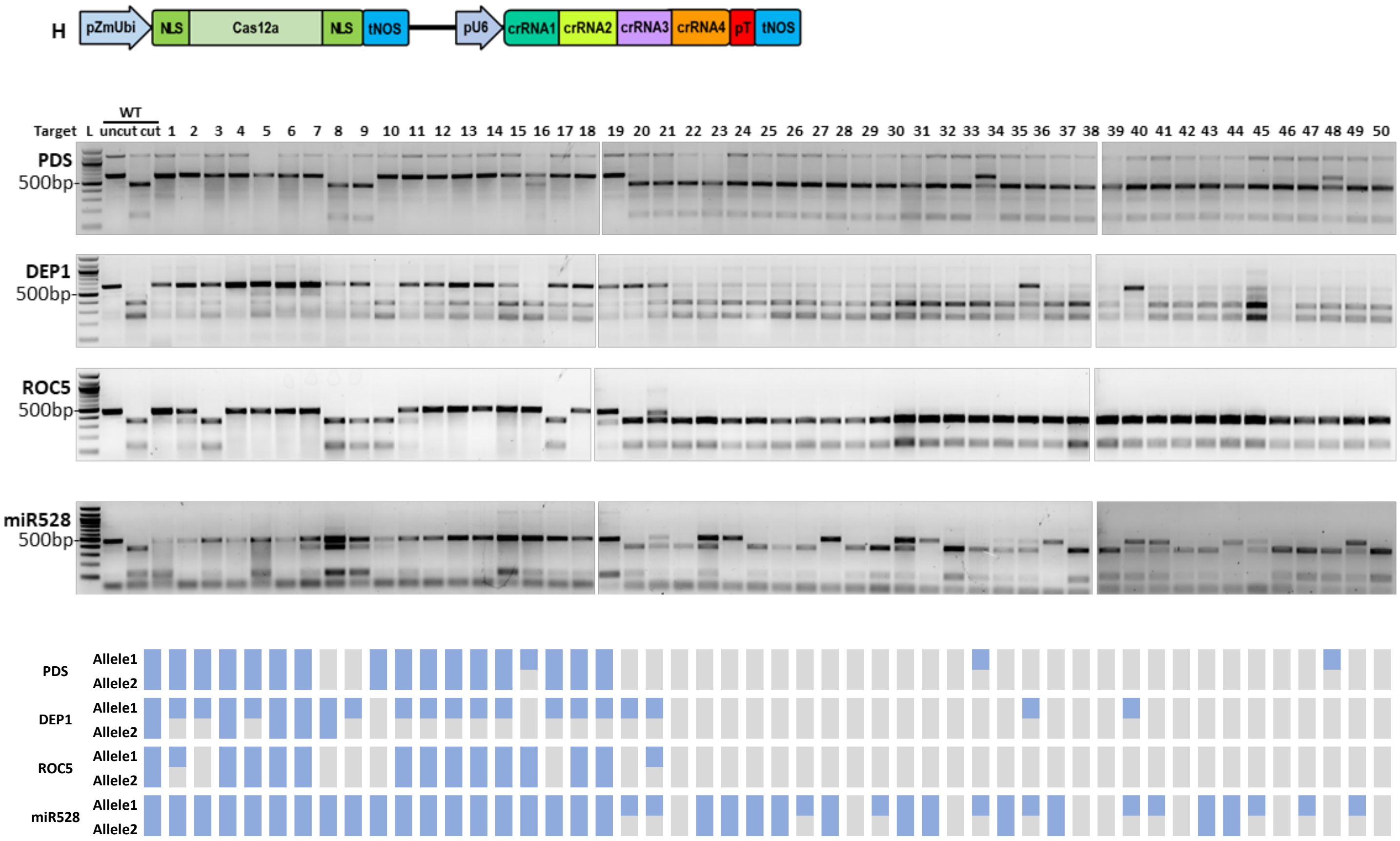

Supplementary Fig. 17. Analysis of multiplexing system H at four target genes with 50 T_0 lines. An illustration of the multiplexing strategy is shown in the upper panel. The middle panel shows RFLP based genotyping data at four target sites among independent lines. L, NEB 100 bp DNA Ladder. WT, wild type rice plants. Uncut, PCR amplicon of target sites before restriction enzyme digestion. Cut, PCR amplicon of target sites after restriction enzyme digestion. The lower panel is a summary table showing editing (blue) or non-editing (gray) of both alleles based on RFLP and Sanger sequencing. Rice stable transformation was not repeated. Source data are provided as a Source Data file.

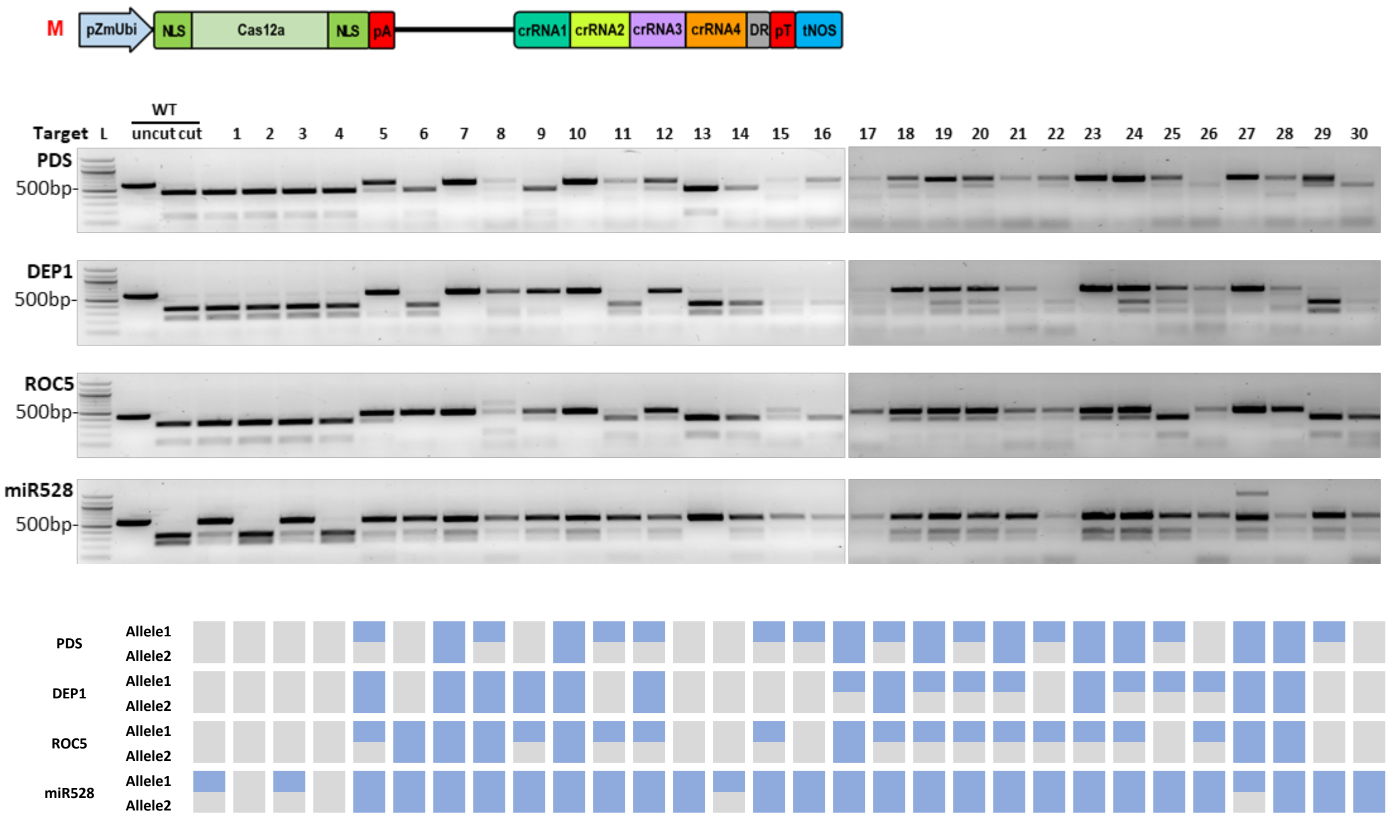

Supplementary Fig. 18. Analysis of multiplexing system M at four target genes with 30 T_0 lines. An illustration of the multiplexing strategy is shown in the upper panel. The middle panel shows RFLP based genotyping data at four target sites among independent lines. L, NEB 100 bp DNA Ladder. WT, wild type rice plants. Uncut, PCR amplicon of target sites before restriction enzyme digestion. Cut, PCR amplicon of target sites after restriction enzyme digestion. The lower panel is a summary table showing editing (blue) or non-editing (gray) of both alleles based on RFLP and Sanger sequencing. Rice stable transformation was not repeated. Source data are provided as a Source Data file.

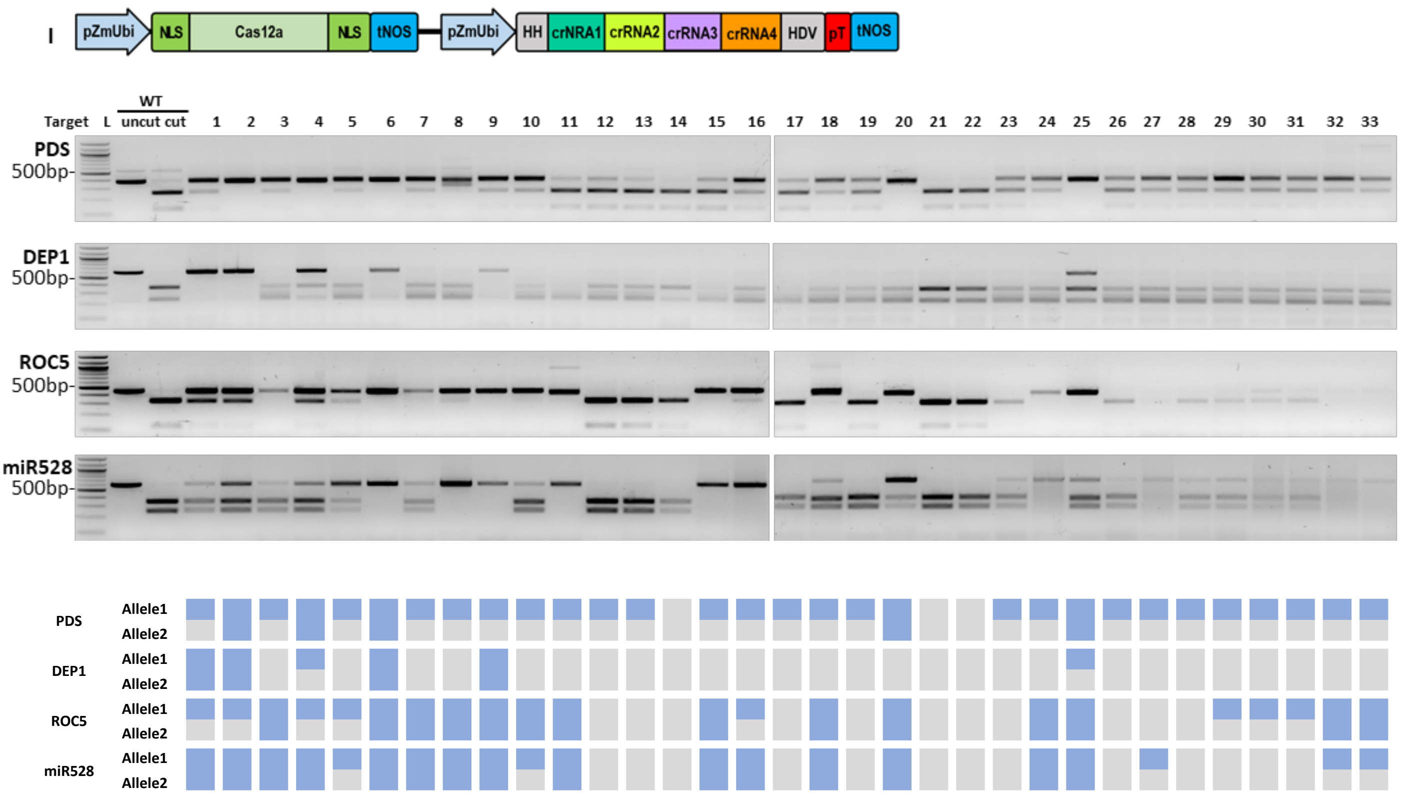

Supplementary Fig. 19. Analysis of multiplexing system I at four target genes with 33 T_0 lines. An illustration of the multiplexing strategy is shown in the upper panel. The middle panel shows RFLP based genotyping data at four target sites among independent lines. L, NEB 100 bp DNA Ladder. WT, wild type rice plants. Uncut, PCR amplicon of target sites before restriction enzyme digestion. Cut, PCR amplicon of target sites after restriction enzyme digestion. The lower panel is a summary table showing editing (blue) or non-editing (gray) of both alleles based on RFLP and Sanger sequencing. Rice stable transformation was not repeated. Source data are provided as a Source Data file.

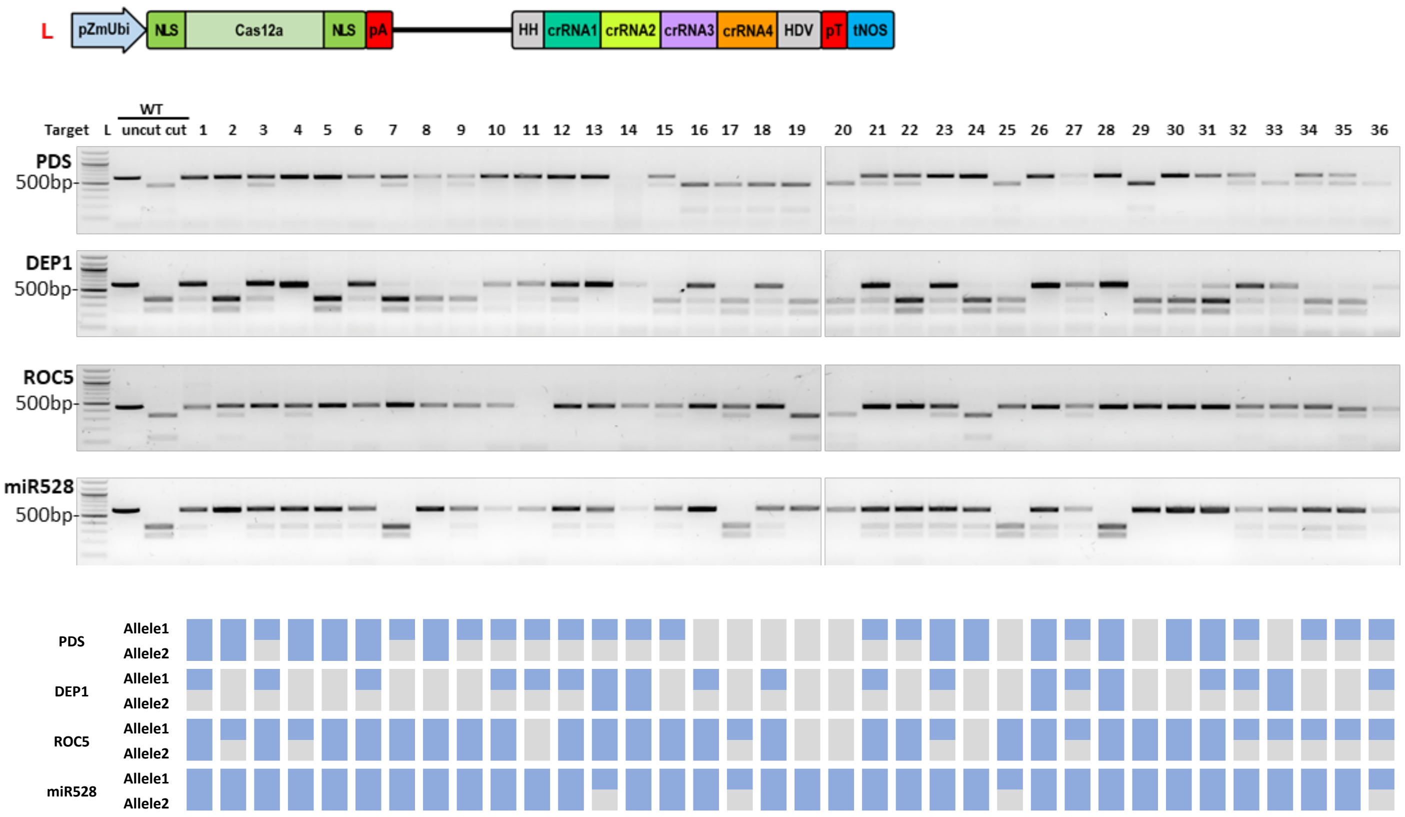

Supplementary Fig. 20. Analysis of multiplexing system L at four target genes with 36 T_0 lines. An illustration of the multiplexing strategy is shown in the upper panel. The middle panel shows RFLP based genotyping data at four target sites among independent lines. L, NEB 100 bp DNA Ladder. WT, wild type rice plants. Uncut, PCR amplicon of target sites before restriction enzyme digestion. Cut, PCR amplicon of target sites after restriction enzyme digestion. The lower panel is a summary table showing editing (blue) or non-editing (gray) of both alleles based on RFLP and Sanger sequencing. Rice stable transformation was not repeated. Source data are provided as a Source Data file.

```
OsPDS-TTTG

   WT  TGTGAGCTTTGGAGTGAAATCTCTTGTCTTAAGGAATAAA

Line1  TGTGAGCTTTGGAGTGAAATCT-----------------A  -17
       TGTGAGCTTTGGAGTGAAATCT-----------------A  -17
Line2  TGTGAGCTTTGGAGTGAAATC------------------A  -18
       TGTGAGCTTTGGAGTGAAATC------------------A  -18
Line3  TGTGAGCTTTGGAGTGAAATCTCTT-------GGAATAAA  -7
       TGTGAGCTTTGGAGTGAAATCTCTT-------GGAATAAA  -7
Line4  ---------36 bp deletion------------ATAAA  -36
       ---------36 bp deletion------------ATAAA  -36
Line5  TGTGAGCTTTGGAGTGAAATCTCTT--------GAATAAA  -8
       TGTGAGCTTTGGAGTGAAATCTCTT--------GAATAAA  -8
Line6  TGTGAGCTTTGGAGTGAAATCTCTT----------ATAAA  -10
       TGTGAGCTTTGGAGTGAAATCTCTT----------ATAAA  -10
Line7  TGTGAGCTTTGGAGTGAAATCTCTT-------GGAATAAA  -7
       TGTGAGCTTTGGAGTGAAATCTCTT-------GGAATAAA  -7
Line8  TGTGAGCTTTGGAGTGAAATCTCTT--------GAATAAA  -8
       TGTGAGCTTTGGAGTGAAATCT--26 bp deletion--  -26
Line9  TGTGAGCTTTGGAGTGAAATC------------------A  -18
       TGTGAGCTTTGGAGTGAAATC------------------A  -18
Line10 TGTGAGCTTTGGAGTGAAATC------------------A  -18
       TGTGAGCTTTGGAGTGAAATC------------------A  -18
Line11 TGTGAGC----------------------------ATAAA  -28
       TGTGAGCTTTGGAGTGAAATCTCTT--------GAATAAA  -8
Line12 TGTGAGCTTTGGAGTGAAATCTCTT-------GGAATAAA  -7
       TGTGAGCTTTGGAGTGAAATCTCTT--------GAATAAA  -8
       TGTGAGCTTTGGAGTGA-----------------AATAAA  -17
Line13 TGTGAGCTTTGGAGTGAAATCT-----------------A  -17
       TGTGAGCTTTGGAGTGAAATCT-----------------A  -17
Line14 TGTGAGCTTTGGAGTGAAATCTCTT----------ATAAA  -10
       TGTGAGCTTTGGAGTGAAATCTCTT----------ATAAA  -10
Line15 TGTGAGCTTTGGAGTGAAATCTCTT----------ATAAA  -10
       TGTGAGCTTTGGAGTGAAATCTCTT----------ATAAA  -10
Line16 TGTGAGCTTTGGAGTGAAATCTC----------GAATAAA  -10
       TGTGAGCTTTGGAGTGAAATCTC----------GAATAAA  -10
Line17 TGTGAGCTTTGGAGTGAAATCTC----------------A  -16
       TGTGAGCTTTGGAGTGAAATCTCT---------GAATAAA  -9
Line18 TGTGAGCTTTGGAGTGAAATCTCT---------GAATAAA  -9
       TGTGAGCTTTGGAGTGAAATCTCT---------GAATAAA  -9
Line19 TGTGAGCTTTGGAGTGAAATCTCT---------GAATAAA  -9
       TGTGAGCTTTGGAGTGAAATCTCT---------GAATAAA  -9
Line20 TGTGAGCTTTGGAGTGAAATCTCT---------GAATAAA  -9
       TGTGAGCTTTGGAGTGAAATCTCT---------GAATAAA  -9
Line21 TGTGAGC----------------------------ATAAA  -28
       TGTGAGCTTTGGAGTGAAATCTCTT--------GAATAAA  -8
Line22 TGTGAGCTTTGGAGTGAAATCTCTT-------GGAATAAA  -7
       TGTGAGCTTTGGAGTGAAATCTCTT-------GGAATAAA  -7
Line23 TGTGAGCTTTGGAGTGAAATCTCTT----------ATAAA  -10
       TGTGAGCTTTGGAGTGAAATCTCTT----------ATAAA  -10
Line24 TGTGAGCTTTGGAGTGAAATCTCTT----------ATAAA  -10
       TGTGAGCTTTGGAGTGAAATCTCTT----------ATAAA  -10
Line25 TGTGAGCTTTGGAGTGAAATCTCTT---TTC-GG------  -26/2
       TGTGAGCTTTGGAGTGAAATCTCTT---TTC-GG------  -26/2
Line26 TGTGAGCTTTGGAGT------------------GAATAAA  -18
       TGTGAGCTTTGGAGTGAAATCTCTTGTC---AGGAATAAA  -3
Line27 TGTGAGCTTTGGAGTGAAATCTCT---------GAATAAA  -9
       TGTGAGCTTTGGAGTGAAATCTCT---------GAATAAA  -9
Line28 TGTGAGCTTTGGAGTGA----------------GAATAAA  -16
       TGTGAGCTTTGGAGTGAAATCTCTT---------AATAAA  -9
Line29 TGTGAGCTTTGGAGTGA----------------GAATAAA  -16
       TGTGAGCTTTGGAGTGAAATCTCTT---------AATAAA  -9
Line30 TGTGAGCTTTGGAGTGAAA-----------A--GAATAAA  -13
       TGTGAGCTTTGGAGTGAAATCTCTT---------AATAAA  -9
Line31 TGTGAGCTTTGGAGTGAAATCTCTTGTC-----GAATAAA  -5
       TGTGAGCTTTGGAGTGAAATCTCTT---------AATAAA  -9
Line32 TGTGAGCTTTGGAGTGAAATCTCTT---TTC-GG------  -26/2
       TGTGAGCTTTGGAGTGAAATCTCTT---TTC-GG------  -26/2
Line33 TGTGAGCTTTGG------------------------TAAA  -24
       TGTGAGCTTTGGAGTGAAATCTCT------------TAAA  -12
Line34 TGTGAGCTTTGGAGTGAAATCTCT------------TAAA  -12
       TGTGAGCTTTGGAGTGAAATCTCTTGT------GAATAAA  -6
Line35 TGTGAGCTTTGGAGT------------------GAATAAA  -18
       TGTGAGCTTTGGAGTGAAATCTCTTGTC---AGGAATAAA  -3
Line36 TGTGAGCTTTGGAGTGAAA-----------A--GAATAAA  -13
       TGTGAGCTTTGGAGTGAAATCTCTT---------AATAAA  -9
```

```
OsDEP1-TTTG

   WT  GTCTTTGCTACTGTTGCAAGTGCTCACCCAAGTGCAAAAGACCAAGG

Line1  GTCTTTGCTACTGTTGC-------------AGTGCAAAAGACCAAGG  -13
       GTCTTTGCTACTGTTGCAAGT-------CAAGTGCAAAAGACCAAGG  -7
Line2  GTCTTTGCTACTGTTGCAAGTGCTTACCCAAGTGCAAAAGACCAAGG  1
       GTCTTTGCTACTGTTGCAAGTGCTCACCCAAGTGCAAAAGACCAAGG  WT
Line3  GTCTTTGCTACTGTTGCAAGTG-------AAGTGCAAAAGACCAAGG  -7
       GTCTTTGCTACTGTTGCAAGTG-------AAGTGCAAAAGACCAAGG  -7
Line4  GTCTTTGCTACTGTTGCAAGTG-T-----AAGTGCAAAAGACCAAGG  -6
       GTCTTTGCTACTGTTGCAAGTGCTCACCCAAGTGCAAAAGACCAAGG  WT
Line5  GTCTTTGCTACTGTTGCAAGT--------AAGTGCAAAAGACCAAGG  -8
       -----------48 bp deletion---------CAAAAGACCAAGG  -48
Line6  GTCTTTGCTACTGTTGCAAGTGCTCACCCAAGTGCAAAAGACCAAGG  WT
       GTCTTTGCTACTGTTGCAAGTGCTCACCCAAGTGCAAAAGACCAAGG  WT
Line7  GTCTTTGCTACTGTTGCAAG---------AAGTGCAAAAGACCAAGG  -9
       GTCTTTGCTACTGTTGCAAGTGCTCACCCAAGTGCAAAAGACCAAGG  WT
Line8  GTCTTTGCTACTGTTGCAAGTG--CACCCAAGTGCAAAAGACCAAGG  -2
       GTCTTTGCTACTGTTGCAAGTGCTCACCCAAGTGCAAAAGACCAAGG  WT
Line9  GTCTTTGCTACTGTTG------------CAAGTGCAAAAGACCAAGG  -12
       GTCTTTGCTACTGTTGCAAG----------AGTGCAAAAGACCAAGG  -10
Line10 GTCTTTGCTACTGT---------------AAGTGCAAAAGACCAAGG  -15
       GTCTTTGCTACTGTTGCAAGTGCTCACCCAAGTGCAAAAGACCAAGG  WT
Line11 -----------43 bp deletion--------------GACCAAGG  -43/1/+2
       -----------43 bp deletion--------------GACCAAGG  -43/1/+2
Line12 GTCTTTGCTACTGT------------------TGCAAAAGACCAAGG  -18
       GTCTTTGCTACTGTTGCAAGTGCTCACCCAAGTGCAAAAGACCAAGG  WT
Line13 GTCTTTGCTACTGTTGCAA---------CAAGTGCAAAAGACCAAGG  -9
       GTCTTTGCTACTGTTGCAAGT-------CAAGTGCAAAAGACCAAGG  -7
       GTCTTTGCTACTGTTGCAAGTGCTCACCCAAGTGCAAAAGACCAAGG  WT
Line14 GTCTTTGCTACTGTTGCAAGT--------AAGTGCAAAAGACCAAGG  -8
       GTCTTTGCTACTGTTGCAAGTGCTCACCCAAGTGCAAAAGACCAAGG  WT
Line15 GTCTTTGCTACTGTTG------------CAAGTGCAAAAGACCAAGG  -12
       GTCTTTGCTACTGTTGCAAGTGCTCACCCAAGTGCAAAAGACCAAGG  WT
Line16 GT-------------------------------GCAAAAGACCAAGG  -31
       GTCTTTGCTACTGTTG------------CAAGTGCAAAAGACCAAGG  -12
Line17 GTCTTT----------CAAGT-CTGA---AAGTGCAAAAGACCAAGG  -14/1
       GTCTTTGCTACTGTT----------ACCCAAGTGCAAAAGACCAAGG  -10
Line18 GTCT-----------------------------GCAAAAGACCAAGG  -29
       GTCTTTGCTACTGTTGCA-----------AAGTGCAAAAGACCAAGG  -11
Line19 GTCT-----------------------------GCAAAAGACCAAGG  -29
       GTCTTTGCTACTGTTGCA-----------AAGTGCAAAAGACCAAGG  -11
Line20 GTCT-----------------------------GCAAAAGACCAAGG  -29
       GTCTTTGCTACTGTT---------------AGTGCAAAAGACCAAGG  -15
Line21 -----------43 bp deletion--------------GACCAAGG  -43/1/+2
       -----------43 bp deletion--------------GACCAAGG  -43/1/+2
Line22 GTCTTTGCTACTGTTGCAAGTG-------AAGTGCAAAAGACCAAGG  -7
       GTCTTTGCTACTGTTGCAAGTG-------AAGTGCAAAAGACCAAGG  -7
Line23 GTCTTTGCTACTG---------------------CAAAAGACCAAGG  -21
       GTCTTTGCTACTGTTGCAAGTGCTCACCCAAGTGCAAAAGACCAAGG  WT
Line24 GTCTTTGCTACTGTTG------------CAAGTGCAAAAGACCAAGG  -12
       GTCTTTGCTACTGTTGCAAGTGCTCACCCAAGTGCAAAAGACCAAGG  WT
Line25 GTCTTTGCTACTGTTGCAA-TGCTCACCCAAGTGCAAAAGACCAAGG  -1
       GTCTTTGCTACTGTTGCAAGTGCTCACCCAAGTGCAAAAGACCAAGG  WT
Line26 GTCTTTGCTACTGTTG------------CAAGTGCAAAAGACCAAGG  -12
       GTCTTTGCTACTGTTGCAAG---------AAGTGCAAAAGACCAAGG  -9
Line27 GTCTTTGCTACTGTTG--------------------------CAAGG  -26
       GTCT-----------------------------GCAAAAGACCAAGG  -29
Line28 GTCTTTGCTACTGT---------------------------------  -33
       GTCTTTGCTACTGTTG------------CAAGTGCAAAAGACCAAGG  -12
Line29 GTCTTTGCTACTGT---------------------------------  -33
       GTCTTTGCTACTGTTGCAAGTG--CACCCAAGTGCAAAAGACCAAGG  -2
Line30 GTCTTTGCTACTGTTGCA---------------GCAAAAGACCAAGG  -15
       ----------139 bp deletion-------TGCAAAAGACCAAGG  -139
Line31 GTCTTTGCTACTGTTG------------CAAGTGCAAAAGACCAAGG  -12
       GTCTTTGCTACTGTTG------------CAAGTGCAAAAGACCAAGG  -12
Line32 GTCTTTGCTACTGTTG------------CAAGTGCAAAAGACCAAGG  -12
       GTCTTTGCTACTGTTGCAAGTGCTCACCCAAGTGCAAAAGACCAAGG  WT
Line33 GTCTTTGCTACTGTTGCAAGTG---------GTGCAAAAGACCAAGG  -9
       GTCTTT-------TTGCATTTTAAA----AAGTGCAAAAGACCAAGG  -11/7
Line34 GTCTTTGCTA----------------------------------AGG  -34
       GTCTTTGCTACTGTTGCAAGTGCT----CAAGTGCAAAAGACCAAGG  -4
Line35 GTCTTTGCTACTGTTG------------CAAGTGCAAAAGACCAAGG  -12
       GTCTTTGCTACTGTTGCAAG---------AAGTGCAAAAGACCAAGG  -9
Line36 GTCTTTGCTACTGTTGCA---------------GCAAAAGACCAAGG  -15
       GTCTTTGCTACTGT------------------TGCAAAAGACCAAGG  -18
```

Supplementary Fig. 21. Genotypes of T_0 rice plants for multiplexing system B at four target genes. PCR amplicons were sequenced using the Hi-TOM platform. PAM sequences are in red. Protospacer sequences are in blue. Substituted nucleotides are in green.

OsROC5-TTTC

```
   WT  GCCATTTCTGCTTCCTGCAATGCCGGTAGACACCTCCTCA

Line1  GCCATTTCTGCTTCCTGC-------------------TCA  -19
       GCCATTTCTGCTTCCTGCAATG----------CCTCCTCA  -10
Line2  GCCATTTCTGCTTCCTGC-------------------TCA  -19
       GCCATTTCTGCTTCCTGCAATG----------CCTCCTCA  -10
Line3  GCCATTTCTGCTTCCTGC-------------------TCA  -19
       GCCATTTCTGCTTCCTGCAATG----------CCTCCTCA  -10
Line4  GCCATTTCTGCTTCCTGC-------------------TCA  -19
       GCCATTTCTGCTTCCTGCAATG----------CCTCCTCA  -10
Line5  GCCATTTCTGCTTCCTGC-------------------TCA  -19
       GCCATTTCTGCTTCCTGCAATG----------CCTCCTCA  -10
Line6  GCCATTTCTGCTTCCTGC-------------------TCA  -19
       GCCATTTCTGCTTCCTGCAATG----------CCTCCTCA  -10
Line7  GCCATTTCTGCTTCCTGC-------------------TCA  -19
       GCCATTTCTGCTTCCTGCAATG----------CCTCCTCA  -10
Line8  GCCATTTCTGCTTCCTGC-------------------TCA  -19
       GCCATTTCTGCTTCCTGCAATG----------CCTCCTCA  -10
Line9  GCCATTTCTGCTTCCTGC-------------------TCA  -19
       GCCATTTCTGCTTCCTGCAATG----------CCTCCTCA  -10
Line10 GCCATTTCTGCTTCCTGC-------------------TCA  -19
       GCCATTTCTGCTTCCTGCAATG----------CCTCCTCA  -10
Line11 GCCATTTCTGCTTCCTGCAA------TA-ACACCTCCTCA  -7
       GCCATTTCTGCTTCCTGCAATG--------CACCTCCTCA  -8
Line12 GCCATTTCTGCTTCCTGC-------------------TCA  -19
       GCCATTTCTGCTTCCTGCAATG----------CCTCCTCA  -10
Line13 GCCATTTCTGCTTCCTGC-------------------TCA  -19
       GCCATTTCTGCTTCCTGCAATG----------CCTCCTCA  -10
Line14 GCCATTTCTGCTTCCTGC-------------------TCA  -19
       GCCATTTCTGCTTCCTGCAATG----------CCTCCTCA  -10
Line15 GCCATTTCTGCTTCCTGC-------------------TCA  -19
       GCCATTTCTGCTTCCTGCAATG----------CCTCCTCA  -10
Line16 GCCATTTCTGCTTCCTGCAAT-------------TCCTCA  -13
       GCCATTTCTGCTTCCTGCAAT---------------CTCA  -15
Line17 GCCATTTCTGCTTCCTGCAATG----------CCTCCTCA  -10
       GCCATTTCTGCTTCCTGCAAT-------------TCCTCA  -13
       GCCATTTCTGCTTCCTGC-------------------TCA  -19
Line18 GCCATTTCTGCTTCCTGC-------------------TCA  -19
       GCCATTTCTGCTTCCTGCAATG----------CCTCCTCA  -10
Line19 GCCATTTCTGCTTCCTGC-------------------TCA  -19
       GCCATTTCTGCTTCCTGCAATG----------CCTCCTCA  -10
Line20 GCCATTTCTGCTTCCTGC-------------------TCA  -19
       GCCATTTCTGCTTCCTGCAATG----------CCTCCTCA  -10
Line21 GCCATTTCTGCTTCCTGCAA------TA-ACACCTCCTCA  -7
       GCCATTTCTGCTTCCTGCAATG--------CACCTCCTCA  -8
Line22 GCCATTTCTGCTTCCTGC-------------------TCA  -19
       GCCATTTCTGCTTCCTGCAATG----------CCTCCTCA  -10
Line23 GCCATTTCTGCTTCCTGC-------------------TCA  -19
       GCCATTTCTGCTTCCTGCAATG----------CCTCCTCA  -10
Line24 GCCATTTCTGCTTCCTGC-------------------TCA  -19
       GCCATTTCTGCTTCCTGCAATG----------CCTCCTCA  -10
Line25 GCCATTTCTGCTTCCTGC-------------------TCA  -19
       GCCATTTCTGCTTCCTGCAATG----------CCTCCTCA  -10
Line26 GCCATTTCTGCTTCCTG-----55 bp deletion----  -55
       GCCATTTCTGCTTCCTGCAATGC----A---ACCTCCTCA  -7
Line27 GCCATTTCTGCTTCCTGC-------------------TCA  -19
       GCCATTTCTGCTTCCTGCAATG----------CCTCCTCA  -10
Line28 GCCATTTCTGCTTCCTGCAATG----------CCTCCTCA  -10
       GCCATTTCTGCTTCCTGCAA------TA-ACACCTCCTCA  -7
Line29 GCCATTTCTGCTTCCTGCAATG----------CCTCCTCA  -10
       GCCATTTCTGCTTCCTGCAA------TA-ACACCTCCTCA  -7
Line30 GCCATTTCTGCTTCCTGCA-------------CCTCCTCA  -13
       GCCATTTCTGCTTCCTGCAATGCC-----------CCTCA  -11
Line31 GCCATTTCTGCTTCCTGCAAT----------ACCTCCTCA  -10
       GCCATTTCTGCTTCCTGCAATGC-------CACCTCCTCA  -7
Line32 GCCATTTCTGCTTCCTGC-------------------TCA  -19
       GCCATTTCTGCTTCCTGCAATG----------CCTCCTCA  -10
Line33 GCCATTTCTGCTTCCTGCAATG-----------CTCCTCA  -11
       GCCATTTCTGCTTCCTGCAATG-----------CTCCTCA  -11
Line34 GCCATTTCTGCTTCCTGCAAT---20 bp deletion--  -20
       GCCATTTCTGCTTCCTGCAAT--------ACACCTCCTCA  -8
Line35 GCCATTTCTGCTTCCTG-----55 bp deletion----  -55
       GCCATTTCTGCTTCCTGCAATGC----A---ACCTCCTCA  -7
Line36 GCCATTTCTGCTTCCTGCA-------------CCTCCTCA  -13
       GCCATTTCTGCTTCCTGCAATGCC-----------CCTCA  -11
```

OsmiR528-TTTG

```
   WT  CACTTTTGCCTCTCTCTCCTGTGCTTGCCTCTTCCATTCCTGCTGCT

Line1  CACTTTTGCCTCTCTCTCCTG----------TTCCATTCCTGCTGCT  -10
       CACTTTTGCCTCTCTCTCCTG----TGCCTCTTCCATTCCTGCTGCT  -4
Line2  CACTTTTGCCTCTCTCTCCTG----------TTCCATTCCTGCTGCT  -10
       CACTTTTGCCTCTCTCTCCTG----TGCCTCTTCCATTCCTGCTGCT  -4
Line3  CACTTTTGCCTCTCTCTCCTG----------TTCCATTCCTGCTGCT  -10
       CACTTTTGCCTCTCTCTCCTG----TGCCTCTTCCATTCCTGCTGCT  -4
Line4  CACTTTTGCCTCTCTCTCCTG----------TTCCATTCCTGCTGCT  -10
       CACTTTTGCCTCTCTCTCCTG----TGCCTCTTCCATTCCTGCTGCT  -4
Line5  CACTTTTGCCTCTCTCTCCTG----------TTCCATTCCTGCTGCT  -10
       CACTTTTGCCTCTCTCTCCTG----TGCCTCTTCCATTCCTGCTGCT  -4
Line6  CACTTTTGCCTCTCTCTCCTG----------TTCCATTCCTGCTGCT  -10
       CACTTTTGCCTCTCTCTCCTG----TGCCTCTTCCATTCCTGCTGCT  -4
Line7  CACTTTTGCCTCTCTCTCCTG----------TTCCATTCCTGCTGCT  -10
       CACTTTTGCCTCTCTCTCCTG----TGCCTCTTCCATTCCTGCTGCT  -4
Line8  CACTTTTGCCTCTCTCTCCTG----------TTCCATTCCTGCTGCT  -10
       CACTTTTGCCTCTCTCTCCTG----TGCCTCTTCCATTCCTGCTGCT  -4
Line9  CACTTTTGCCTCTCTCTCCTG----------TTCCATTCCTGCTGCT  -10
       CACTTTTGCCTCTCTCTCCTG----TGCCTCTTCCATTCCTGCTGCT  -4
Line10 CACTTTTGCCTCTCTCTCCTG----------TTCCATTCCTGCTGCT  -10
       CACTTTTGCCTCTCTCTCCTG----TGCCTCTTCCATTCCTGCTGCT  -4
Line11 CACTTTTGCCTCTCTCTCCTG----------TTCCATTCCTGCTGCT  -10
       CACTTTTGCCTCTCTCTCCTG----TGCCTCTTCCATTCCTGCTGCT  -4
Line12 CACTTTTGCCTCTCTCTCCTG----------TTCCATTCCTGCTGCT  -10
       CACTTTTGCCTCTCTCTCCTG----TGCCTCTTCCATTCCTGCTGCT  -4
Line13 CACTTTTGCCTCTCTCTCCTG----------TTCCATTCCTGCTGCT  -10
       CACTTTTGCCTCTCTCTCCTG----TGCCTCTTCCATTCCTGCTGCT  -4
Line14 CACTTTTGCCTCTCTCTCCTG----------TTCCATTCCTGCTGCT  -10
       CACTTTTGCCTCTCTCTCCTG----TGCCTCTTCCATTCCTGCTGCT  -4
Line15 CACTTTTGCCTCTCTCTCCTG----------TTCCATTCCTGCTGCT  -10
       CACTTTTGCCTCTCTCTCCTG----TGCCTCTTCCATTCCTGCTGCT  -4
Line16 CACTTTTGCCTCTCTCTCCTG----TGCCTCTTCCATTCCTGCTGCT  -4
       CACTTTTGCCTCTCTCTCCTG--------ACTTCCATTCCTGCTGCT  -8/1
Line17 CACTTTTGCCTCTCTCTCCTG----TGCCTCTTCCATTCCTGCTGCT  -4
       CACTTTTGCCTCTCTCTCCTG--------ACTTCCATTCCTGCTGCT  -8/1
Line18 CACTTTTGCCTCTCTCTCCTG----------TTCCATTCCTGCTGCT  -10
       CACTTTTGCCTCTCTCTCCTG----TGCCTCTTCCATTCCTGCTGCT  -4
Line19 CACTTTTGCCTCTCTCTCCTG----------TTCCATTCCTGCTGCT  -10
       CACTTTTGCCTCTCTCTCCTG----TGCCTCTTCCATTCCTGCTGCT  -4
Line20 CACTTTTGCCTCTCTCTCCTG----------TTCCATTCCTGCTGCT  -10
       CACTTTTGCCTCTCTCTCCTG----TGCCTCTTCCATTCCTGCTGCT  -4
Line21 CACTTTTGCCTCTCTCTCCTG----TGCCTCTTCCATTCCTGCTGCT  -4
       CACTTTTGCCTCTCTCTCCTG----TGCCTCTTCCATTCCTGCTGCT  -4
Line22 CACTTTTGCCTCTCTCTCCTG----------TTCCATTCCTGCTGCT  -10
       CACTTTTGCCTCTCTCTCCTG----TGCCTCTTCCATTCCTGCTGCT  -4
Line23 CACTTTTGCCTCTCTCTCCTG----------TTCCATTCCTGCTGCT  -10
       CACTTTTGCCTCTCTCTCCTG----TGCCTCTTCCATTCCTGCTGCT  -4
Line24 CACTTTTGCCTCTCTCTCCTG----------TTCCATTCCTGCTGCT  -10
       CACTTTTGCCTCTCTCTCCTG----TGCCTCTTCCATTCCTGCTGCT  -4
Line25 CACTTTTGCCTCTCTCTCCTG----------TTCCATTCCTGCTGCT  -10
       CACTTTTGCCTCTCTCTCCTG----TGCCTCTTCCATTCCTGCTGCT  -4
Line26 CACTTTTGCCTCTCTCTCCTG----TGCCTCTTCCATTCCTGCTGCT  -4
       CACTTTTGCCTCTCTCTCCTG----TGCCTCTTCCATTCCTGCTGCT  -4
Line27 CACTTTTGCCTCTCTCTCCTG----------TTCCATTCCTGCTGCT  -10
       CACTTTTGCCTCTCTCTCCTG----TGCCTCTTCCATTCCTGCTGCT  -4
Line28 CACTTTTGCCTCTCTCTCCTG----TGCCTCTTCCATTCCTGCTGCT  -4
       CACTTTTGCCTCTCTCTCCTG----TGCCTCTTCCATTCCTGCTGCT  -4
Line29 CACTTTTGCCTCTCTCTCCTG----TGCCTCTTCCATTCCTGCTGCT  -4
       CACTTTTGCCTCTCTCTCCTG----TGCCTCTTCCATTCCTGCTGCT  -4
Line30 CACTTTTGCCTCTCTCTCCTG----TGCCTCTTCCATTCCTGCTGCT  -4
       CACTTTTGCCTCT---------------CTCTTCCATTCCTGCTGCT  -15
Line31 CACTTTTGCCTCTCTCTCCTG----TGCCTCTTCCATTCCTGCTGCT  -4
       CACTTTTGCCTCTCTCTCCTG----TGCCTCTTCCATTCCTGCTGCT  -4
Line32 CACTTTTGCCTCTCTCTCCTG----------TTCCATTCCTGCTGCT  -10
       CACTTTTGCCTCTCTCTCCTG----TGCCTCTTCCATTCCTGCTGCT  -4
Line33 CACTTTTGCCTCTCTCTCCTG--------TCTTCCATTCCTGCTGCT  -8
       CACTTTTGCCTCTCTCTCCTG--------TCTTCCATTCCTGCTGCT  -8
Line34 CACTTTTGCCTCTCTCTCCTG----TGCCTCTTCCATTCCTGCTGCT  -4
       CACTTTTGCCTCTCTCTCCTG----TGCCTCTTCCATTCCTGCTGCT  -4
Line35 CACTTTTGCCTCTCTCTCCTG----TGCCTCTTCCATTCCTGCTGCT  -4
       CACTTTTGCCTCTCTCTCCTG----TGCCTCTTCCATTCCTGCTGCT  -4
Line36 CACTTTTGCCTCT---------------CTCTTCCATTCCTGCTGCT  -15
       CACTTTTGCCTCTCTCTCCTGTGC------CTTCCATTCCTGCTGCT  -6
```

Supplementary Fig. 22. Genotypes of additional T_0 rice plants for multiplexing system B at four target genes. PCR amplicons were sequenced using the Hi-TOM platform. PAM sequences are in red. Protospacer sequences are in blue. Substituted nucleotides are in green.

```
OsPDS-TTTG

    WT  TGTGAGCTTTGGAGTGAAATCTCTTGTCTTAAGGAATAAA

Line1   TGTGAGCTTTGGAGTGAAATCTCT-----------ATAAA  -11
        TGTGAGCTTTGGAGTGAAATCTCTTG-------GAATAAA  -7
Line2   TGTGAGCTTTGGAGTGAAATCTC---------------AA  -15
        TGTGAGCTTTGGAGTGAAAT-----------------AAA  -17
Line3   TGTGAGCTTTGGAGTGAAATCTCTTG--------AATAAA  -8
        TGTGAGCTTTGGAGTGAAATCTCTT----------ATAAA  -10
Line4   TGTGAGCTTTGGAGTGAAATCTCTT---------AATAAA  -9
        TGTGAGCTTTGGAGTGA-----------------AATAAA  -17
Line5   TGTGAGCTTTGGAGTGAAATCTCTTGT--------ATAAA  -8
        TGTATCGTGTGAT----------------------TAAA   -23/10
Line6   TGTGAGCTTTGGAGTGAAATCTCTTGTCTTAAGGAATAAA   WT
        TGTGAGCTTTGGAGTGAAATCTCTT--------GAATAAA  -8
Line9   TGTGAGCTTTGGAGTGAAATCTCTTGTC------AATAAA  -6
        TGTGAGCTTTGGAGTGAAA---72 bp deletion----  -72
Line10  TGTGAGCTTTGGAGTGA--------------------AAA  -20
        TGTGAGCTTTGGAGTGAAATCTCTTGT------GAATAAA  -6
Line11  TGTGAGCTTTGGAGTGAAATCTCTTGT------GAATAAA  -6
        TGTGAGCTTTGGAGTGAAATCTCTTGT------GAATAAA  -6
```

```
OsDEP1-TTTG

    WT  GTCTTTGCTACTGTTGCAAGTGCTCACCCAAGTGCAAAAGACCAAGG

Line1   GTCTTTGCTACTGTTGCAAG----------------AAAGACCAAGG  -16
        GTCTTTGCTACTGTTGCAAG--------------------------G  -26
Line2   GTCTTTGCTACTGTTGCAAGT--------AAGTGCAAAAGACCAAGG  -8
        GTCTTTGCTACTGTTGCAAGTGCATCACCCAAGTGCAAAAGACCAAGG +1
Line3   GTCTTTGCTACTGTTGCAA---------CAAGTGCAAAAGACCAAGG  -9
        GTCTTTGCTACTGTTGCAA---------------------------G  -27
Line4   GTCTTTGCTACTGTTGCAAGT--------AAGTGCAAAAGACCAAGG  -8
        GTCTTTGCTACTGTTGCAAGTGCCTCACCCAAGTGCAAAAGACCAAGG +1
Line5   GTCTTTGCTACTGTTG----------------TGCAAAAGACCAAGG  -16
        GTCTTTGCTACTGTTGCAAGTG------CAAGTGCAAAAGACCAAGG  -6
Line6   GTCTTTGCTACTGTTG----------------TGCAAAAGACCAAGG  -16
        GTCTTTGCTACTGTTGCAAGTG------CAAGTGCAAAAGACCAAGG  -6
Line7   GTCTTTGCTACTGTTGCAA------------------AAGACCAAGG  -18
        GTCTTTGCTACTGTTGCAAGTG------CAAGTGCAAAAGACCAAGG  -6
Line9   GTCTTTGCTACTGTTG----------------TGCAAAAGACCAAGG  -16
        GTCTTTGCTACTGTTGCAAGTG------CAAGTGCAAAAGACCAAGG  -6
Line10  GTCTTTGCTACTGTTG----------------TGCAAAAGACCAAGG  -16
        GTCTTTGCTACTGTTGCAAGTG------CAAGTGCAAAAGACCAAGG  -6
Line11  GTCTTTGCTACTGTTG----------------TGCAAAAGACCAAGG  -16
        GTCTTTGCTACTGTTGCAAGTG------CAAGTGCAAAAGACCAAGG  -6
```

```
OsROC5-TTTC

    WT  GCCATTTCTGCTTCCTGCAATGCCGGTAGACACCTCCTCA

Line1   GCCATTTCTGCTTCCT---------------ACCTCCTCA  -15
        GCCATTTCTGCTTCCTGCAATG----------CCTCCTCA  -10
Line2   GCCATTTCTGCTTCCTGCAA------------CCTCCTCA  -12
        GCCATTTCTGCTTCCTGCAA------------CCTCCTCA  -12
Line3   GCCATTTCTGCTTCCTGCAATGCC-------ACCTCCTCA  -7
        GCCATTTCTGCTTCCTGCAATGCC--------CCTCCTCA  -8
Line4   GCCATTTCTGCTTCCTGCAA------------CCTCCTCA  -12
        GCCATTTCTGCTTCCTGCAA------------CCTCCTCA  -12
Line5   GCCATTTCTGCTTCCTGCAA-------------CTCCTCA  -13
        GCCATTTCTGCTTCCTGCAATGC-------CACCTCCTCA  -7
Line6   GCCATTTCTGCTTCCTGCAA-------------CTCCTCA  -13
        GCCATTTCTGCTTCCTGCAATGC-------CACCTCCTCA  -7
Line7   GCCATTTCTGCTTCCTGCAATGC-GGTAGACACCTCCTCA  -1
        GCCATTTCTGCTTCCTGCAATGC-------CACCTCCTCA  -7
Line8   GCCATTTCTGCTTCCTGCAATGC---------CCTCCTCA  -9
        GCCATTTCTGCTTCCTGCAATGC-----------TCCTCA  -11
Line9   GCCATTTCTGCTTCCTGCAA-------------CTCCTCA  -13
        GCCATTTCTGCTTCCTGCAATGC-------CACCTCCTCA  -7
Line10  GCCATTTCTGCTTCCTGCAA-------------CTCCTCA  -13
        GCCATTTCTGCTTCCTGCAATGC-------CACCTCCTCA  -7
Line11  GCCATTTCTGCTTCCTGCAA-------------CTCCTCA  -13
        GCCATTTCTGCTTCCTGCAATGC-------CACCTCCTCA  -7
```

```
OsmiR528-TTTG

    WT  CACTTTTGCCTCTCTCTCCTGTGCTTGCCTCTTCCATTCCTGCTGCT

Line1   CACTTTTGCCTCTCTCTCCTG---------------TTCCTGCTGCT  -15
        CACTTTTGCCTCTCTCTCCTG--CT----TCTTCCATTCCTGCTGCT  -6
Line2   CACTTTTGCCTCTCTCTCC----------TCTTCCATTCCTGCTGCT  -10
        CACTTTTGCCTCTCTCTCCAG----TGCCTCTTCCATTCCTGCTGCT  -4/1
Line3   CACTTTTGCCTCTCTCTCCT-----------------TCCTGCTGCT  -17
        CACTTTTGCCTCTCTCTCCT-----------------TCCTGCTGCT  -17
Line4   CACTTTTGCCTCTCTCTCC----------TCTTCCATTCCTGCTGCT  -10
        CACTTTTGCCTCTCTCTCCAG----TGCCTCTTCCATTCCTGCTGCT  -4/1
Line5   CACTTTTGCCTCTCTCTCCT-----------------TCCTGCTGCT  -17
        CACTTTTGCCTCTCTCTCCT-----------------TCCTGCTGCT  -17
Line6   CACTTTTGCCTCTCTCTCCT-----------------TCCTGCTGCT  -17
        CACTTTTGCCTCTCTCTCCT-----------------TCCTGCTGCT  -17
Line7   CACTT----------------------------CCATTCCTGCTGCT  -28
        CACTTTTGCCTCTCTCTCCTG---------CTTCCATTCCTGCTGCT  -9
Line8   CACTTTTGCCTCTCTCTCC-----------CTTCCATTCCTGCTGCT  -11
        CACTTTTGCCTCTCTCTCC-----------CTTCCATTCCTGCTGCT  -11
Line9   CACTTTTGCCTCTCTCTCCT-----------------TCCTGCTGCT  -17
        CACTTTTGCCTCTCTCTCCT-----------------TCCTGCTGCT  -17
Line10  CACTTTTGCCTCTCTCTCCT-----------------TCCTGCTGCT  -17
        CACTTTTGCCTCTCTCTCCT-----------------TCCTGCTGCT  -17
Line11  CACTTTTGCCTCTCTCTCCT-----------------TCCTGCTGCT  -17
        CACTTTTGCCTCTCTCTCCT-----------------TCCTGCTGCT  -17
```

Supplementary Fig. 23. Genotypes of T_0 rice plants edited by Mb2Cas12a with system B targeting four sites. PAM sequences are in red. Protospacer sequences are in blue. Substituted nucleotides are in green.

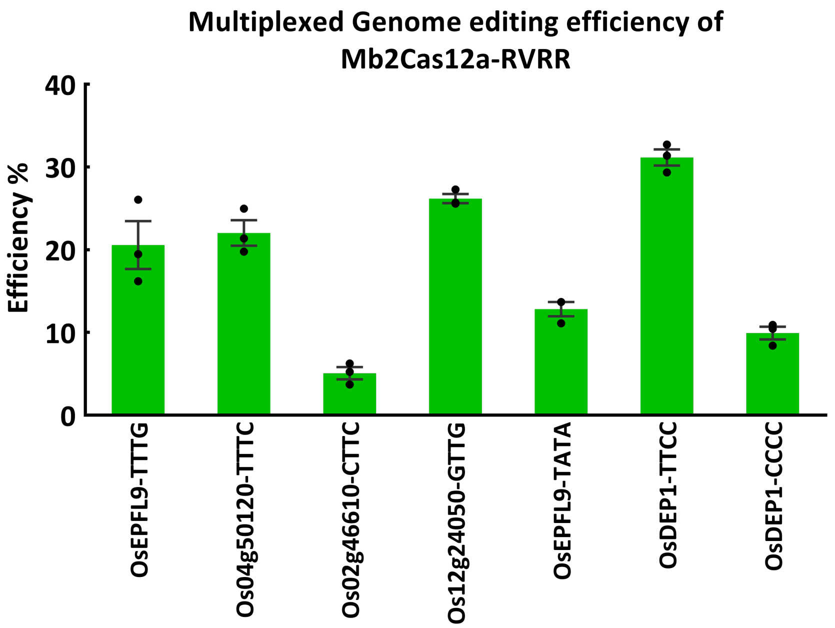

Supplementary Fig. 24. Multiplexed genome editing using Mb2Cas12a-RVRR variant and system B in rice protoplasts. Editing efficiencies were measured using RFLP analysis. Data are presented as mean values ± SEM. n=3 biologically independent samples. Source data are provided as a Source Data file.

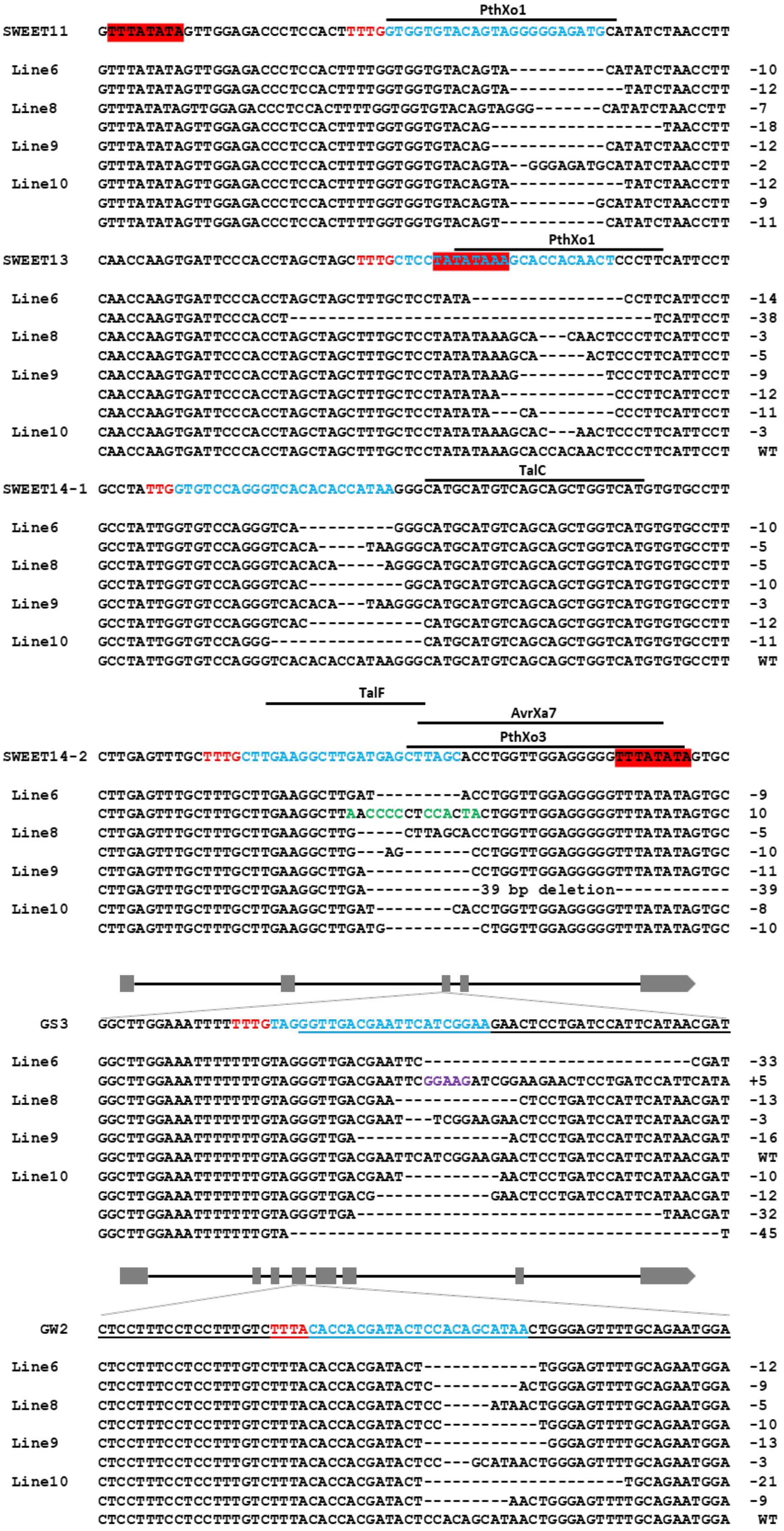

Supplementary Fig. 25. Genotypes of additional T_0 lines for simultaneous editing of six sites by Mb2Cas12a for multiplexed engineering of quantitative traits in rice. PAM sequences are in red. Protospacer sequences are in blue. Substituted nucleotides are in green. Inserted nucleotides are in purple. The TATA boxes are painted in red. The TAL effector binding sites in the promoters of the *SWEET* genes are indicated by solid black lines above the sequences. Exons of *GS3* and *GW2* are underlined.

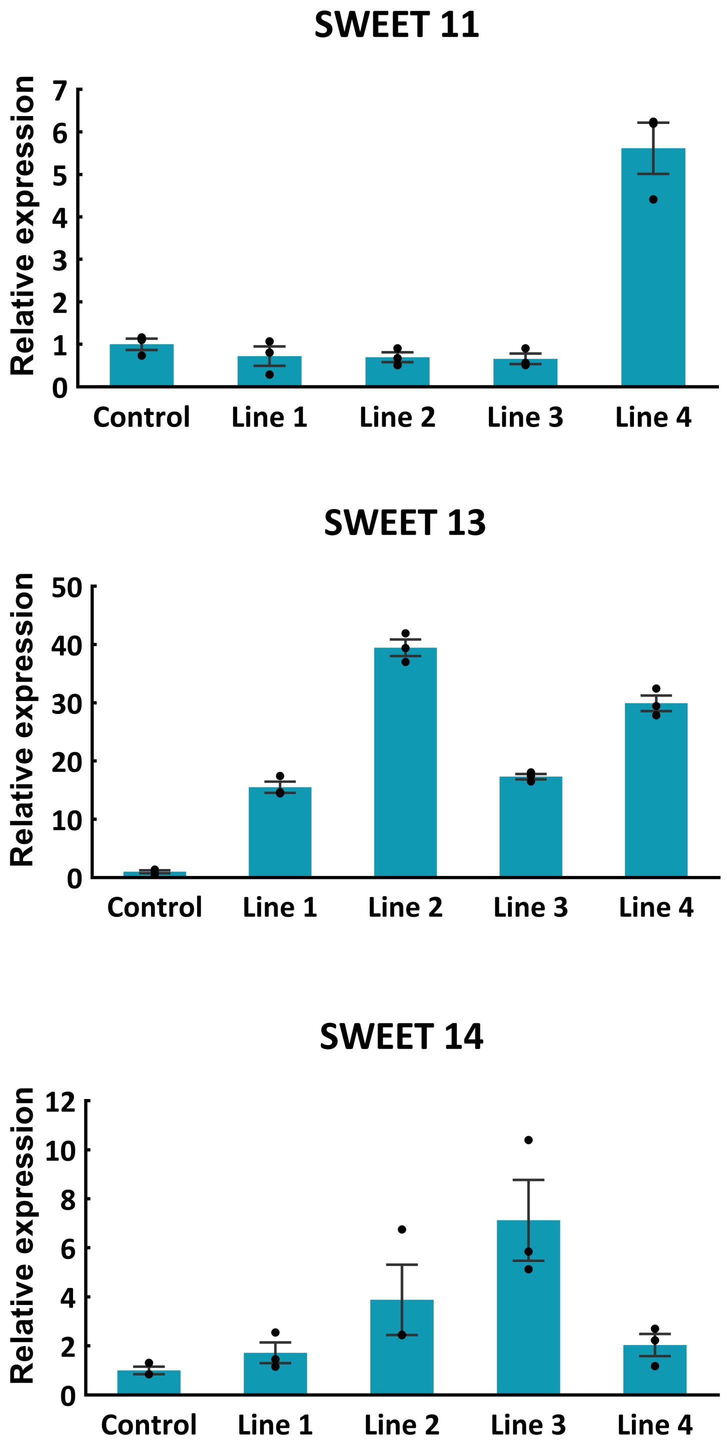

Supplementary Fig. 26. Relative expression of the *SWEET* genes in Mb2Cas12a edited rice lines. The rice line transformed with the T-DNA vector containing Mb2Cas12a without crRNAs were used as the control. Data are presented as mean values ± SEM. n=3 biologically independent samples. Source data are provided as a Source Data file.

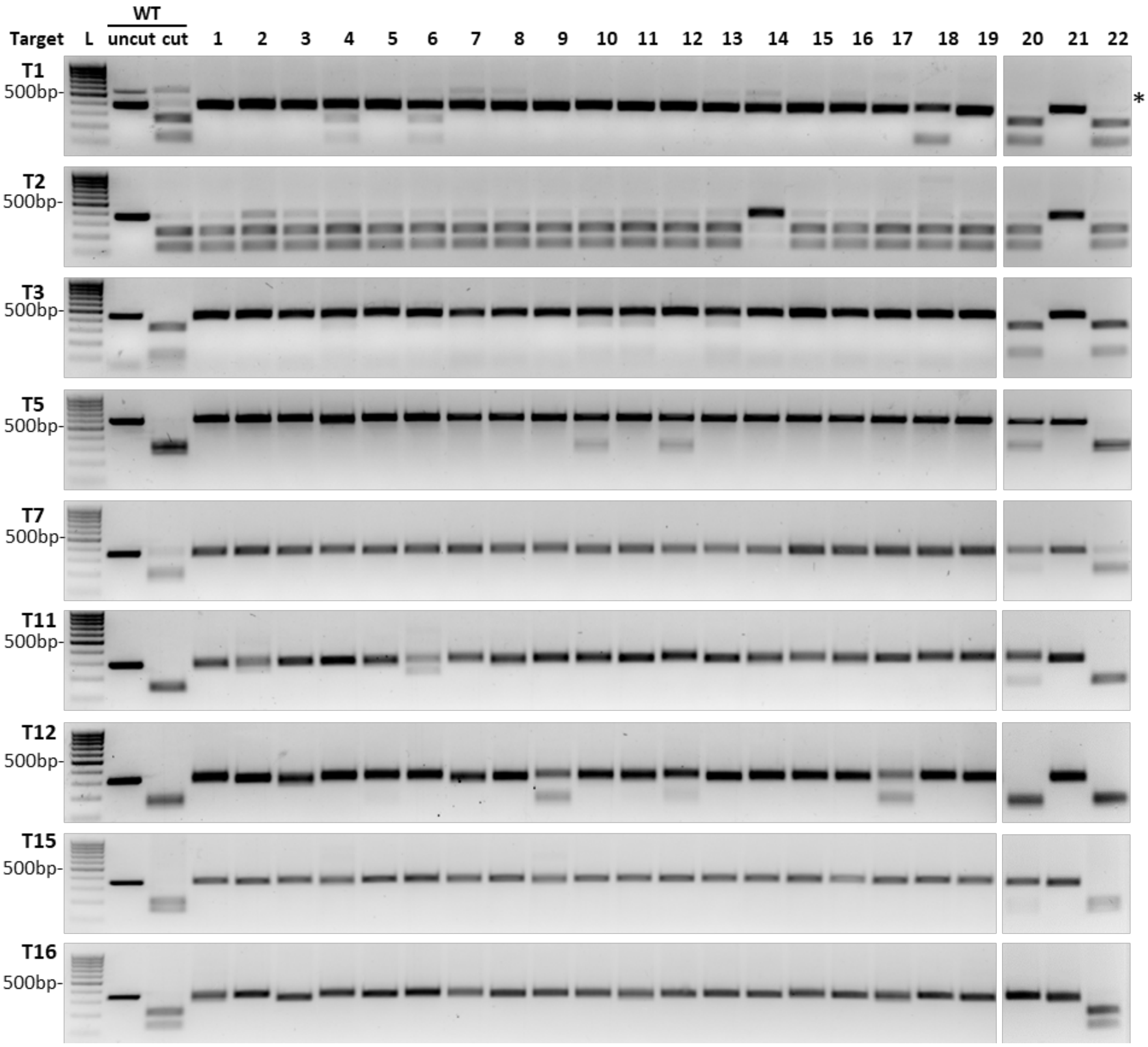

Supplementary Fig. 27. RFLP analysis of multiplexed large-scale genome editing at 9 target sites in T_0 rice plants. L, NEB 100 bp DNA Ladder. WT, wild type rice plants. Uncut, PCR amplicon of target sites before restriction enzyme digestion. Cut, PCR amplicon of target sites after restriction enzyme digestion. The asterisk indicates non-specific amplifications. Source data are provided as a Source Data file.

```
T1    WT AGCTTTGGAGTGAAATCTCTTGTCTTAAGGAATAAAGGAAAAAGA

Line1    AGCTTTGGAGTGAAATCTC----C-----GAATAAAGGAAAAAGA
         AGCTTTGGAGTGAAATCTCTT---------AATAAAGGAAAAAGA
Line3    AGCTTTGGAGTGAAATCTC-------------TAAAGGAAAAAGA
         AGCTTTGGAGTGAAATCT----------------AAGGAAAAAGA
Line4    AGCTTTGGAGTGAAATCTCT--------------AAGGAAAAAGA
         AGCTTTGGAGTGAAATC-----TCTTAAGGAATAAAGGAAAAAGA
Line5    AGCTTTGGAGTGAAATC-----------------AAGGAAAAAGA
         AGCTTTGGAGTGAAATCTC-------------TAAAGGAAAAAGA
Line6    AGCTTTGGAGTGAAATCTCTT-------GGAATAAAGGAAAAAGA
         AGCTTTGGAGTGAAATCT-------TAAG--ATAAAGGAAAAAGA
Line7    AGC-----A--------------------GAATAAAGGAAAAAGA
         AGCTTTGGAGTGAAATCTCTT-------GGAATAAAGGAAAAAGA
Line8    AGCTTTGGAGT--------------------------GAAAAAGA
         AGCTTTGGAGTG-------------------ATAAAGGAAAAAGA
Line9    AGCTTTGGAGT------------------GAATAAAGGAAAAAGA
         AGCTTTGGAGTGAAATCTC----------------AGGAAAAAGA
         AGCTTTGGAGTG-----------------------AGGAAAAAGA
Line10   AGCTTTGGAGTGAAATCTCTTGTC----GGAATAAAGGAAAAAGA
         AGCTTTGGAGTGAAATCTCTT--------GAATAAAGGAAAAAGA
Line11   AGCTTTGGAGTGAAATCTCTT-------GGAATAAAGGAAAAAGA
         AGCTTTGGAGTGAAATCTCTT--------GAATAAAGGAAAAAGA
Line12   AGCTTTGGAGTGAAATCTCTTGTC----GGAATAAAGGAAAAAGA
         AGCTTTGGAGTGAAATCTCTT--------GAATAAAGGAAAAAGA
Line13   AGCTTTGGAGTGAAATCTC-----------AATAAAGGAAAAAGA
         AGCTTTGGAGTGAAATCTCT----------AATAAAGGAAAAAGA
Line14   AGCTTTGGAGT--------------------------GAAAAAGA
         AGCTTTGGAGTGAAATCTCTTG--------AATAAAGGAAAAAGA
Line15   AGCTTTGGAGT--------------------------GAAAAAGA
         AGCTTTGGAGTGAAATCTCTT-------GGAATAAAGGAAAAAGA
Line16   AGCTTTGGAGT--------------------------GAAAAAGA
         AGCTTTGGAGTGAAATCTCTT--------GAATAAAGGAAAAAGA
Line17   AGCACTG--------------------------AAAGGAAAAAGA
         AGCTTTGGAGTGAAATCTCT----------AATAAAGGAAAAAGA
Line18   AGCTTTGGAGTGAAATCTCTT-------GGAATAAAGGAAAAAGA
         AGCTTTGGAGTGAAATCTCT--------GGAATAAAGGAAAAAGA
Line19   AGCTTTGGAGTGA-----------------AATAAAGGAAAAAGA
         AGCTTTGGAGTGAAAT------41 bp deletion---------
Line20   AGCTTTGGAGTGAAATCTCTTGTCTTAAGGAATAAAGGAAAAAGA
         AGCTTTGGAGTGAAATCTCTTGTCTTAAGGAATAAAGGAAAAAGA
```

```
T2    WT GTCTTTGCTACTGTTGCAAGTGCTCACCCAAGTGCAAAAGACCAA

Line1    GTCTTTGCTACTGTTGCAAGTGCTCACCCAAGTGCAAAAGACCAA
         GTCTTTGCTACTGTTGCAAGTGCTCACCCAAGTGCAAAAGACCAA
Line3    GTCTTTGCTACTGTTGCAAGTGCTCACCCAAGTGCAAAAGACCAA
         GTCTTTGCTACTGTTGCAAGTGCTCACCCAAGTGCAAAAGACCAA
Line4    GTCTTTGCTACTGTTGCAAGTGCTCACCCAAGTGCAAAAGACCAA
         GTCTTTGCTACTGTTGCAAGTGCTCACCCAAGTGCAAAAGACCAA
Line5    GTCTTTGCTACTGTTGCAAGTGCTCACCCAAGTGCAAAAGACCAA
         GTCTTTGCTACTGTTGCAAGTGCTCACCCAAGTGCAAAAGACCAA
Line6    GTCTTTGCTACTGTTGCAAGTGCTCACCCAAGTGCAAAAGACCAA
         GTCTTTGCTACTGTTGCAAGTGCTCACCCAAGTGCAAAAGACCAA
Line7    GTCTTTGCTACTGTTGCAAGTGCTCACCCAAGTGCAAAAGACCAA
         GTCTTTGCTACTGTTGCAAGTGCTCACCCAAGTGCAAAAGACCAA
Line8    GTCTTTGCTACTGTTGCAAGTGCTCACCCAAGTGCAAAAGACCAA
         GTCTTTGCTACTGTTGCAAGTGCTCACCCAAGTGCAAAAGACCAA
Line9    GTCTTTGCTACTGTTGCAAGTGCTCACCCAAGTGCAAAAGACCAA
         GTCTTTGCTACTGTTGCAAGTGCTCACCCAAGTGCAAAAGACCAA
Line10   GTCTTTGCTACTGTTGCAAGTGCTCACCCAAGTGCAAAAGACCAA
         GTCTTTGCTACTGTTGCAAGTGCTCACCCAAGTGCAAAAGACCAA
Line11   GTCTTTGCTACTGTTGCAAGTGCTCACCCAAGTGCAAAAGACCAA
         GTCTTTGCTACTGTTGCAAGTGCTCACCCAAGTGCAAAAGACCAA
Line12   GTCTTTGCTACTGTTGCAAGTGCTCACCCAAGTGCAAAAGACCAA
         GTCTTTGCTACTGTTGCAAGTGCTCACCCAAGTGCAAAAGACCAA
Line14   GTCTTTGCTACTGTTGCAAGTGCTCACCCAAGTGCAAAAGACCAA
         GTCTTTGCTACTGTTGCAAGTGC------------AAAAGACCAA
Line13   GTCTTTGCTACTGTTGCAAGTGCTCACCCAAGTGCAAAAGACCAA
         GTCTTTGCTACTGTTGCAAGTGCTCACCCAAGTGCAAAAGACCAA
Line15   GTCTTTGCTACTGTTGCAAGTGCTCACCCAAGTGCAAAAGACCAA
         GTCTTTGCTACTGTTGCAAGTGCTCACCCAAGTGCAAAAGACCAA
Line16   GTCTTTGCTACTGTTGCAAGTGCTCACCCAAGTGCAAAAGACCAA
         GTCTTTGCTACTGTTGCAAGTGCTCACCCAAGTGCAAAAGACCAA
Line17   GTCTTTGCTACTGTTGCAAGTGCTCACCCAAGTGCAAAAGACCAA
         GTCTTTGCTACTGTTGCAAGTGCTCACCCAAGTGCAAAAGACCAA
Line18   GTCTTTGCTACTGTTGCAAGTGCTCACCCAAGTGCAAAAGACCAA
         GTCTTTGCTACTGTTGCAAGTGCTCACCCAAGTGCAAAAGACCAA
Line19   GTCTTTGCTACTGTTGCAAGTGCTCACCCAAGTGCAAAAGACCAA
         GTCTTTGCTACTGTTGCAAGTGCTCACCCAAGTGCAAAAGACCAA
Line20   GTCTTTGCTACTGTTGCAAGTGCTCACCCAAGTGCAAAAGACCAA
         GTCTTTGCTACTGTTGCAAGTGCTCACCCAAGTGCAAAAGACCAA
```

```
T3    WT CCATTTCTGCTTCCTGCAATGCCGGTAGACACCTCCTCAAGCACT

Line1    CCATTTCTGCTTCCTGCA-------------CCTCCTCAAGCACT
         CCATTTCTGCTTCCTGCAATGC--------ACCTCCTCAAGCACT
Line3    CCATTTCTGCTTCCTGCA-------------------------CT
         CCATTTCTGCTTCCTGCAATG-------------CCTCAAGCACT
Line4    CCATTTCTGCTTCCTGCAATGCCGGTAGACACCTCCTCAAGCACT
         CCATTTCTGCTTCCTGCA-------------CCTCCTCAAGCACT
Line5    CCATTTCTGCTTCCTGCAATGC-----------TCCTCAAGCACT
         CCATTTCTGCTTCCTGCA-GAA----A-ACACCTCCTCAAGCACT
Line6    CCATTTCTGCTTCCTGCAATGC--------ACCTCCTCAAGCACT
         CCATTTCTGCTTCCTGCAATGCC--------CCTCCTCAAGCACT
         CCATTTCTGCTTCCTGCAATG-------ACACCTCCTCAAGCACT
         CCATTTCTGCTTCCTGCAATGCCGGTA--CACCTCCTCAAGCACT
Line7    CCATTTCTGCTTCCTGCAATGCCGGTAGACACCTCCTCAAGCACT
         CCATTTCTGCTTCCTGCAAT----------ACCTCCTCAAGCACT
         CCATTTCTGCTTCCTGCAATGCC----------TCCTCAAGCACT
Line8    CCATTTCTGCTTCCTGCAATGC--------ACCTCCTCAAGCACT
         CCATTTCTGCTTCCTGCAATG-------ACACCTCCTCAAGCACT
Line9    CCATTTCTGCTTCCTGCA-------------CCTCCTCAAGCACT
         CCATTTCTGCTTCCTGCAAT----------ACCTCCTCAAGCACT
Line10   CCATTTCTGCTTCCTGCAAT------------CTCCTCAAGCACT
         CCATTTCTGCTTCCTGCAATGCCGGTAGACACCTCCTCAAGCACT
Line11   CCATTTCTGCTTCCTGCAA-----------ACCTCCTCAAGCACT
         CCATTTCTGCTTCCTGCAATGCCGGTAGACACCTCCTCAAGCACT
Line12   CCATTTCTGCTTCCTGCAAT------------CTCCTCAAGCACT
         CCATTTCTGCTTCCTGCAATGCC-------ACCTCCTCAAGCACT
Line13   CCATTTCTGCTTCCTGCAATGC--------ACCTCCTCAAGCACT
         CCATTTCTGCTTCCTGCAATGCC--------CCTCCTCAAGCACT
         CCATTTCTGCTTCCTGCAATG-------ACACCTCCTCAAGCACT
Line14   CCATTTCTGCTTCCTGCAATG-------ACACCTCCTCAAGCACT
         CCATTTCTGCTTCCTGCAATG--------CACCTCCTCAAGCACT
Line15   CCATTTCTGCTTCCTGCAAT----------ACCTCCTCAAGCACT
         CCATTTCTGCTTCCTGCAA-----------ACCTCCTCAAGCACT
         CCATTTCTGCTTC-----------------------TCAAGCACT
Line16   CCATTTCTGCTTCCTGCAATGC--------ACCTCCTCAAGCACT
         CCATTTCTGCTTCCTGCAATG-------ACACCTCCTCAAGCACT
Line17   CCATTTCTGCTTCCT----------------------CAAGCACT
         CCATTTCTGCTTCCTGCAA-------------CTCCTCAAGCACT
Line18   CCATTTCTGCTTCCTGCAAT------------CTCCTCAAGCACT
         CCATTTCTGCTTCCTGCAATGCCG-------CCTCCTCAAGCACT
Line19   CCATTTCTGCTTCCTGCAATGC-------CACCTCCTCAAGCACT
         CCATTTCTGCTTCCTGCAATG-------ACACCTCCTCAAGCACT
Line20   CCATTTCTGCTTCCTGCAATGCCGGTAGACACCTCCTCAAGCACT
         CCATTTCTGCTTCCTGCAATGCCGGTAGACACCTCCTCAAGCACT
```

```
T4    WT ACTTTTGCCTCTCTCTCCTGTGCTTGCCTCTTCCATTCCTGCTGC

Line1    ACTTTTGCCTCTCTCTC------------CTTCCATTCCTGCTGC
         ACTTTTGCCTCTCTCTCCTG----TGCCTCTTCCATTCCTGCTGC
Line3    ACTTTTGCCTCTCTCTCCTGTGCTTGCCTCTTCCATTCCTGCTGC
         ACTTTTGCCTCTCTCTCCTGTGCTTGCCTCTTCCATTCCTGCTGC
Line4    ACTTTTGCCTCTCTCTC------------CTTCCATTCCTGCTGC
         ACTTTTGCCTCT---------------CTCTTCCATTCCTGCTGC
Line5    ACTTTTGCCTCTCTCTCCTGTG-------CTTCCATTCCTGCTGC
         ACTTTTGCCTCTCTCTC------------CTTCCATTCCTGCTGC
         ACTTTTGCCTCTCTCTCCTGTGCT----TCTTCCATTCCTGCTGC
Line6    ACTTTTGCCTCTCTCTC------------CTTCCATTCCTGCTGC
         ACTTTTGCCTCTCTCTC------------CTTCCATTCCTGCTGC
Line7    ACTTTTGCCTCTCTCTC------------CTTCCATTCCTGCTGC
         ACTTTTGCCTCTCTCTC------------CTTCCATTCCTGCTGC
Line8    ACTTTTGCCTCTCTCT----------CCTCTTCCATTCCTGCTGC
         ACTTTTGCCTCTCTCTCCTGTGCTTGCCTCTTCCATTCCTGCTGC
Line9    ACTTTTGCCTCTCTCTC------------CTTCCATTCCTGCTGC
         ACTTTTGCCTCTCTCTC------------CTTCCATTCCTGCTGC
Line10   ACTTTTGCCTCTCTCTCCTGTGCTTGCCTCTTCCATTCCTGCTGC
         ACTTTTGCCTCTCTCTCCTGTGCTTGCCTCTTCCATTCCTGCTGC
Line11   ACTTTTGCCTCTCTC-------31 bp deletion---------
         ACTTTTGCCTCTCTCTCCTG----------TTCCATTCCTGCTGC
Line12   ACTTTTGCCTCTCTCTCCTGTGCTTGCCTCTTCCATTCCTGCTGC
         ACTTTTGCCTCTCTCTCCTGTGCTTGCCTCTTCCATTCCTGCTGC
Line13   ACTTTTGC---------------------CTTCCATTCCTGCTGC
         ACTTTTGCCTCTCTCTCCTGT-------TCTTCCATTCCTGCTGC
Line14   ACTTTTGCCTCTCTCTCCTGTGCTTGCCTCTTCCATTCCTGCTGC
         ACTTTTGCCTCTCTCTCCTGTGCTTGCCTCTTCCATTCCTGCTGC
Line15   ACTTTTGCCTCTCTCTC------------CTTCCATTCCTGCTGC
         ACTTTTGCCTCTCTCTCCTG----TGCCTCTTCCATTCCTGCTGC
Line16   ACTTTTGCCTCTCTCT----------CCTCTTCCATTCCTGCTGC
         ACTTTTGCCTCTCTCTCCTG----TGCCTCTTCCATTCCTGCTGC
Line17   ACTTTTGCCTCTCTCT----------CCTCTTCCATTCCTGCTGC
         ACTTTTGCCTCTCTCTCCTG--------TCTTCCATTCCTGCTGC
         ACTTTTGCCTCTCTCTCCTGTGCTTGCCTCTTCCATTCCTGCTGC
Line18   ACTTTTGCCTCTCTCTCCTGTGCT----TCTTCCATTCCTGCTGC
         ACTTTTGCCTCTCTCTCCTGTG----------CCATTCCTGCTGC
         ACTTTTGCCTCTCTCTC------------CTTCCATTCCTGCTGC
Line19   ACTTTTGCCTCTCTCTCCTGTGCTTGCCTCTTCCATTCCTGCTGC
         ACTTTTGCCTCTCT------------------------------C
Line20   ACTTTTGCCTCTCTCTCCTGTGCTTGCCTCTTCCATTCCTGCTGC
         ACTTTTGCCTCTCTCT----------CCTCTTCCATTCCTGCTGC
         ACTTTTGCCTCTCT------------------------------C
```

Supplementary Fig. 28. Genotypes of T_0 rice lines of multiplexed large-scale genome editing at 16 target sites. PCR amplicons were sequenced using the Hi-TOM platform. PAM sequences are in red. Protospacer sequences are in blue. Substituted nucleotides are in green. Inserted nucleotides are in purple.

```
T5   WT TTGTTTGAAGAAGGGTTATGGCCAATGCTTGCCCCACATCTACCA

Line1   TTGTTTGAAGAAGGGTTATG------GCTTGCCCCACATCTACCA
        TTGTTTGAAGAAGGGTTATGGC----GCTTGCCCCACATCTACCA
Line3   TTGTTTGAAGAAGGGTTATGGC---------CCCCACATCTACCA
        TTGTTTGAAGAAGGGTTAT------TGCTTGCCCCACATCTACCA
Line4   --------48 bp deletion----------CCCACATCTACCA
        TTGTTTGAAGAAGGGTTATG----------GCCCCACATCTACCA
Line5   TTGTTTGAAGAAGGGTTATGGC----GCTTGCCCCACATCTACCA
        TTGTTTGAAGAAGGGTTATG------GCTTGCCCCACATCTACCA
Line6   TTGTTTGAAGAAGGGTTATGGC---------CCCCACATCTACCA
        TTGTTTGAAGAAGGGTTAT------TGCTTGCCCCACATCTACCA
Line7   TTGTTTGAAGAAGGGTTAT------TGCTTGCCCCACATCTACCA
        TTGTTTGAAGAAGGGTTATGGC---------CCCCACATCTACCA
Line8   TTGTTTGAAGAAGGGTTATGGC----GCTTGCCCCACATCTACCA
        TTGTTTGAAGAAGGGTTATG------GCTTGCCCCACATCTACCA
Line9   TTGTTTGAAGAAGGGTTA-----------TGCCCCACATCTACCA
        TTGTTTGAAGAAGGGTTATGGC--------GCCCCACATCTACCA
Line10  TTGTTTGAAGAAGGGTTATGGC-----CTTGCCCCACATCTACCA
        TTGTTTGAAGAAGGGTTATGGCCA-----TGCCCCACATCTACCA
Line11  TTGTTTGAAGAAGGGT------------TTGCCCCACATCTACCA
        TTGTTTGAAGAAGGGTTATGG--------TGCCCCACATCTACCA
Line12  TTGTTTGAAGAAGGGTTATGGC-----CTTGCCCCACATCTACCA
        TTGTTTGAAGAAGGGTTATGGCCAA-----GCCCCACATCTACCA
Line13  TTGTTTGAAGAAGGGTTATG----------GCCCCACATCTACCA
        TTGTTTGAAGAAGGGTTATGGC--------GCCCCACATCTACCA
Line14  TTGTTTGAAGAAGGGTTATGGC----GCTTGCCCCACATCTACCA
        TTGTTTGAAGAAGGGTTATGGC------TTGCCCCACATCTACCA
Line15  TTGTTTGAAGAAGGGTTATGGC---------CCCCACATCTACCA
        TTGTTTGAAGAAGGGTTAT------TGCTTGCCCCACATCTACCA
Line16  TTGTTTGAAGAAGGGTTATG------GCTTGCCCCACATCTACCA
        TTGTTTGAAGAAGGGTTATGGC----GCTTGCCCCACATCTACCA
Line17  TTGTTTGAAGAAGGGT------------TTGCCCCACATCTACCA
        TTGTTTGAAGAAGGGTTATGG--------TGCCCCACATCTACCA
Line18  TTGTTTGAAGAAGGGTTATG----------GCCCCACATCTACCA
        TTGTTTGAAGAAGGGTTATGG-------TTGCCCCACATCTACCA
Line19  TTGTTTGAAGAAGGGTTATGGC-------TGCCCCACATCTACCA
        TTGTTTGAAGAAGGGTTATGG-------TTGCCCCACATCTACCA
Line20  TTGTTTGAAGAAGGGTTATG------GCTTGCCCCACATCTACCA
        TTGTTTGAAGAAGGGTTATGGCCA------GCCCCACATCTACCA
```

```
T6   WT GGATTTGGGGCATGGAGACAGGAGACATAGATGGCCCGGCCATGT

Line1   GGATTTGGGGCATGGAGAC---------AGATGGCCCGGCCATGT
        GGATTTGGGGCATGGAGACA------------GGCCCGGCCATGT
Line3   GGATTTGGGGCATGGAGAC---------------CCCGGCCATGT
        GGATTTGGGGCATGGAGAC---------------CCCGGCCATGT
Line4   GGATTTGGGGCATGGAGACA----------ATGGCCCGGCCATGT
        GGATTTGGGGCATGGAG--------------------GGCCATGT
Line5   GGATTTGGGGCATGGAGAC---------AGATGGCCCGGCCATGT
        GGATTTGGGGCATGGAGACA------------GGCCCGGCCATGT
Line6   GGATTTGGGGCATGGA--------------------CGGCCATGT
        GGATTTGGGGCATGGA--------------------CGGCCATGT
Line7   GGATTTGGGGCATGGAGAC---------AGATGGCCCGGCCATGT
        GGATTTGGGGCATGGAGA-------------------GGCCATGT
Line8   GGATTTGGGGCATGGAGAC---------AGATGGCCCGGCCATGT
        GGATTTGGGGCATGGAGACA------------GGCCCGGCCATGT
Line9   GGATTTGGGGCATGGAGA--------------GGCCCGGCCATGT
        GGATTTGGGGCATGGAG---------ATAGATGGCCCGGCCATGT
        GGATTTGGGGCATGGAGACAGGAGACATAGATGGCCCGGCCATGT
Line10  GGATTTG------------------------------GGCCATGT
        GGATTTGGGGCATGGAGAC------C------GGCCCGGCCATGT
        GGATTTGGGGCATGGAGACAGG------AGATGGCCCGGCCATGT
        GGATTTGGGGCATGGAGACAGGAGACATAGATGGCCCGGCCATGT
Line11  GGAT----------------------------------------T
        GGATTTGGGGCATGGAGAC-----------ATGGCCCGGCCATGT
Line12  GGATTTGGGGCATGGAGACA------------GGCCCGGCCATGT
        GGATTTGGGGCATGGAGACAG--------GATGGCCCGGCCATGT
Line13  GGATTTGGGGCATGG-------------------CCCGGCCATGT
        GGATTTGGGGCATGGAGAC---------AGATGGCCCGGCCATGT
Line14  GGATTTGGGGCATGGAGACAG---------ATGGCCCGGCCATGT
        GGATTTGGGGCATGGAGACAG------------GCCCGGCCATGT
Line15  GGATTTGGGGCATGGAGACA-------TAGATGGCCCGGCCATGT
        GGATTTGGGGCATGGAGACAG----------------GGCCATGT
Line16  GGATTTGGGGCATGGAGAC---------AGATGGCCCGGCCATGT
        GGATTTGGGGCATGGAGACA------------GGCCCGGCCATGT
Line17  GGATTTGGGGCATGGAGACAG--------GATGGCCCGGCCATGT
        GGATTTGGGGCATG-----------------------GGCCATGT
Line18  GGATTTGGG-----------------------GGCCCGGCCATGT
        GGATTTGGGGCATGGAGAC---------AGATGGCCCGGCCATGT
        GGATTTGGG------AAGCAGGATGTTGAGC--AGATGGCCCGGC
Line19  GGATTTGGGGCATGGAGAC-----------ATGGCCCGGCCATGT
        GGATTTGGGGCATGGAGACAGG----------GGCCCGGCCATGT
Line20  GGATTTGGGGCATGGAGACAGG------AGATGGCCCGGCCATGT
        GGATTTGGGGCATGGAGACAGGAGACATAGATGGCCCGGCCATGT
```

```
T7   WT ACCTTTGGCACCATATGCTTGCTGATCAAATACTTTATTTAAGTC

Line1   ACCTTTGGCACCATATGCTT--------------TTATTTAAGTC
        ACCTTTGGCACCATATGCT-----------TACTTTATTTAAGTC
Line3   ACCTTTGGCACCATATGC-----------ATACTTTATTTAAGTC
        ACCTTTGGCACCATATGCTTG-T----------TTTATTTAAGTC
Line4   ACCTTTGGCACCATATGCTTGC--------TACTTTATTTAAGTC
        ACCTTTGGCACCATATGC------------TACTTTATTTAAGTC
Line5   ACCTTTGGCACCATATGCTT--------------TTATTTAAGTC
        ACCTTTGGCACCATATGCT-----------TACTTTATTTAAGTC
Line6   ACCTTTGGCACCATATGC-----------ATACTTTATTTAAGTC
        ACCTTTGGCACCATATGCTTG-T----------TTTATTTAAGTC
Line7   ACCTTTGGCACCATATGC-----------ATACTTTATTTAAGTC
        ACCTTTGGCACCATATGCTTG-T----------TTTATTTAAGTC
Line8   ACCTTTGGCACCATATGCTT--------------TTATTTAAGTC
        ACCTTTGGCACCATATGCT-----------TACTTTATTTAAGTC
Line9   ACCTTTGGCACCATATGCTTGC--------TACTTTATTTAAGTC
        ACCTTTGGCACCATATGCTT--------------TTATTTAAGTC
Line10  ACCTTTGGCACCATATG---------------CTTTATTTAAGTC
        ACCTTTGGCACCATATG---------------CTTTATTTAAGTC
Line11  ACCTTTGGCACCATATGCTT-----------A--TTATTTAAGTC
        ACCTTTGGCACCATATGCT-----------TACTTTATTTAAGTC
Line12  ACCTTTGGCACCATATG---------------CTTTATTTAAGTC
        ACCTTTGGCACCATATGCT-----------TACTTTATTTAAGTC
Line13  ACCTTTGGCACCATATGCTTGC--------------ATTTAAGTC
        ACCTTTGGCACCATATGCTTGC--------TACTTTATTTAAGTC
Line14  ACCTTTGGCACCATATGCTT-----------ACTTTATTTAAGTC
        ACCTTTGGCACCATATGCTT--------------TTATTTAAGTC
Line15  ACCTTTGGCACCATATGC-----------ATACTTTATTTAAGTC
        ACCTTTGGCACCATATGCTTG-T----------TTTATTTAAGTC
Line16  ACCTTTGGCACCATATGCTT--------------TTATTTAAGTC
        ACCTTTGGCACCATATGCT-----------TACTTTATTTAAGTC
Line17  ACCTTTGGCACCATATGC-------------------TTTAAGTC
        ACCTTTGGCACCATATGCT-----------TACTTTATTTAAGTC
Line18  ACCTTTGGCACCA----------------------TATTTAAGTC
        ACCTTTGGCACCAT---------------ATACTTTATTTAAGTC
Line19  ACCTTTGGCACCATATGCTTGCT-----------TTATTTAAGTC
        ACCTTTGGCACCATATGCTTGCT-------TACTTTATTTAAGTC
Line20  ACCTTTGGCACCATATG---------------CTTTATTTAAGTC
        ACCTTTGGCACCATATGCTTGCTGATCAAATACTTTATTTAAGTC
```

```
T8   WT ACCTTTAACCCTGTGTGAATGGTCAGTAAGCCCACCTACATTGAT

Line1   ACCTTTAACCCTGTGTGAATGGTCAGTAAGCCCACCTACATTGAT
        ACCTTTAACCCTGTGTGAA-------AAAACCCACCTACATTGAT
        ACCTTTAACCCTGTGTGA---------AAGCCCACCTACATTGAT
        ACCTTTAACCCTGTGTGA-------------CCACCTACATTGAT
Line3   ACCTTTAACCCTGTGTGAATGGTCAGTAAGCCCACCTACATTGAT
        ACCTTTAACCCTGTGTG----------AAGCCCACCTACATTGAT
        ACCTTTAACCCTGTGTGA---------AAGCCCACCTACATTGAT
Line4   ACCTTTAACCC----------------------ACCTACATTGAT
        ACCTTTAACCCTGTGTGAATGGTCAGTAAGCCCACCTACATTGAT
Line5   ACCTTTAACCCTGTGTGAATGGTCAGTAAGCCCACCTACATTGAT
        ACCTTTAACCCTGTGTGAATGGTCAGTAAGCCCACCTACATTGAT
Line6   ACCTTTAACCCTGTGTGAATGGTCAGTAAGCCCACCTACATTGAT
        ACCTTTAACCCTGTGTGAATGGTCAGTAAGCCCACCTACATTGAT
Line7   ACCTTTAACCCTGTGTGAATGGTCAGTAAGCCCACCTACATTGAT
        ACCTTTAACCCTGTGTGAATGGTCAGTAAGCCCACCTACATTGAT
Line8   ACCTTTAACCCTGTGTGAAT---------GCCCACCTACATTGAT
        ACCTTTAACCCTGTGTGAATGGTCAGTAAGCCCACCTACATTGAT
Line9   ACCTTTAACCCTGTGTG----------AAGCCCACCTACATTGAT
        ACCTTTAACCCTGTGTGA---------------ACCTACATTGAT
        ACCTTTAACCCTGTGTGAATGGTCAGTAAGCCCACCTACATTGAT
Line10  ACCTTTAACCCTGTGTGAATGGTCAGTAAGCCCACCTACATTGAT
        ACCTTTAACCCTGTGTGAATG----GTAAGCCCACCTACATTGAT
Line11  ACCTTTAACCCTGTGTGAATGGTCAGTAAGCCCACCTACATTGAT
        AC-------------------------------------------
Line12  ACCTTTAACCCTGTGTGAAT--------------CCTACATTGAT
        ACCTTTAACCCTGTGTGAATG----GTAAGCCCACCTACATTGAT
        ACCTTTAACCCTGTGTGAATGGTCAGTAAGCCCACCTACATTGAT
Line13  ACCTTTAACCCTGTGTGAATGGTCAGTAAGCCCACCTACATTGAT
        ACCTTTAACCCTGTGTGAATGGTCAGTAAGCCCACCTACATTGAT
Line14  ACCTTTAACCCTGTGTGA--------------------------T
        ACCTTTAACCCTGTGTGAAT-----------CCACCTACATTGAT
Line15  ACCTTTAACCCTGTGTGAATGGTCAGTAAGCCCACCTACATTGAT
        ----------------73 bp deletion---------------
Line16  ACCTTTAACCCTGTGTGAATGGTCAGTAAGCCCACCTACATTGAT
        ACCTTTAACCCTGTGTGAAT---------GCCCACCTACATTGAT
Line17  ACCTTTAACCCTGTGTGAATG----------CCACCTACATTGAT
        ACCTTTAACCCTGTGTGAATGGTCAGTAAGCCCACCTACATTGAT
Line18  ACCTTTAACCCTGTGTGAATGGTCAGTAAGCCCACCTACATTGAT
        ---------------------------------ACCTACATTGAT
Line19  ACCTTTAACCCTGTGTGAATGGTCAGTAAGCCCACCTACATTGAT
        ACCTTTAACCCTGTGTGAATGGTCAGTAAGCCCACCTACATTGAT
Line20  ACCTTTAACCCTGTGTGAATGGTCAGTAAGCCCACCTACATTGAT
        ACCTTTAACCCTGTGTGAATGGTCAGTAAGCCCACCTACATTGAT
```

Supplementary Fig. 29. Genotypes of additional T_0 rice lines of multiplexed large-scale genome editing at 16 target sites. PCR amplicons were sequenced using the Hi-TOM platform. PAM sequences are in red. Protospacer sequences are in blue. Substituted nucleotides are in green. Inserted nucleotides are in purple.

```
T9   WT CACTTTCTGTATCTCCGACACCCGGATCACGTCGTGGTAGGAGGA

Line1   CACTTTCTGTATCTCCGA---------CACGTCGTGGTAGGAGGA
        CACTTTCTGTATCTCCGAC------ATCACGTCGTGGTAGGAGGA
Line3   CACTTTCTGTATC----------------------GGTAGGAGGA
        CACTTTCTGTATCTCCGACAC------CACGTCGTGGTAGGAGGA
Line4   CACTTTCTGTATCTCCGACA-------CACGTCGTGGTAGGAGGA
        CACTTTCTGTATCTCCGACAC------CACGTCGTGGTAGGAGGA
        CACTTTCTGTATCTCCGAC----------CGTCGTGGTAGGAGGA
        CACTTTCTGTATCTCCGACA------T--CGTCGTGGTAGGAGGA
Line5   CACTTTCTGTATCTCCGACAC--------CGTCGTGGTAGGAGGA
        CACTTTCTGTATCTCCGAC------ATCACGTCGTGGTAGGAGGA
Line6   CACTTTCTGTATCTCCGACAC------CACGTCGTGGTAGGAGGA
        CACTTTCTGTATCTCCGACA-------CACGTCGTGGTAGGAGGA
        CACTT----------48 bp deletion----------------
Line7   CACTTTCTGTATCTCCGACA-------CACGTCGTGGTAGGAGGA
        CACTTTCTGTATCTCCGACAC------CACGTCGTGGTAGGAGGA
        CACTT----------48 bp deletion----------------
Line8   CACTTTCTGTATCTCCGACAC--------CGTCGTGGTAGGAGGA
        CACTTTCTGTATCTCCGAC------ATCACGTCGTGGTAGGAGGA
Line9   CACTTTCTGTATCTCCGAC----------CGTCGTGGTAGGAGGA
        ---------93 bp deletion--------TCGTGGTAGGAGGA
Line10  CACTTTCTGTATCTCCGACA----------GTCGTGGTAGGAGGA
        CACTTTCTGTATCTCCGACACC-------CGTCGTGGTAGGAGGA
Line11  CACTTTCTGTATCTCCGACAC--------CGTCGTGGTAGGAGGA
        CACTTTCTGTATCTCCGA---------CACGTCGTGGTAGGAGGA
Line12  ----------------59 bp deletion---------------
        ----------------75 bp deletion---------------
        CACTTTCTGTATCTCCGACACCC-GATCACGTCGTGGTAGGAGGA
Line13  CACTTTCTGTATCTCCGA-----G----ACGTCGTGGTAGGAGGA
        CACTTTCTGTATCTCCGACA-------CACGTCGTGGTAGGAGGA
Line14  CACTTTCT----------------------------GTAGGAGGA
        CACTTTCTGTATCTCCGACA------TCACGTCGTGGTAGGAGGA
Line15  ----------------62 bp deletion---------------
        CACTTTCTGTATCTCCG----------ACGTCGTGGTAGGAGGA
        CACTT-------------42 bp deletion-------------
        CACTTTCTGTATCTCCGAC-----------------------GGA
Line16  CACTTTCT----------------------------GTAGGAGGA
        CACTTTCTGTATCTCCGAC------ATCACGTCGTGGTAGGAGGA
Line17  CACTTTCTGTATCTCCGACAC--------CGTCGTGGTAGGAGGA
        CACTTTCTGTATCTCCGACAC-----TCACGTCGTGGTAGGAGGA
Line18  CACTTTCTGTATCTCCAGCAC-----TT---TCGTGGTAGGAGGA
        CACTTTCTGTATCTCCGACACCC------CGTCGTGGTAGGAGGA
Line19  CACTTTCTGTATCTCCGACA-------CACGTCGTGGTAGGAGGA
        CACTTTCTGTATCTCCGACA-------CACGTCGTGGTAGGAGGA
Line20  CACTTTCTGTATCTCCGACACCCGGATCACGTCGTGGTAGGAGGA
        CACTTTCTGTATCTCCGACACCCGGATCACGTCGTGGTAGGAGGA
```

```
T10  WT GACTTTGGATGATGCATCAGGTACTAGAACGCCCTCGGCACACC

Line1   GACTTTGGATGATGCAT--------------CCCTCGGCACACC
        GACTTTGGATGATGCATCAG------GAACGCCCTCGGCACACC
Line3   GACTTTGGATGATGCATCAGGT---------CCCTCGGCACACC
        GACTTTGGATGATGC--C-----C-----CGCCCTCGGCACACC
Line4   GACTTTGGATGATGCATCATGGTACTAGAACGCCCTCGGCACAC
        GACTTTGGATGATGCATC----AC-------CCCTCGGCACACC
Line5   GACTTTGGATGATGCAT------------CGCCCTCGGCACACC
        GACTTTGGATGATGCATCAG------GAACGCCCTCGGCACACC
        GACTTTGGATGATGCATCAGGTACTAGAACGCCCTCGGCACACC
Line6   GACTTTGGATGATGCATCAGGTACT--------CTCGGCACACC
        GACTTTGGATGATGCATCAGGT------ACGCCCTCGGCACACC
        GACTTTGGATGATGCATCAG----------GCCCTCGGCACACC
Line7   GACTTTGGATGATGCATCAGGTACT--------CTCGGCACACC
        GACTTTGGATGATGCATCAG------------CCTCGGCACACC
Line8   GACTTTGGATGATGCATCAG------GAACGCCCTCGGCACACC
        GACTTTGGATGATGCATC----------ACGCCCTCGGCACACC
        GACTTTGG--------------------ACGCCCTCGGCACACC
        GACTTTGGATGATGCATCAGGTACTAGAACGCCCTCGGCACACC
Line9   GACTTTGGATGATGCATC--------GAACGCCCTCGGCACACC
        GACTTTGGATGATGCATC-------------CCCTCGGCACACC
        GACTTTGGATGATGCATCAGGTACTAGAACGCCCTCGGCACACC
Line10  GACTTTGGATGATGCATCAGGTACTAGAACGCCCTCGGCACACC
        GACTTTGGATGATGCATCAGGTACTAGAACGCCCTCGGCACACC
Line11  GACTTTGGATGATGCATCAGGT------ACGCCCTCGGCACACC
        GACTTTGGATGATGCATCAGGT------ACGCCCTCGGCACACC
Line12  GACTTTGGATGATGCAT------------CGCCCTCGGCACACC
        GACTTTGGATGATGCATCAGGTACTAGAACGCCCTCGGCACACC
Line13  GACTTTGGATGATGCATCAGGT--------GCCCTCGGCACACC
        GACTTTGGATGATGCATCAGGT------ACGCCCTCGGCACACC
Line14  GACTTTGGATGATGCATCA-------------CCTCGGCACACC
        GACTTTGGATGATGCATCAG------GAACGCCCTCGGCACACC
Line15  GACTTTGGATGATGCATC-------AGAACGCCCTCGGCACACC
        GACTTTGGATGATGCATCAG------GAACGCCCTCGGCACACC
        GACTTTGGATGATGCATCAGGTACT---ACGCCCTCGGCACACC
        GACTTTGGATGATGCATCAGGT------ACGCCCTCGGCACACC
        ------43 bp deletion-----------CCCTCGGCACACC
Line16  GACTTTGGATGATGCATCA-----------GCCCTCGGCACACC
        GACTTTGGATGATGCATCAGGTACTAGAACGCCCTCGGCACACC
Line17  GACTTTGGATGATGCATCAGG-------ACGCCCTCGGCACACC
        GACTTTGGATGATGCATCAG----------GCCCTCGGCACACC
Line18  GACTTTGGATGATGCATCA-----------GCCCTCGGCACACC
        GACTTTGGATGATGCATCAGGTA--------CCCTCGGCACACC
        GACTTTGGATGATGCATCAGGTACTAGAACGCCCTCGGCACACC
Line19  GACTTTGGATGATGCATC----------ACGCCCTCGGCACACC
        GACTTTGGATGATGCATCAG------GAACGCCCTCGGCACACC
Line20  GACTTTGGATGATGCATCAGGTACTAGAACGCCCTCGGCACACC
        GACTTTGGATGATGCATCAGGTACTAGAACGCCCTCGGCACACC
```

```
T11  WT GCATTTACCGGTGAAAAGGACCTTGTCCCATCTGTGCTCCATGAG

Line1   GCATTTACCGG------------------------------TGAG
        GCATTTACCGGTGAAAAGGAC---------TCTGTGCTCCATGAG
Line3   GCATTTACCGGTGAAAAGGAC----------CTGTGCTCCATGAG
        GCATTTACCGGTGAAAAGGACC--------TCTGTGCTCCATGAG
Line4   GCATTTACCGGTGAAAAGGA--------CATCTGTGCTCCATGAG
        GCATTTACCGGTGAAAAG-A------CCCATCTGTGCTCCATGAG
Line5   GCATTTACCGG------------------------------TGAG
        GCATTTACCGGTGAAAAGGAC---------TCTGTGCTCCATGAG
Line6   GCATTTACCGGTGAAAAGGAC---------TCTGTGCTCCATGAG
        --------90 bp deletion--------TCTGTGCTCCATGAG
Line7   GCATTTACCGGTGAAAAGGAC---------TCTGTGCTCCATGAG
        GCATTTACCGGTGAAAAGGAC-TTGTCCCATCTGTGCTCCATGAG
        GCATTTACCGGTGAAAAGGACCTTGTCCCATCTGTGCTCCATGAG
Line8   GCATTTACCGG------------------------------TGAG
        GCATTTACCGGTGAAAAGGAC---------TCTGTGCTCCATGAG
Line9   GCATTTACCGGTGAAAAGGA-------CCATCTGTGCTCCATGAG
        GCATTTACCGGTGAAAAGG------------------TCCATGAG
        GCATTTACCGGTGAAAAGGACGACCTTTT----------CATGAG
        GCATTTACCGGTGAAAAGGACCTTGTCCCATCTGTGCTCCATGAG
Line10  GCATTTACCGGTGAAAAGGAC---GTCCCATCTGTGCTCCATGAG
        GCATTTACCGGTGAAAAGGACC------------------ATGAG
Line11  GCATTTACCGGTGAAAAGG---------CATCTGTGCTCCATGAG
        GCATTTACCGGTGAAAAGGA-------CCATCTGTGCTCCATGAG
Line12  GCATTTACCGGTGAAAAGGAC---GTCCCATCTGTGCTCCATGAG
        GCATTTACCGGTGAAAAGGAC---------TCTGTGCTCCATGAG
Line13  GCATTTACCGGTGAAAAGGACCTTGTCCCATCTGTGCTCCATGAG
        GCATTTACCGGTGAAAAGGA-----------CTGTGCTCCATGAG
        GCATTTACCGGTGAAAAGG-----------TCTGTGCTCCATGAG
Line14  GCATTTACCGGTGA--------------------------------G
        GCATTTACCGGTGAAAAGGAC---------TCTGTGCTCCATGAG
Line15  GCATTTACCGGTGAAAAGGACCTTGTCCCATCTGTGCTCCATGAG
        GCATTTACCGGTGAAAAGGAC---------TCTGTGCTCCATGAG
        GCATTTACCGGTGAAAAGGACC-----------GTGCTCCATGAG
Line16  GCATTTACCGG------------------------------TGAG
        GCATTTACCGGTGAAAAGGAC---------TCTGTGCTCCATGAG
Line17  GCATTTACCGGTGAAAAGG---------CATCTGTGCTCCATGAG
        GCATTTACCGGTGAAAAGG----------ATCTGTGCTCCATGAG
Line18  GCATTTACCGGTGAAAAGGA-------CCATCTGTGCTCCATGAG
        GCATTTACCGGTGAAAAGGAC-------------TGCTCCATGAG
Line19  GCATTTACCGGTGAAAAGGAC-------------TGCTCCATGAG
        GCATTTACCGGTGAAAAGGAC-----CCCATCTGTGCTCCATGAG
Line20  GCATTTACCGGTGAAAAGGA-------CCATCTGTGCTCCATGAG
        GCATTTACCGGTGAAAAGGACCTTGTCCCATCTGTGCTCCATGAG
```

```
T12  WT CCATTTCTGGGGCCTTGCAAGGTCACCTCCATAGATTACAAGGAG

Line1   CCATTTCTGGGGCCTTGCAAG-----------AGATTACAAGGAG
        CCATTTCTGGGGCCTTGCAAGG-----TCCATAGATTACAAGGAG
Line3   -----------44 bp deletion-----------TACAAGGAG
        CCATTTCTGGGGCCTTGCAAG------------GATTACAAGGAG
Line4   CCATTTCTGGGGCCTTG--------CCTCCATAGATTACAAGGAG
        CCATTTCTGGGGCCTTGCAAG----CCTCCATAGATTACAAGGAG
        -------34 bp deletion-----------AGATTACAAGGAG
Line5   CCATTTCTGGGGCCTTGCAAGG-------CATAGATTACAAGGAG
        CCATTTCTGGGGCCTTGCA-------------AGATTACAAGGAG
        CCATTTCTGGGGCCTTGCAAGGTCACCTCCATAGATTACAAGGAG
Line6   CCATTTCTGGGGCCTTG---------------------CAAGGAG
        CCATTTCTGGGGCCTTGCA-------------AGATTACAAGGAG
Line7   CCATTTC-----------------------TAGATTACAAGGAG
        CCA---C-------------------------GATTACAAGGAG
Line8   CCATTTCTGGGGCCTTG---------------------CAAGGAG
        CCATTTCTGGGGCCTTGCAAG----------TAGATTACAAGGAG
        CCATTTCTGGGGCCTTGCA-----------ATAGATTACAAGGAG
        CCATTTCTGGGGCCTTGCAA---------CATAGATTACAAGGAG
Line9   CCATTTCTGGGGCCTTGCAAGGTCACCTCCATAGATTACAAGGAG
        CC----------------------------------TTACAAGGAG
        CCATTTCTGGGGCCTTGCAAG------------GATTACAAGGAG
Line10  ----------------59 bp deletion---------------
        CCATTTCTGGGGCCTTGCA-----------ATAGATTACAAGGAG
        CCATTTCTGGGGCCTTGCAA----------ATAGATTACAAGGAG
Line11  CCATTTCTGGGGCC---------------------TTACAAGGAG
        CCATTTCTGGGGCCTTGCAAGGTCACCTCCATAGATTACAAGGAG
Line12  CCATTTCTGGGGCCTTGCA-----------ATAGATTACAAGGAG
        CCATTTCTGGGGCCTTG------------CATAGATTACAAGGAG
Line13  CCATTTCTGGGGCCTTG------------CATAGATTACAAGGAG
        CCATTTCTGGGGCCTTGCA-----------ATAGATTACAAGGAG
Line14  CCATTTCTGGGGCCTTGCAA-----------TAGATTACAAGGAG
        CCATTTCTGGGGCCTTGC---------------GATTACAAGGAG
Line15  CCATTTCTGGGGCCTTG-----------CCATAGATTACAAGGAG
        CCATTTCTGGGGCCTTGCA-----------ATAGATTACAAGGAG
        CCATTTCTGGGGCCTTGCAAG---------ATAGATTACAAGGAG
Line16  CCATTTCTGGGGCCTTGCA-----------ATAGATTACAAGGAG
        CCATTTCTGGGGCCTTGC----------------ATTACAAGGAG
        CCATTTCTGGGGCCTTGCAA---------CATAGATTACAAGGAG
Line17  CCATTTCTGGGGCCTTGCAAGGTCACCTCG--AGATTACAAGGAG
        CCATTTCTGGGGCCTTGCAAGGTCACCTCCATAGATTACAAGGAG
Line18  CCATTTCTGGGGCCTTGCAA----A-------AGATTACAAGGAG
        CCATTTCTGGGGCCTTGCAAGGAGGTG---ATAGATTACAAGGAG
Line19  CCATTTCTGGGGCCTTG---------------------CAAGGAG
        CCATTTCTGGGGCCTTGCAAG----------TAGATTACAAGGAG
Line20  CCATTTCTGGGGCCTTGCAAGGTCACCTCCATAGATTACAAGGAG
        CCATTTCTGGGGCCTTGCAAGGTCACCTCCATAGATTACAAGGAG
```

Supplementary Fig. 30. Genotypes of additional T_0 rice lines of multiplexed large-scale genome editing at 16 target sites. PCR amplicons were sequenced using the Hi-TOM platform. PAM sequences are in red. Protospacer sequences are in blue. Substituted nucleotides are in green. Inserted nucleotides are in purple.

```
T13  WT AAATTTGCCGGCAGCTAATAGGGATCTAAACACACTAGTCATATC

 Line1  AAATTTGCCGGCAGCTAATAG-------AACACACTAGTCATATC
        AAATTTGCCGGCAGCTAAT-----------CACACTAGTCATATC
        AAATTTGCCGGCAGCTAATA-------AAACACACTAGTCATATC
 Line3  AAATTTGCCGGCAGCT-------------ACACACTAGTCATATC
        AAATTTGCCGGCAGCTAATAG---------CACACTAGTCATATC
 Line4  AAATTTGCCGGCAGCT----------------CACTAGTCATATC
        AAATTTGCCGGCAGCTAATAGGG---------CACTAGTCATATC
 Line5  AAATTTGCCGGCAGCTAATAG--------ACACACTAGTCATATC
        AAATTTGCCGGCAGCTAATAG-------AACACACTAGTCATATC
 Line6  AAAT---------------------------ACACTAGTCATATC
        AAATTTGCCGGCAGCTAATAG--------ACACACTAGTCATATC
 Line7  AAAT---------------------------ACACTAGTCATATC
        AAATTTGCCGGCAGCTAATAG--------ACACACTAGTCATATC
 Line8  AAATTTGCCGGCAGCTAATAG--------ACACACTAGTCATATC
        AAATTTGCCGGCAGCTAATAG-------AACACACTAGTCATATC
 Line9  AAATTTGCCGGCAGCTAATAG--A-------ACACTAGTCATATC
        AAATTTGCCGGCAGCTAAT----------ACACACTAGTCATATC
 Line10 AAATTTGCCGGCAGCT-------------ACACACTAGTCATATC
        AAATTTGCCGGCAGCTAATAGGG------ACACACTAGTCATATC
        AAATTTGCCGGCAGCT------------AACACACTAGTCATATC
        AAATTTGCCGG----------40 bp deletion----------
 Line11 AAATTTGCCGGCAGCT-------------ACACACTAGTCATATC
        AAATTTGCCGGCAGCTAATAG--------ACACACTAGTCATATC
 Line12 AAATTTGCCGGCAGCTAAT------------ACACTAGTCATATC
        AAATTTGCCGGCAGCTAAT---------AACACACTAGTCATATC
 Line13 AAATTTGCCGGCAGCTAATAG---------CACACTAGTCATATC
        AAATTTGCCGGCAGCTAATAG---T-----CACACTAGTCATATC
 Line14 AAATTTGCCGGCAGCTAATAGG-------ACACACTAGTCATATC
        AAATTTGCCGGCAGCTAA--------TAAACACACTAGTCATATC
 Line15 AAAT---------------------------ACACTAGTCATATC
        AAATTTGCCGGCAGCTGAAGTGAGGTGACCTGGTGAAGGACATAT
 Line16 AAATTTGCCGGCAGCTAATAG--------ACACACTAGTCATATC
        AAATTTGCCGGCAGCTAATAG-------AACACACTAGTCATATC
 Line17 AAATTTGCCGGCAGCTAAT---------AACACACTAGTCATATC
        AAATTTGCCGGCAGCTAAT-----T----ACACACTAGTCATATC
        AAATTTGCCGGCAGCTAATAGGG------ACACACTAGTCATATC
 Line18 AAATTTGCCGGCAG-----------------ACACTAGTCATATC
        AAATTTGCCGGCAGCTAATA-----CTAAACACACTAGTCATATC
 Line19 AAATTTGCCGGCAGCT------------AACACACTAGTCATATC
        AAATTTGCCGGCAGCTAAT---------AACACACTAGTCATATC
 Line20 AAATTTGCCGGCAGCTAATAGGGATCTAAACACACTAGTCATATC
        AAATTTGCCGGCAGCTAATAGGGATCTAAACACACTAGTCATATC
```

```
T14  WT TGCTTTATACGTGGAAACAATGACAGTTCACACAGGAGGAGGTTG

 Line1  TGCTTTATACGTGGAAACAA----------------------TTG
        TGCTTTATACGTGGAAACAAT-----------CAGGAGGAGGTTG
 Line3  TGCTTTATACGTGGAAACAAT-------------GGAGGAGGTTG
        TGCTTTATACGTGGAAACAAT----------ACAGGAGGAGGTTG
 Line4  TGCTTTATACGTGGAAA-----------CACACAGGAGGAGGTTG
        TGCTTTAT---------------------ACACAGGAGGAGGTTG
 Line5  TGCTTTATACGTGGAAACAA----------------------TTG
        TGCTTTATACGTGGAAACAAT-----------CAGGAGGAGGTTG
 Line6  TGCTTTATACGTGGAAACAAT-------------GGAGGAGGTTG
        TGCTTTATACGTGGAAACAAT----------ACAGGAGGAGGTTG
 Line7  TGCTTTATACGTGGAAACAAT----------ACAGGAGGAGGTTG
        TGCTTTATACGTGGAAACAAT-------------GGAGGAGGTTG
 Line8  TGCTTTATACGTGGAAACAA----------------------TTG
        TGCTTTATACGTGGAAACAAT-----------CAGGAGGAGGTTG
 Line9  TGCTTTATACGTGGAAA-----------CACACAGGAGGAGGTTG
        TGCTTTATACGTGGAAACAAT--------ACACAGGAGGAGGTTG
 Line10 TGCTTTATACGTGGAAAC-------41 bp deletion------
        TGCTTTATACGTGGAAACAAT--------ACACAGGAGGAGGTTG
 Line11 TGCTTTATACGTGGAAACAAATG----30 bp deletion----
        TGCTTTATACGTGGAAACAATG--------CACAGGAGGAGGTTG
 Line12 TGCTTTATACGTGGAAAC-------41 bp deletion------
        TGCTTTATACGTGGAAACAAT--------ACACAGGAGGAGGTTG
 Line13 TGCTTTATACGTGGAAA-----------CACACAGGAGGAGGTTG
        TGCTTTAT---------------------ACACAGGAGGAGGTTG
 Line14 TGCTTTATACGTGGAAACAAT-----------CAGGAGGAGGTTG
        TGCTTTATACGTGGAAACAAT----------------------TG
 Line15 TGCTTTATACGTGGAAACAAT-------------GGAGGAGGTTG
        TGCTTTATACGTGGAAACAAT----------ACAGGAGGAGGTTG
 Line16 TGCTTTATACGTGGAAACAA----------------------TTG
        TGCTTTATACGTGGAAACAAT-----------CAGGAGGAGGTTG
 Line17 TGCTTTATACGTGGAAACAATG--------CACAGGAGGAGGTTG
        TGCTTTATACGTGGAAACAATG--------CACAGGAGGAGGTTG
 Line18 TGCTTTATACGTGGAAACA---AC------------AGGAGGTTG
        TGCTTTATACGTGGAAACA----------ACACAGGAGGAGGTTG
 Line19 TGCTTTATACGTGGAAACAAT---------CACAGGAGGAGGTTG
        TGCTTTATACGTGGAAACAAT-----------CAGGAGGAGGTTG
 Line20 TGCTTTATACGTGGAAA-----------CACACAGGAGGAGGTTG
        TGCTTTATACGTGGAAACAATG-----TCACACAGGAGGAGGTTG
```

```
T15  WT CTCTTTCTCCTGAGGAGCAAGAGCCATCACAGGTAAGAAAGAACT

 Line1  CTCTTTCTCCTGAGGAGCAAG-G---T--CAGGTAAGAAAGAACT
        CTCTTTCTCCTGAGGAGCAAGAG-------AGGTAAGAAAGAACT
 Line3  CTCTTTCTCCTGAGGAG------------------AGAAAGAACT
        CTCTTTCTCCTGAGGAGCAAG----ATCACAGGTAAGAAAGAACT
 Line4  CTCTTTCTCCTGAGGAGC------------------------ACT
        CTCTTTCTCCTGAGGAGCAAG-------ACAGGTAAGAAAGAACT
 Line5  CTCTTTCTCCTGAGGAGCAAG-G---T--CAGGTAAGAAAGAACT
        CTCTTTCTCCTGAGGAGCAAGAG-------AGGTAAGAAAGAACT
 Line6  CTCTTTCTCCTGAGGAG------------------AGAAAGAACT
        CTCTTTCTCCTGAGGAGCAAG----ATCACAGGTAAGAAAGAACT
 Line7  CTCTTTCTCCTGAGGAG------------------AGAAAGAACT
        CTCTTTCTCCTGAGGAGCAAG----ATCACAGGTAAGAAAGAACT
 Line8  CTCTTTCTCCTGAGGAGCAAGAG-------AGGTAAGAAAGAACT
        CTCTTTCTCCTGAGGAGCAAG-G---T--CAGGTAAGAAAGAACT
 Line9  CTCTTTCTCCTGAG-----------------------------CT
        CTCTTTCTCCTGAGGAGCAA----------AGGTAAGAAAGAACT
 Line10 CTCTTTCTCCTGAGGAG-------CATCACAGGTAAGAAAGAACT
        CTCTTTCTCCTGAGGAGCAAG-------ACAGGTAAGAAAGAACT
 Line11 CTCTTTCTCCTGAGGAGCAAG-G------CAGGTAAGAAAGAACT
        CTCTTTCTCCTGAGGAGCAAGA----------GTAAGAAAGAACT
 Line12 CTCTTTCTCCTGAGGAGCAA-----ATCACAGGTAAGAAAGAACT
        CTCTTTCTCCTGAGGAGCAA-----ATCACAGGTAAGAAAGAACT
 Line13 CTCTTTCTCCTGAGGAGCA-----------AGGTAAGAAAGAACT
        CTCTTTCTCCTGAGGAGCAAG-------ACAGGTAAGAAAGAACT
 Line14 CTCTTTCTCCTGAGGAGCAAGA----------------AAGAACT
        CTCTTTCTCCTGAGGAGCAAG-G---T--CAGGTAAGAAAGAACT
 Line15 CTCTTTCTCCTGAGGAG------------------AGAAAGAACT
        CTCTTTCTCCTGAGGAGCAAG----ATCACAGGTAAGAAAGAACT
 Line16 CTCTTTCTCCTGAGGAGCAAG-G---T--CAGGTAAGAAAGAACT
        CTCTTTCTCCTGAGGAGCAAGAG-------AGGTAAGAAAGAACT
 Line17 CTCTTTCTCCTGAGGAGCAAG-G------CAGGTAAGAAAGAACT
        CTCTTTCTCCTGAGGAG---------T--------AGAAAGAACT
 Line18 CTCTTTCTCCTGAGGAGCAAG-----T--CAGGTAAGAAAGAACT
        CTCTTTCTCCTGAGGAGCAAG----ATCACAGGTAAGAAAGAACT
 Line19 CTCTTTCTCCTGAGGAGCAAGA--------------------ACT
        CTCTTTCTCCTGAGGAGCAA------------GTAAGAAAGAACT
 Line20 CTCTTTCTCCTGAGGAGCAAG--------CAGGTAAGAAAGAACT
        CTCTTTCTCCTGAGGAGCAAGAGAGCCATCACAGGTAAGAAAGAA
```

```
T16  WT ATGTTTGAGCATATGGTTGTAACTTCAGAAAAACTGAATCCCCAA

 Line1  ATGT----------------------------------------A
        ATGTTTGAGCATATGGTTGT---------AAAACTGAATCCCCAA
 Line3  ---------36 bp deletion---------ACTGAATCCCCAA
        ---------36 bp deletion---------ACTGAATCCCCAA
 Line4  ATGTTTGAGCATATGG------30 bp deletion---------
        ATGTTTGAGCATATGGTT------------AAACTGAATCCCCAA
 Line5  ATGTTTGAGCATATGGTT------------AAACTGAATCCCCAA
        ATGTTTGAGCATATGGTTGT-----------AACTGAATCCCCAA
 Line6  ATGTTTGAGCATATGGTT---------------CTGAATCCCCAA
        ATGTTTGAGCATATGGTTGTAA---CAGAAAAACTGAATCCCCAA
 Line7  ATGTTTGAGCATATGGTT---------------CTGAATCCCCAA
        ATGTTTGAGCATATGGTTG----------AAAACTGAATCCCCAA
 Line8  ATGTTTGAGCATATGGTTGT----------AAACTGAATCCCCAA
        ATGTTTGAGCATATGGTTGT----------AAACTGAATCCCCAA
 Line9  ATGTTTGAGCATATGGTTGT-----------AACTGAATCCCCAA
        ATGTTTGAGCATATGGTTGT--------AAAAACTGAATCCCCAA
 Line10 ATGTTTGAG---------------------AAACTGAATCCCCAA
        ATGTTTGAGCATATGGTTGT----------AAACTGAATCCCCAA
        ATGTT----------------------------CTGAATCCCCAA
 Line11 ATG--------------------------------------CCAA
        ATGTTTGAGCATATGGTTGT-----------AACTGAATCCCCAA
 Line12 ATGTTTGAGCATATGGTTGT----------AAACTGAATCCCCAA
        ATGTTTGAG---------------------AAACTGAATCCCCAA
 Line13 ATGTTTGAGCATATGGTTGT---------AAAACTGAATCCCCAA
        ATGTTTGAGCATATGGTTGT--------AAAAACTGAATCCCCAA
 Line14 ATGTTTGAGCATATGGTTGTAA----------ACTGAATCCCCAA
        ATGTTTGAGCATATGGTTGTAA----------ACTGAATCCCCAA
 Line15 ATGTTTGAGCATATGGTT---------------CTGAATCCCCAA
        ATGTTTGAGCATATGGTTGT---------AAAACTGAATCCCCAA
 Line16 ATGTTTGAGCATATGGTTGT----------AAACTGAATCCCCAA
        ATGTTTGAGCATATGGTTGT----------AAACTGAATCCCCAA
 Line17 ATG--------------------------------------CCAA
        ATGTTTGAGCATATGGTTGT-----------AACTGAATCCCCAA
 Line18 ATGTTTGAGCATATGGTTGTAA---CAGAAAAACTGAATCCCCAA
        ATGTTTGAGCATATGGTTGTAA---CAGAAAAACTGAATCCCCAA
 Line19 ATGTTTGAGCATATGGTTG------------AACTGAATCCCCAA
        ATGTTTGAGCATATGGTTG-------------------TCCCCAA
 Line20 ATGTTTGAGCATATGGTTG------------AACTGAATCCCCAA
        ATGTTTGAGCATATGGTTG------------AACTGAATCCCCAA
```

Supplementary Fig. 31. Genotypes of additional T_0 rice lines of multiplexed large-scale genome editing at 16 target sites. PCR amplicons were sequenced using the Hi-TOM platform. PAM sequences are in red. Protospacer sequences are in blue. Substituted nucleotides are in green. Inserted nucleotides are in purple.

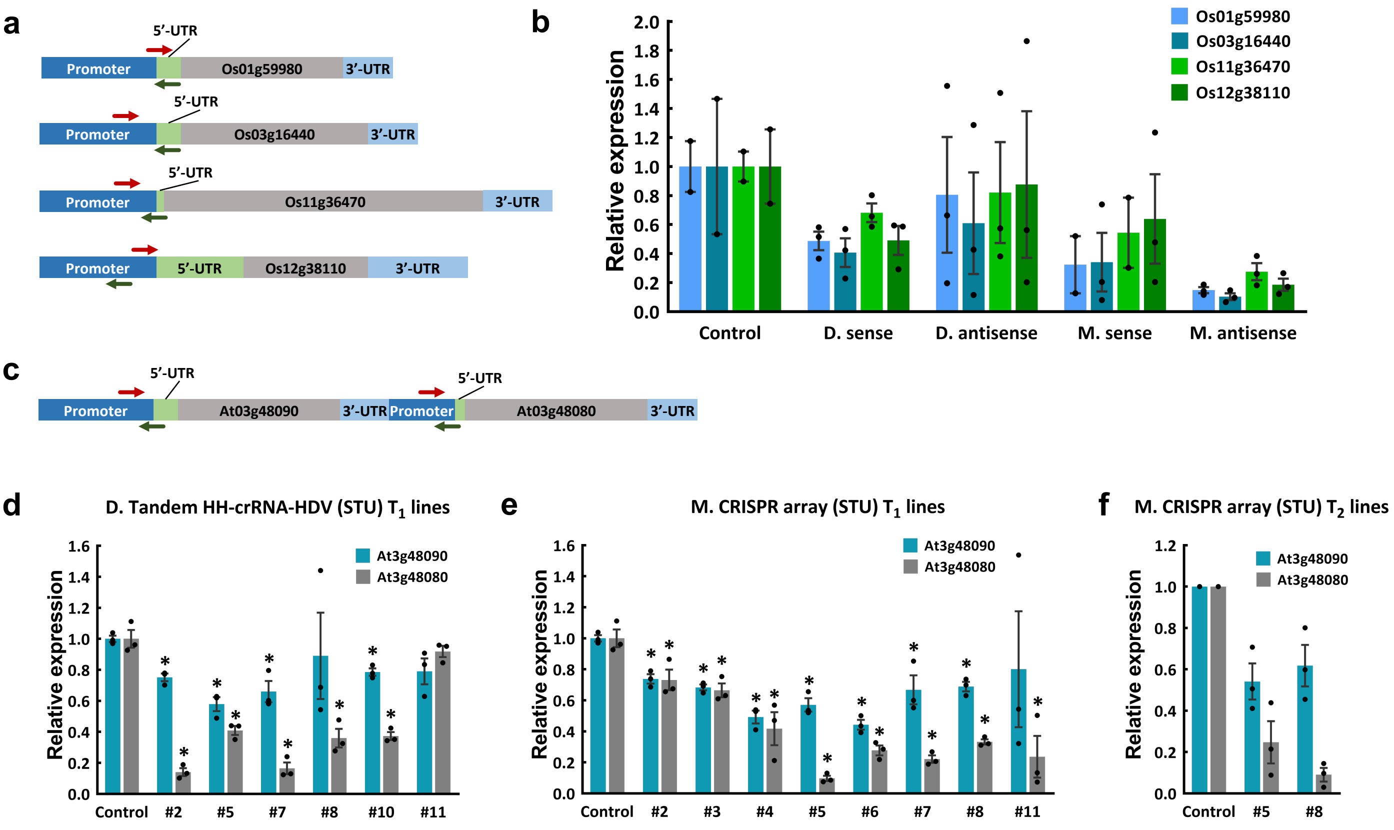

At3g48090	0.00149	0.00105	0.00882	0.71366	0.00245	0.07100
At3g48080	0.00016	0.00076	0.00025	0.00151	0.00055	0.29380

At3g48090	0.00199	0.00034	0.00039	0.00083	0.00012	0.02558	0.00110	0.62216
At3g48080	0.03752	0.00978	0.00845	0.00011	0.00037	0.00023	0.00035	0.00636

Supplementary Fig. 32. Multiplexed transcriptional repression with two compacted STU dCas12a-SRDX systems. a, Schematics of the rice target genes and crRNAs. Red arrows indicate crRNAs targeting the antisense strand of DNA. Green arrows indicate crRNAs targeting the sense strand of DNA. **b**, Simultaneous transcriptional repression of four genes in rice protoplasts by two STU systems, D and M. **c**, Schematics of the *Arabidopsis* target genes and crRNAs. Red arrows indicate crRNAs targeting the antisense strand of DNA. Green arrows indicate crRNAs targeting the sense strand of DNA. **d**, Simultaneous transcriptional repression of two tandemly arrayed genes in *Arabidopsis* T_1 lines by the STU system D. **e**, Simultaneous transcriptional repression in *Arabidopsis* T_1 lines by the STU system M. **f,** Targeted transcriptional repression is inherited to the T_2 generation. T_2 lines are the progenies of T_1 lines with the same line number. Transcription levels of target genes were quantified by qRT-PCR. Data are presented as mean values ± SEM. n=3 biological replicates in "**b**" and "**f**", and n=3 technical replicates in "**d**" and "**e**". Asterisks indicate significant differences (p<0.05) between CRISPR lines and the control line using two-sided Student's t-test. *P* values are shown under each line in "**d**" and "**e**". Source data are provided as a Source Data file.

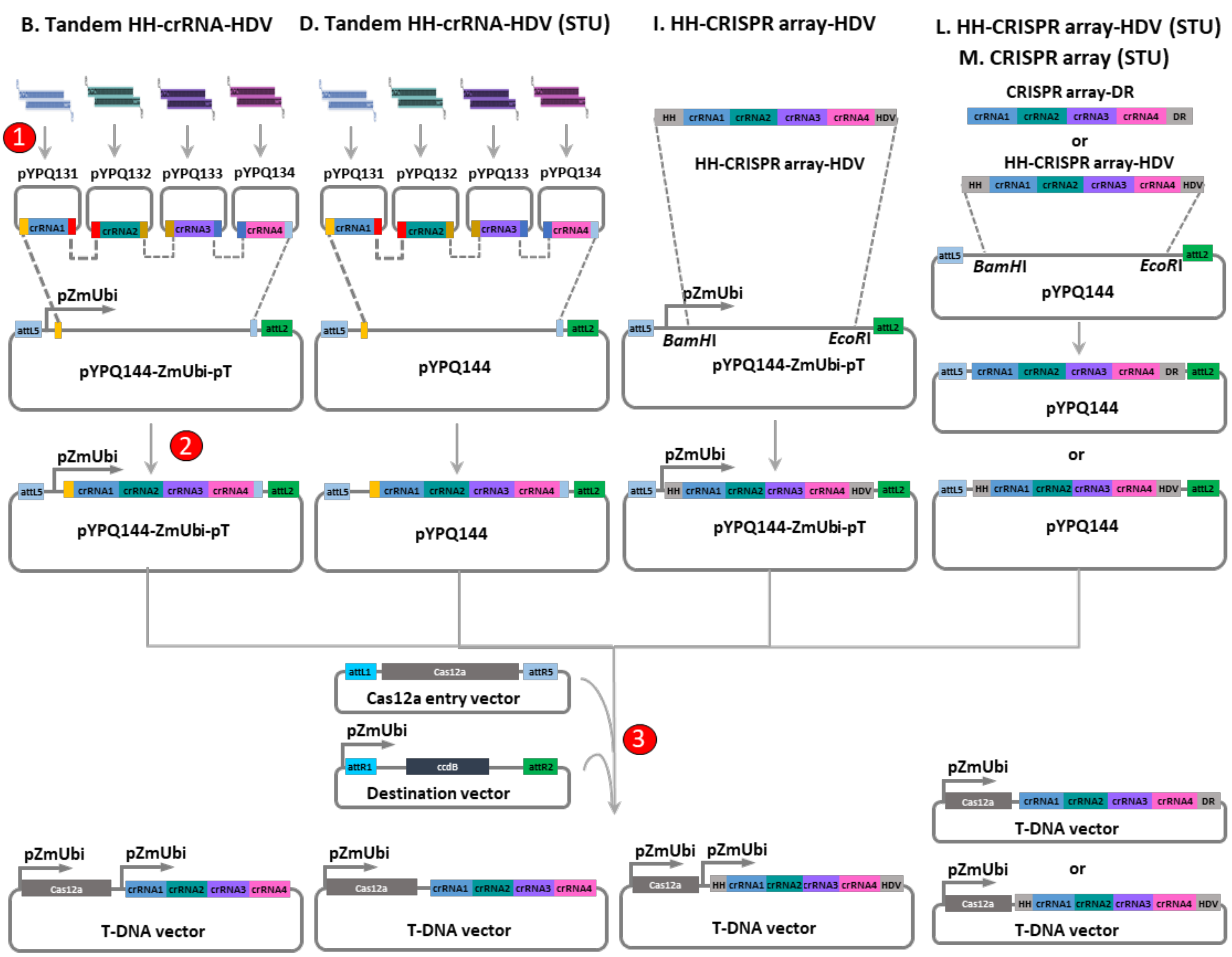

Supplementary Fig. 33. Modular assembly systems for multiplexed plant genome engineering. Assembly of multiplexed Cas12a systems follows a streamlined modular approach. In step 1, the protospacers for crRNAs are cloned into crRNA entry clones for HH-crRNA-HDV based systems. CRISPR arrays can be directly synthesized for crRNA array-based systems. In step 2, assembly of multiple crRNA cassettes is achieved either through Golden Gate cloning (for HH-crRNA-HDV systems) or conventional cloning at *BamH*I and *EcoR*I sites (for crRNA array systems), resulting attL5-attL2 crRNA expression vectors. In step 3, final T-DNA expression vectors are assembled by three-way Gateway recombination reactions.

Supplementary Table 1. Multiplexed CRISPR-Cas9 genome editing systems

Study	Species	Cas9	Cas9 promoter	sgRNA promoter	Targets	Multiplexing strategy	Editing efficiency	Biallelic editing efficiency	Efficiency of biallelic editing of all targets
**Lowder et al., 2015[1]**	Rice	pcoCas9	35S	U6/3	3	Tandem U6/3-sgRNA-Terminator	33.3-53.3%	20.0-40.0%	NA
**Xing et al., 2014[2]**	Maize	zCas9	Ubi1	U3	2	Tandem U3-sgRNA-Terminator	NA	At least 60%	NA
	Arabidopsis	zCas9	35S	U6	3 (2 sgRNAs)	Tandem U6-sgRNA-Terminator	42-100% (T1)	NA	NA
**Vazquez-Vilar et al., 2016[3]**	N. benthamiana	hCas9	35S	U6	2	Tandem U6-sgRNA-Terminator	9.9-12.1%	Agro-infiltration	NA
**Zhang et al., 2016[4]**	Arabidopsis	hCas9	AtUBQ1	U6, U3b, 7SL	6	Tandem Pol III Promoter-sgRNA-Terminator	13-93% (T1)	NA	NA
**Ma et al., 2015[5]**	Rice	pcoCas9	35S	U6/3	1,2,3,8,	Tandem U6/3-sgRNA-Terminator	0-100% (average 85.4%)	0-100% (average 79.6%)	0[a] (8 targets) 15/24 (highest for 3 targets) All (highest for 2 targets, limited plants sampled)
	Arabidopsis	pcoCas9	35S	U6/3	1,3	Tandem U6/3-sgRNA-Terminator	22.2-60% (average 35.6%)	0-33.3% (average 8.5%)	0[b] (3 targets)
**Tang et al., 2016[6]**	Rice	Cas9	ZmUbi	-	2	Hammerhead ribozyme (RZ) cleavage sites-sgRNA-cleavage site-sgRNA-cleavage site-RZ (STU)	66.7-93.1%	22.2-89.7%	NA
**Xie et al., 2015[7]**	Rice	Cas9	OsUBI10	U3	2,4,8	Tandem tRNA-sgRNA array	47-100%	6-86%	6/17 (2 targets) 1/17 (2 targets tested in 4-targets multiplex) 7/14 (5 targets tested in 8-targets multiplex)
**Ding et al., 2018[8]**	Rice	Cas9	OsUBI10	-	2,4	Tandem tRNA-sgRNA array in intron (STU)	50-85%	0-73%	NA
**Qi et al., 2016[9]**	Maize	zCas9	ZmUbi	U6	2,4	Tandem tRNA (maize glycine)-sgRNA array	85.7-100% (calculated for each gene with 2 or 4 sgRNA)	NA	NA
**Wang et al., 2018[10]**	Rice	Cas9	ZmUbi	U6	3	Tandem U6-sgRNA	70.8-79.2%	37.5-66.7%	7/24
	Rice	Cas9	ZmUbi	-	3	sgRNA linked by 6bp linker (STU)	50.0-86.4%	18.2-68.2%	0[c]

**Wang et al., 2016**[11]	Wheat	wheat codon optimized Cas9		U6	3	Tandem tRNA-sgRNA array	0.5-7.4%	NA	NA
**Tang et al., 2018**[12]	Rice	Cas9	ZmUbi	-	2,3	Hammerhead ribozyme (RZ) cleavage sites-sgRNA-cleavage site-sgRNA-cleavage site-RZ (STU)	58.3-87.2%	33.3-71.8%	26/39 (2 targets) 11/36 (3 targets)
	Rice	Cas9	ZmUbi	-	2,3	sgRNAs flanked and linked by Csy4 recognition sites (STU)	60.6-93.9%	39.4-84.4%	24/32 (2 targets) 13/33 (3 targets)
	Rice	Cas9	ZmUbi	-	2,3,6	Tandem tRNA-sgRNA array with 1 tRNA at the end (STU)	60.5-97.4%	18.4-90.0%	22/30 (2 targets) 23/37 (3 targets) 4/38 (6 targets)

[a] 3 out of 3 lines have 7 targets biallelically edited.

[b] The best performing line has 2 targets biallelically edited, 1 target no edit.

[c] The best performing lines (2 lines out of 22 lines) have 2 targets biallelically edited (including homozygous), 1 target heterozygous. The possibility to get 3 targets biallelically edited in T_1 generation is 1/4.

Supplementary Table 2. Multiplexed CRISPR-Cas12a genome editing systems

Study	Cas12a	Cas12a promoter	crRNA promoter	Targets	Multiplexing strategy	Editing efficiency	Biallelic editing efficiency	Efficiency of biallelic editing of all genes
Wang et al., 2017[13]	FnCas12a	ZmUbi	U6	4	CRISPR array	43.8-75%	6.3-28.1%	1/32 (only 1 plant shown)
	LbCas12a	ZmUbi	U6	4	CRISPR array	40-60%	10-20%	1/30 (only 1 plant shown)
Wang et al., 2018[10]	FnCas12a	ZmUbi	ZmUbi	8	HH-CRISPR array-HDV	0-70.8%	0-66.7%	0[a]
	FnCas12a	ZmUbi	-	8	CRISPR array-DR (STU)	0-70.8%	0-41.7%	0[b]
	LbCas12a	ZmUbi	CmYLCV	9	tRNA- CRISPR array-DR-tRNA	4.2-54.2%	0-50%	0[c]
	LbCas12a	ZmUbi	-	9	tRNA- CRISPR array-DR-tRNA (STU)	4.2-70.8%	0-41.7%	0[d]
Tang et al., 2019[12]	LbCas12a	ZmUbi	-	4	CRISPR array-DR (STU)	29.2-50%	4.2-33.3%	1/24
Hu et al., 2019[14]	FnCas12a	OsACTIN1	U3	4	CRISPR array	34.2-45%	2.2-43.5%	NA
	FnCas12a	OsACTIN1	U3	4	Truncated tRNA-crRNA array	29.2-55.6%	3.2-19.4%	NA
This study	LbCas12a	ZmUbi	ZmUbi	4	Tandem HH-crRNA-HDV	100%	72.2-100%	26/36[e]
	LbCas12a	ZmUbi	-	4	Tandem HH-crRNA-HDV (STU)	60-100%	8.3-95%	3/60
	LbCas12a	ZmUbi	-	4	HH-CRISPR array-HDV (STU)	52.8-100%	13.9-88.9%	2/36
	LbCas12a	ZmUbi	-	4	CRISPR array-DR (STU)	56.7-93.3%	23.3-80%	3/30
	LbCas12a	ZmUbi	ZmUbi	4	HH-CRISPR array-HDV	18.2-90.9%	12.1-45.5%	1/33
	LbCas12a	ZmUbi	U6	4	CRISPR array	30-82%	10-60%	4/50
	LbCas12a	ZmUBi	ZmUbi	4	Tandem tRNA-HH-crRNA-HDV	5.6-63.9%	0-16.7%	0

[a] The best performing line (only this one line out of 24 lines) has 6 targets biallelically edited, 1 target heterozygous, one target no edit. The possibility to get 7 targets biallelically edited in T_1 generation is ¼.

[b] The best performing line (only this one line out of 24 lines) has 3 targets biallelically edited, 3 targets heterozygous, 1 target chimeric, 1 target no edit. The possibility to get 6 targets biallelically edited in T_1 generation is 1/64.

[c] The best performing line (only this one line out of 24 lines) has 4 targets biallelically edited, 2 targets heterozygous, 2 targets chimeric, 1 target no edit. The possibility to get 6 targets biallelically edited in T_1 generation is 1/16.

[d] The best performing line (only this one line out of 24 lines) has 4 targets biallelically edited (including homozygous), 4 targets heterozygous, 1 target no edit. The possibility to get 8 targets biallelically edited in T_1 generation is 1/256.

[e] The multiplexing strategy highlighted in red is the best performing multiplexing system B identified in this study.

Supplementary Table 3. Off-target analysis of multiplexed genome editing by Mb2Cas12a

Supplementary Table 3a. Off-target analysis of multiplexed genome editing by Mb2Cas12a targeting four sites

Target gene	Target site with PAM	Off-target site[1]	Chromosome	Start	End	Mismatches	Bulge Size	Off-target edits	
								Line10	Line11
OsPDS	TTTGGTGGTGTACAGTAGGGGGAGATG	TTAGTatTGTACAGTAGGGtGGATG	chr4	4907304	4907329	3	1	No	No
		TTTGTGcaGTACAGTAGGcGGGATG	chr5	22862184	22862209	3	1	No	No
		TTAGgGGTGTAAGTAaGGGGAGAgG	chr3	28881350	28881375	3	1	No	No
		TTAGTtGTtTACAAGTAGGGtGAGATG	chr12	24872563	24872590	3	1	No	No
OsDEP1	TTTGCTCCTATATAAAGCACCACAACT	TTCCTCTATATAAAGCcCCACAgCc	chr5	13199576	13199601	3	1	No	No
		TTTCcCaTATcTAAAGCACCACAAT	chr11	28136076	28136101	3	1	No	No
		TTTCcCaTATcTAAAGCACCACAAT	chr11	28186639	28186664	3	1	No	No
		TTTtCCTAaATAAAcCACCACAACT	chr4	29393361	29393386	3	1	No	No
		TTTCTCCTATATAAtGgACCACAGACa	chr1	18394707	18394734	3	1	No	No
OsROC5	TTTCCCTCTCTCTCCTGTGCTTGCCTC	TTTCCTCTCTCTtCTtTGCcTGCCTa	chr9	5396740	5396766	4	0	No	No
		TTACCTCTtcCTCCTcTtCTTGCCTC	chr12	7725512	7725538	4	0	No	No
		TTTaCTCTCTCTCCcGTGCaTGCCgC	chr12	8612122	8612148	4	0	No	No
		TTTaCTCTCTCTCCcGTGCaTGCCgC	chr12	8620241	8620267	4	0	No	No
		TTTaCTCTCTCTCCcGTGCaTGCCgC	chr5	9406550	9406576	4	0	No	No
		TTTaCTCTCTCTCCcGTGCaTGCCgC	chr5	9415106	9415132	4	0	No	No
		TTTaCTCTCTCTCCcGTGCaTGCCgC	chr5	11281498	11281524	4	0	No	No
		TTTaCTCTCTCTCCcGTGCaTGCCgC	chr5	11505695	11505721	4	0	No	No
		TTTaCTCTCTCTCCcGTGCaTGCCgC	chr5	11517355	11517381	4	0	No	No
		TTTCCTCTCTCTCCcGTGCacGCCgC	chr5	10307460	10307486	4	0	No	No
		TTAtCTCcCTCTgCTtTGCTTGCCTC	chr6	16867148	16867174	4	0	No	No
		TTACCTCTCTCTCCTcTcCTctCCTC	chr2	16997014	16997040	4	0	No	No
		TTTaCTCTCTCTCCcGTGCaTGCCgC	chr11	18215076	18215102	4	0	No	No
		TTTaCTCTCTCTCCcGTGCaTGCCgC	chr11	18223631	18223657	4	0	No	No
		TTGCCTCTCTCcCCgGTGCTccCCTC	chr7	22719091	22719117	4	0	No	No
		TTTgCTCTCTCTCgTtTGgTTGCCTC	chr4	24355528	24355554	4	0	No	No
		TTTCCTCTCTCTCCaGTGCTCTGCCgC	chr7	3896630	3896657	2	1	No	No
OsmiR528	TTTGTAGGGTTGACGAATTCATCGGAA	TTTTAGGGTTGAGgtTTCATCaGAA	chr1	18524408	18524433	3	1	No	No

[1] Mismatched nucleotides are in lower case.

Supplementary Table 3b. Off-target analysis of multiplexed genome editing by Mb2Cas12a targeting six sites for quantitative traits

Target gene	Target site with PAM	Off-target site[1]	Chromosome	Start	End	Mismatches	Bulge Size	Off-target edits	
								Line1	Line2
**OsSWEET11**	TTTGNGAGTGAAATCTCTTGTCTTAAGG	TTAGATGAAATCTaTTGcCTcAAGG	chr9	18098793	18098818	3	1	No	No
**OsSWEET13**	TTTGCACCACGATACTCCACAGCATAA	TTACACACatTgCTCCACAGCATAA	chr3	17294307	17294332	3	1	No	No
**OsSWEET14**	TTTGCTACTGTTGCAAGTGCTCACCCA	TTGCTACTGTGCAgGTtCTCAgCCA	chr1	3463032	3463057	3	1	No	No
		TTGCTACTGTTGgAATcCTCACCgA	chr5	4921797	4921822	3	1	No	No
		TTACTACTGTTGCAtGTGCCACtCt	chr1	31407391	31407416	3	1	No	No
		TTGCaACTGTTGCAAGTGACTgAtCCA	chr6	28368038	28368065	3	1	No	No
**OsGS3**	TTTGCTTGAAGGCTTGATGAGCTTAGC	TTCCTTGAtGGCTTGgTGAtCTcAGC	chr3	4484179	4484205	4	0	No	No
		TTCCTTGGGtTTGATGAGgTTAGC	chr4	23124666	23124690	2	2	No	No
		TTGCTTGAgGtCTTGATGAGCTgAGa	chr3	30394017	30394043	4	0	No	No

[1] Mismatched nucleotides are in lower case.

Supplementary Table 4. Vectors for Cas12a genome editing

Supplementary Table 4a. Gateway compatible vectors for assembly of Cas12a ortholog expression systems for plant genome editing

Vector type	Vector name (Addgene #)	Reference
crRNA entry vector	pYPQ141-ZmUbi-RZ-Lb (#86197)	[1]
	pYPQ141-ZmUbi-RZ-Fn (#108864)	[2]
Cas12a entry vector	pYPQ230 (Lb; #86210)	[1]
	pYPQ239 (Fn; #108859)	[2]
	pYPQ281 (Mb; #138113); pYPQ282 (Ts; #138114); pYPQ283 (Ml; #138115); pYPQ284 (Mb2; #138116); pYPQ284-RVR(#138117); pYPQ284-RVRR (#160327); pYPQ284-v1 (#138118); pYPQ284-v2 (#138119); pYPQ285 (Lb5; #138120); pYPQ285-RVR (#138121); pYPQ286 (CMa; #138122); pYPQ287 (Bs; #138123); pYPQ287-RVR (#138124); pYPQ288 (Bo; #138125); pYPQ289 (Er; #138126); pYPQ289-RVR (#138127)	This study
Destination vector	pYPQ203 (#86207)	[1]

Supplementary Table 4b. Golden Gate and Gateway compatible vectors for assembly of top-performing multiplex Cas12a systems for plant genome editing and transcriptional repression

Vector type	Vector name (Addgene #)	Reference
Golden gate assembly vector	pYPQ131-STU-Lb (#138096); pYPQ132-STU-Lb (#138099); pYPQ133-STU-Lb (#138102); pYPQ134-STU-Lb (#138105); pYPQ131-STU-As (#138094); pYPQ132-STU-As (#138097); pYPQ133-STU-As (#138100); pYPQ134-STU-As (#138103); pYPQ131-STU-Fn (#138095); pYPQ132-STU-Fn (#138098); pYPQ133-STU-Fn (#138101); pYPQ134-STU-Fn (#138104);	This study
Recipient vector	pYPQ142 (#69294); pYPQ143 (#69295); pYPQ144 (#69296)	[1]
	pYPQ142-ZmUbi (#138106); pYPQ143-ZmUbi (#138107); pYPQ144-ZmUbi-pT (#138108)	This study
Cas12a entry vector	pYPQ230 (Lb editing; #86210); pYPQ220 (As editing; #86208) pYPQ233 (Lb repression; #86211); pYPQ223 (As repression; #86209)	[15]
	pYPQ239 (Fn editing; #108859)	[16]
	pYPQ230-STU (Lb editing; #138110); pYPQ239-STU (Fn editing; #138112); pYPQ233-STU (Lb repression; #138111); pYPQ223-STU (As repression; #138109)	This study
Destination vector	pYPQ202 (#86198); pYPQ203 (#86207)	[15]

References

1. Lowder, L. G. *et al.* A CRISPR/Cas9 toolbox for multiplexed plant genome editing and transcriptional regulation. *Plant Physiol.* **169**, 971–985 (2015).
2. Xing, H.-L. *et al.* A CRISPR/Cas9 toolkit for multiplex genome editing in plants. *BMC Plant Biol* **14**, (2014).
3. Vazquez-Vilar, M. *et al.* A modular toolbox for gRNA–Cas9 genome engineering in plants based on the GoldenBraid standard. *Plant Methods* **12**, 10 (2016).
4. Zhang, Z. *et al.* A multiplex CRISPR/Cas9 platform for fast and efficient editing of multiple genes in Arabidopsis. *Plant Cell Rep.* **35**, 1519–1533 (2016).
5. Ma, X. *et al.* A robust CRISPR/Cas9 system for convenient, high-efficiency multiplex genome editing in monocot and dicot plants. *Molecular Plant* **8**, 1274–1284 (2015).
6. Tang, X. *et al.* A single transcript CRISPR-Cas9 system for efficient genome editing in plants. *Molecular Plant* **9**, 1088–1091 (2016).
7. Xie, K., Minkenberg, B. & Yang, Y. Boosting CRISPR/Cas9 multiplex editing capability with the endogenous tRNA-processing system. *PNAS* **112**, 3570–3575 (2015).
8. Ding, D., Chen, K., Chen, Y., Li, H. & Xie, K. Engineering introns to express RNA guides for Cas9- and Cpf1-mediated multiplex genome editing. *Molecular Plant* **11**, 542–552 (2018).
9. Qi, W. *et al.* High-efficiency CRISPR/Cas9 multiplex gene editing using the glycine tRNA-processing system-based strategy in maize. *BMC Biotechnology* **16**, 58 (2016).
10. Wang, M. *et al.* Multiplex gene editing in rice with simplified CRISPR-Cpf1 and CRISPR-Cas9 systems: Simplified single transcriptional unit CRISPR systems. *Journal of Integrative Plant Biology* **60**, 626–631 (2018).
11. Wang, W., Akhunova, A., Chao, S. & Akhunov, E. Optimizing multiplex CRISPR/Cas9-based genome editing for wheat. *bioRxiv* 051342 (2016) doi:10.1101/051342.
12. Tang, X. *et al.* Single transcript unit CRISPR 2.0 systems for robust Cas9 and Cas12a mediated plant genome editing. *Plant Biotechnology Journal* **17**, 1431–1445 (2019).
13. Wang, M., Mao, Y., Lu, Y., Tao, X. & Zhu, J. Multiplex gene editing in rice using the CRISPR-Cpf1 system. *Molecular Plant* **10**, 1011–1013 (2017).
14. Hu, X., Meng, X., Li, J., Wang, K. & Yu, H. Improving the efficiency of the CRISPR-Cas12a system with tRNA-crRNA arrays. *The Crop Journal* **8**, 403–4407 (2020).
15. Tang, X. et al. A CRISPR–Cpf1 system for efficient genome editing and transcriptional repression in plants. *Nature Plants* **3**, 17103 (2017).
16. Zhong, Z. et al. Plant genome editing using FnCpf1 and LbCpf1 nucleases at redefined and altered PAM sites. *Molecular Plant* **11**, 999–1002 (2018).
