# Supplementary material for: Expanding the scope of plant genome engineering with Cas12a orthologs and highly multiplexable editing systems
Source: Nat Commun. 2021 Mar 29;12:1944. doi: 10.1038/s41467-021-22330-w (PMC8007695; doi:10.1038/s41467-021-22330-w)
Supplement: Supplementary file 2 — Description of Additional Supplementary Files [file 41467_2021_22330_MOESM2_ESM.pdf]

Supplementary Data 1
Description: Genome editing efficiencies of Cas12a in rice protoplasts

Supplementary Data 2
Description: Primers and gBlocks used in this study

Supplementary Data 3
Description: T-DNA vectors used in this study
