# Supplementary material for: Expanding the scope of plant genome engineering with Cas12a orthologs and highly multiplexable editing systems
Source: Nat Commun. 2021 Mar 29;12:1944. doi: 10.1038/s41467-021-22330-w (PMC8007695; doi:10.1038/s41467-021-22330-w)
Supplement: Supplementary file 6 — Reporting Summary [file 41467_2021_22330_MOESM6_ESM.pdf]

nature research

Corresponding author(s): Yiping Qi, Yong Zhang

Last updated by author(s): 02/15/2021

Reporting Summary

Nature Research wishes to improve the reproducibility of the work that we publish. This form provides structure for consistency and transparency in reporting. For further information on Nature Research policies, see Authors & Referees and the Editorial Policy Checklist .

Statistics

For all statistical analyses, confirm that the following items are present in the figure legend, table legend, main text, or Methods section.

n/a	Confirmed	
☐	☒	The exact sample size (*n*) for each experimental group/condition, given as a discrete number and unit of measurement
☒	☐	A statement on whether measurements were taken from distinct samples or whether the same sample was measured repeatedly
☐	☒	The statistical test(s) used AND whether they are one- or two-sided<br>*Only common tests should be described solely by name; describe more complex techniques in the Methods section.*
☐	☒	A description of all covariates tested
☒	☐	A description of any assumptions or corrections, such as tests of normality and adjustment for multiple comparisons
☐	☒	A full description of the statistical parameters including central tendency (e.g. means) or other basic estimates (e.g. regression coefficient) AND variation (e.g. standard deviation) or associated estimates of uncertainty (e.g. confidence intervals)
☐	☒	For null hypothesis testing, the test statistic (e.g. *F*, *t*, *r*) with confidence intervals, effect sizes, degrees of freedom and *P* value noted<br>*Give P values as exact values whenever suitable.*
☒	☐	For Bayesian analysis, information on the choice of priors and Markov chain Monte Carlo settings
☒	☐	For hierarchical and complex designs, identification of the appropriate level for tests and full reporting of outcomes
☒	☐	Estimates of effect sizes (e.g. Cohen's *d*, Pearson's *r*), indicating how they were calculated

Our web collection on statistics for biologists contains articles on many of the points above.

Software and code

Policy information about availability of computer code

Data collection	No software was used.
Data analysis	SnapGene 4.3.10 was used to analyze and construct DNA vectors. ImageJ bundled with Java 1.8.0_172 and Bio-Rad Image Lab™ 6.1 were used to analyze DNA electrophoresis results. DSDecodeM was used to decode Sanger sequencing data for revealing genome edits. CRISPRMatch, CRISPResso2 and Hi-TOM online tool were used to analyze and quantify genome editing results based on deep sequencing of PCR amplicons. Perl 5.30.3 was used for PAM analysis.

For manuscripts utilizing custom algorithms or software that are central to the research but not yet described in published literature, software must be made available to editors/reviewers. We strongly encourage code deposition in a community repository (e.g. GitHub). See the Nature Research guidelines for submitting code & software for further information.

Data

Policy information about availability of data

All manuscripts must include a data availability statement. This statement should provide the following information, where applicable:

- Accession codes, unique identifiers, or web links for publicly available datasets
- A list of figures that have associated raw data
- A description of any restrictions on data availability

Accession codes. Next-generation sequencing (NGS) data have been deposited to the National Center for Biotechnology information (NCBI) database under Sequence Read Archive (SRA) BioProject ID: PRJNA595844. The 35 Golden Gate and Gateway compatible vectors for the new Cas12a toolbox are available at Addgene (Supplementary Table 1).

Field-specific reporting

Please select the one below that is the best fit for your research. If you are not sure, read the appropriate sections before making your selection.

- [x] Life sciences
- [ ] Behavioural & social sciences
- [ ] Ecological, evolutionary & environmental sciences

For a reference copy of the document with all sections, see nature.com/documents/nr-reporting-summary-flat.pdf

Life sciences study design

All studies must disclose on these points even when the disclosure is negative.

Sample size	For assays in protoplasts, according to the common practice, three biological replicates were used for each sample/treatment. Specifically, 360 µL rice protoplasts (2x1000,000 cells/mL) were used for PEG-mediated transfection per sample. For assays in stable transgenic plants, the numbers of rice T0 plants varied among different experiments, ranging from 12 to 60. This is based on a rationale that a minimal of 12 individual plants are sufficient for calculating editing efficiency in each T0 population. For assays in stable transgenic Arabidopsis plants, three independent T1 plants were used for each construct.
Data exclusions	No data were excluded from analyses.
Replication	For rice protoplasts assays, three biological replicates were used for each construct. The biological replicates were done at intervals ranging from days to weeks between experiments and performed by up to two different researchers. All findings have been replicated independently at least once in the investigators' labs.
Randomization	Rice protoplasts were isolated and randomly separated to each transformation. The constructs used for transfection didn't need to follow any specific order.
Blinding	The investigators were blinded to the grouping of Cas12a orthologs in the initial testing in rice protoplasts. For the remaining experiments with stable transgenic rice and Arabidopsis lines, blinding is not applicable as no bias will be introduced by knowing the identity of each sample. All samples were processed identically through standard procedures (e.g., DNA transformation, DNA isolation, and DNA sequencing).

Reporting for specific materials, systems and methods

We require information from authors about some types of materials, experimental systems and methods used in many studies. Here, indicate whether each material, system or method listed is relevant to your study. If you are not sure if a list item applies to your research, read the appropriate section before selecting a response.

Materials & experimental systems

n/a	Involved in the study	
☒	☐	Antibodies
☒	☐	Eukaryotic cell lines
☒	☐	Palaeontology
☒	☐	Animals and other organisms
☒	☐	Human research participants
☒	☐	Clinical data

Methods

n/a	Involved in the study	
☒	☐	ChIP-seq
☒	☐	Flow cytometry
☒	☐	MRI-based neuroimaging
