# Supplementary material for: Expanding the scope of plant genome engineering with Cas12a orthologs and highly multiplexable editing systems
Source: Nat Commun. 2021 Mar 29;12:1944. doi: 10.1038/s41467-021-22330-w (PMC8007695; doi:10.1038/s41467-021-22330-w)
Supplement: Supplementary file 7 — Source Data [file 41467_2021_22330_MOESM7_ESM.zip › Source Data/Source Data-20210302.docx]

**Source Data Files**

**Expanding the scope of plant genome engineering with Cas12a orthologs and highly multiplexable editing systems**

Yingxiao Zhang^1,Ɨ^, Qiurong Ren^2,Ɨ^, Xu Tang^2,Ɨ^, Shishi Liu^2^, Aimee A. Malzahn^1^, Jianping Zhou^2^, Jiaheng Wang^2^, Desuo Yin^1,3^, Changtian Pan^1^, Mingzhu Yuan^2^, Lan Huang^2^, Han Yang^2^, Yuxin Zhao^2^, Qing Fang^2^, Xuelian Zheng^2^, Li Tian^2^, Yanhao Cheng^1,4^, Ysa Le^1^, Bailey McCoy^1^, Lidiya Franklin^1^, Jeremy D. Selengut^5^, Stephen M. Mount^6^, Qiudeng Que^7^, Yong Zhang^2*^, Yiping Qi^1,8*^

^1^Department of Plant Science and Landscape Architecture, University of Maryland, College Park, Maryland 20742, USA;

^2^Department of Biotechnology, School of Life Science and Technology, Center for Informational Biology, University of Electronic Science and Technology of China, Chengdu 610054, China;

^3^Food Crop Institute, Hubei Academy of Agricultural Sciences, Wuhan, Hubei 430064, China;

^4^College of Agriculture, Nanjing Agricultural University, Nanjing, Jiangsu 210095, China;

^5^Center for Bioinformatics and Computational Biology, University of Maryland, College Park, Maryland 20742, USA

^6^Department of Cell Biology and Molecular Genetics, University of Maryland, College Park, Maryland 20742, USA;

^7^Syngenta, Research Triangle Park, North Carolina 27709, USA;

^8^Institute for Bioscience and Biotechnology Research, University of Maryland, Rockville, Maryland 20850, USA.

^Ɨ^These authors contributed equally to this work.

***Corresponding authors:** Yiping Qi, Email: [yiping@umd.edu](mailto:yiping@umd.edu); Yong Zhang, Email: [zhangyong916@uestc.edu.cn](mailto:zhangyong916@uestc.edu.cn)


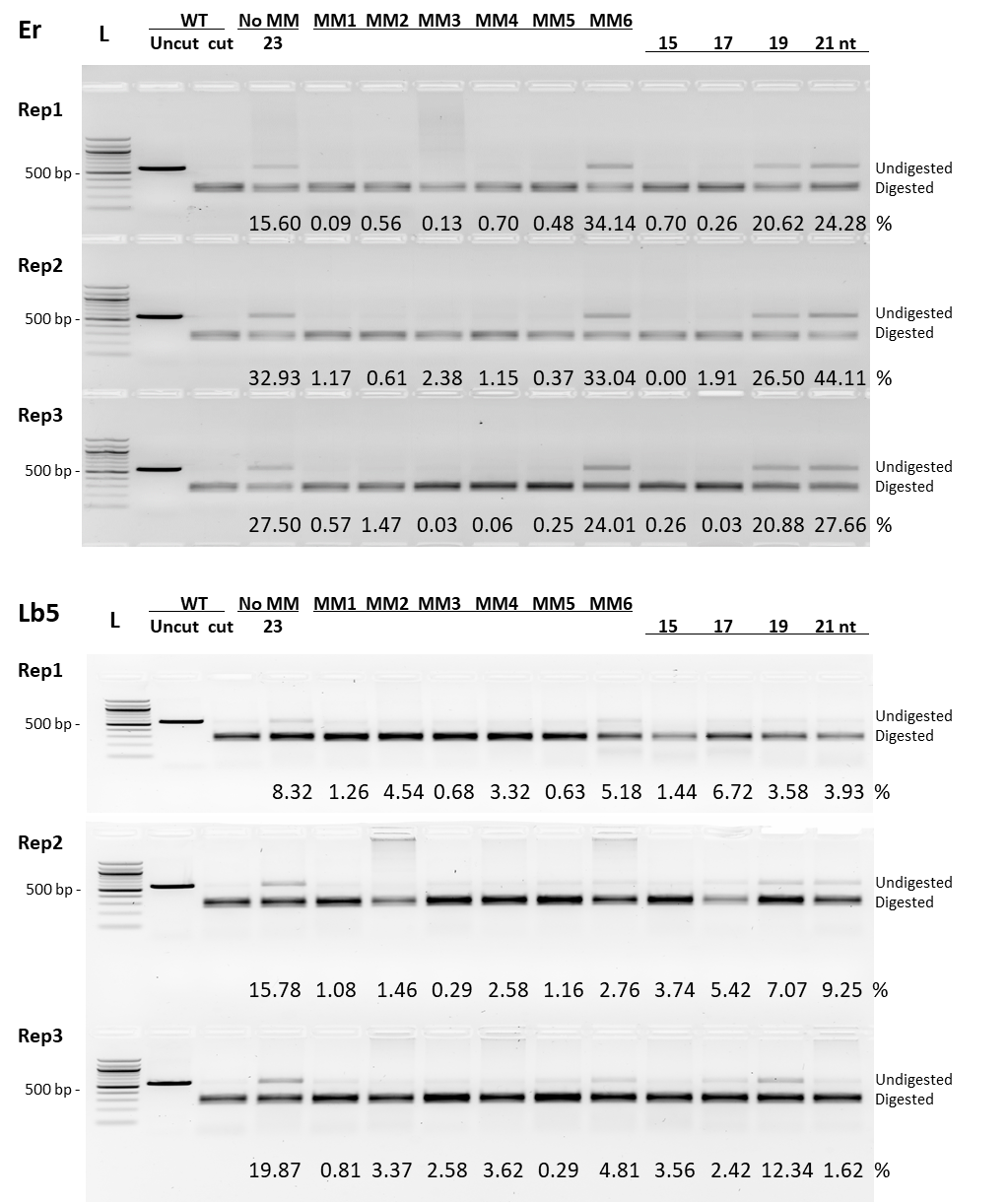
Panel 1. Original gel images for Figure 1c and 1d, Supplementary Figure 4. Agarose gels were stained using ethidium bromide and visualized using the Bio-Rad ChemiDoc Imaging system. L, NEB 100 bp DNA Ladder. WT, wild type rice protoplast. Uncut, PCR amplicon of target sites before restriction enzyme digestion. Cut, PCR amplicon of target sites after restriction enzyme digestion. MM, mismatch. Protospacer length (nt) is indicated by numbers. Editing efficiencies (%) shown under each lane were calculated by subtracting the editing efficiencies of WT. Each experiment includes three biological replicates.

Panel 1 (Continued). Original gel images for Figure 1c and 1d, Supplementary Figure 4. Agarose gels were stained using ethidium bromide and visualized using the Bio-Rad ChemiDoc Imaging System. L, NEB 100 bp DNA Ladder. WT, wild type rice protoplast. Uncut, PCR amplicon of target sites before restriction enzyme digestion. Cut, PCR amplicon of target sites after restriction enzyme digestion. MM, mismatch. Protospacer length (nt) is indicated by numbers. Editing efficiencies (%) shown under each lane were calculated by subtracting the editing efficiencies of WT. Each experiment includes three biological replicates.


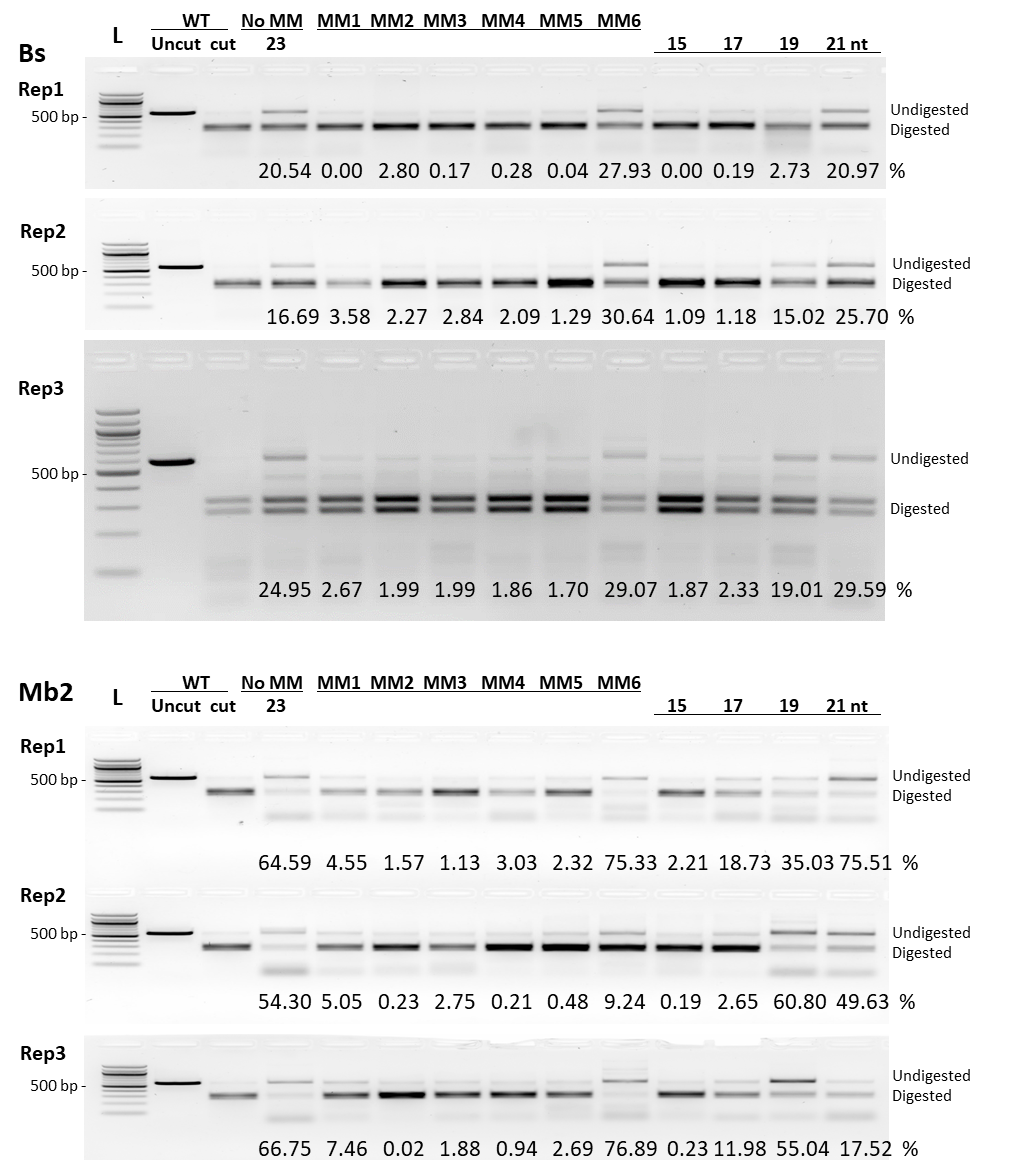


Panel 2. Original gel images for Figure 2b, Supplementary Figure 8. Agarose gels were stained using ethidium bromide and visualized using the Bio-Rad ChemiDoc Imaging System. L, NEB 100 bp DNA Ladder. WT, wild type rice protoplast. Uncut, PCR amplicon of target sites before restriction enzyme digestion. Cut, PCR amplicon of target sites after restriction enzyme digestion. Asterisks indicate non-specific amplifications. Editing efficiencies (%) shown under each lane were calculated by subtracting the editing efficiencies of WT. Each experiment includes three biological replicates.


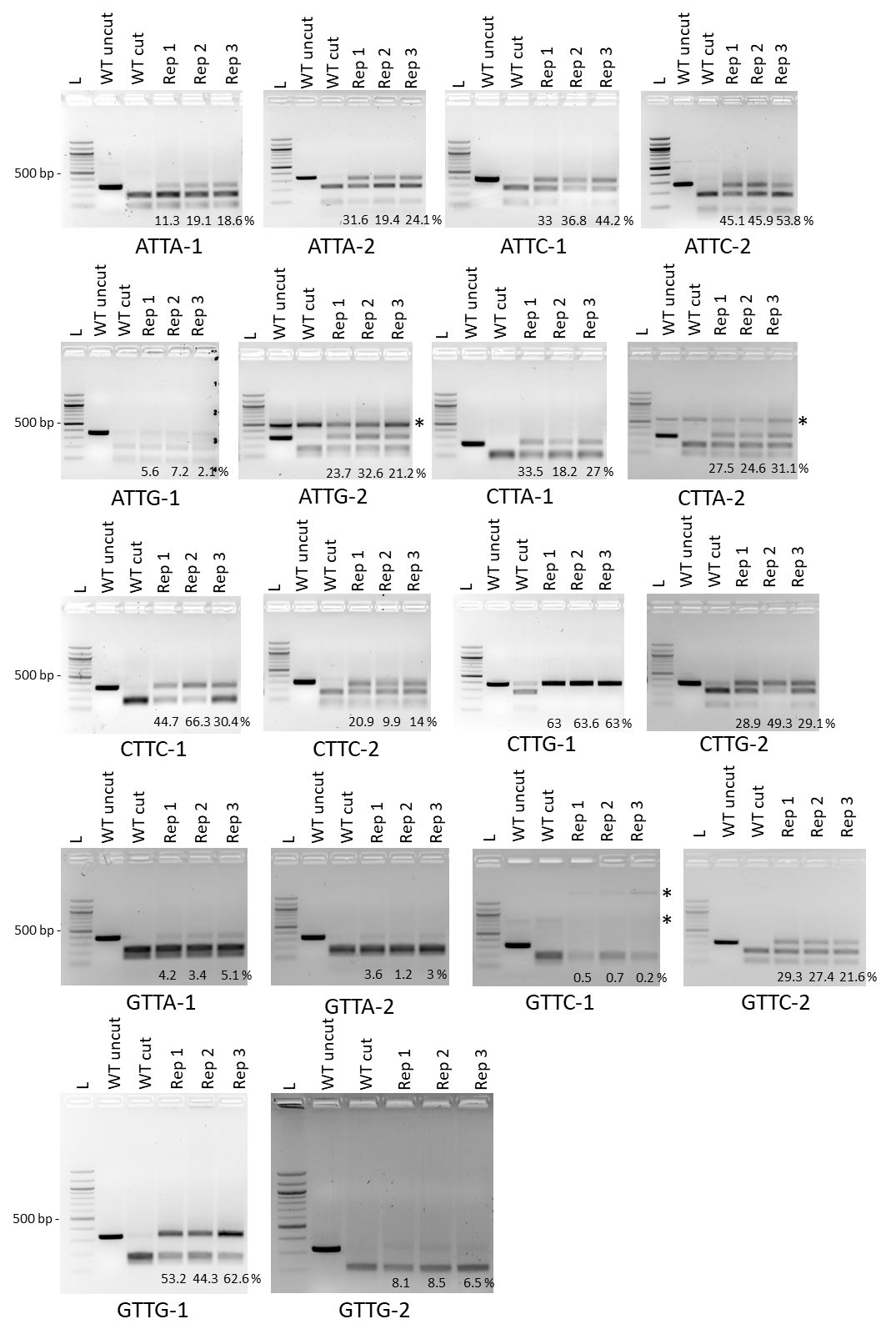


Panel 3. Original gel images for Figure 2d, Supplementary Figure 10. Agarose gels were stained using ethidium bromide and visualized using the Bio-Rad ChemiDoc Imaging System. L, NEB 100 bp DNA Ladder. WT, wild type rice protoplast. Uncut, PCR amplicon of target sites before restriction enzyme digestion. Cut, PCR amplicon of target sites after restriction enzyme digestion. The asterisk indicates non-specific amplifications. Editing efficiencies (%) shown under each lane were calculated by subtracting the editing efficiencies of WT. Each experiment includes three biological replicates.


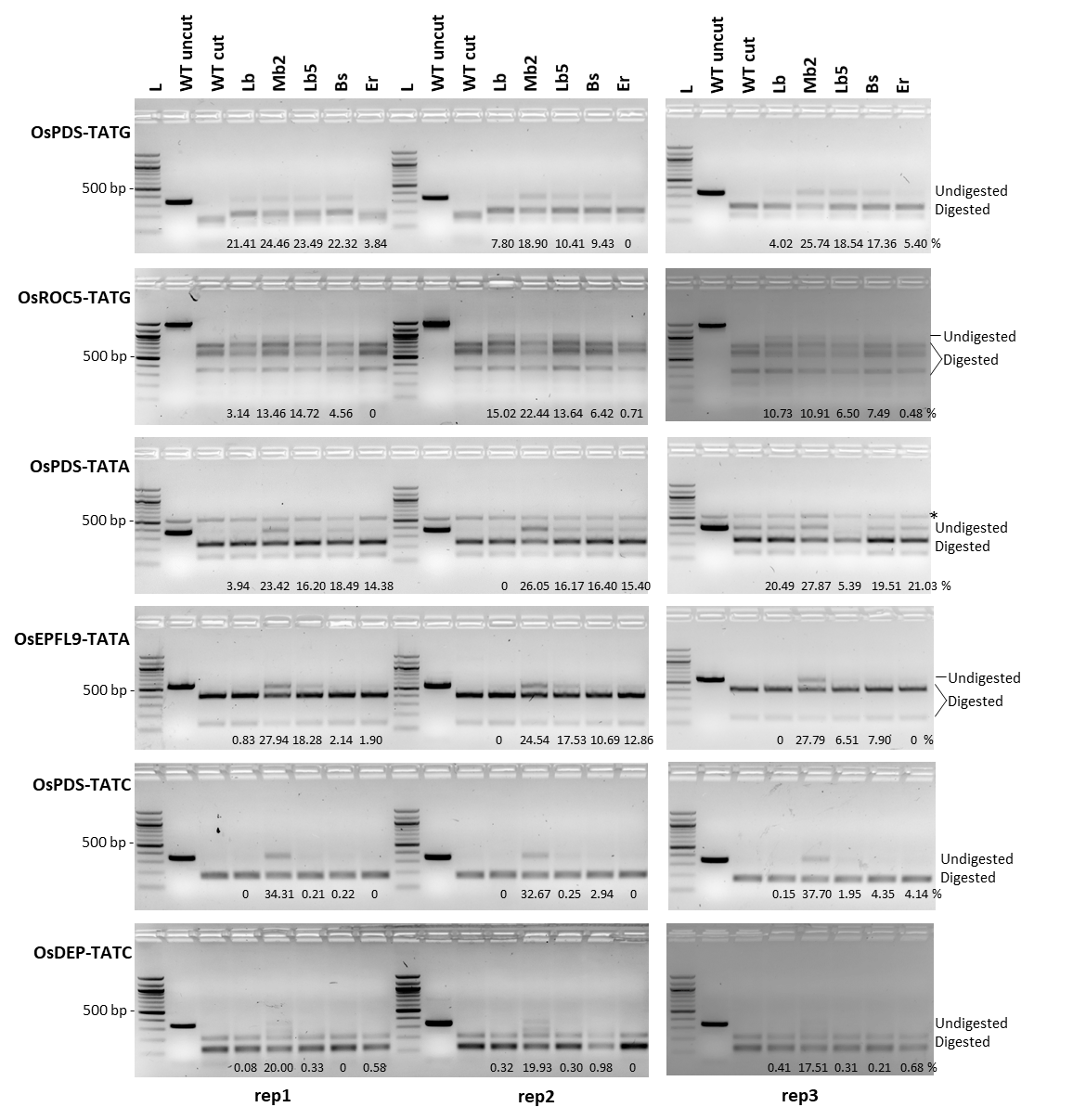


Panel 4. Original gel images for Figure 2e. Agarose gels were stained using ethidium bromide and visualized using the Bio-Rad ChemiDoc Imaging System. L, NEB 100 bp DNA Ladder. WT, wild type rice protoplast. Uncut, PCR amplicon of target sites before restriction enzyme digestion. Cut, PCR amplicon of target sites after restriction enzyme digestion. Asterisks indicate non-specific amplifications. Editing efficiencies (%) shown under each lane were calculated by subtracting the editing efficiencies of WT. Each experiment includes three biological replicates.


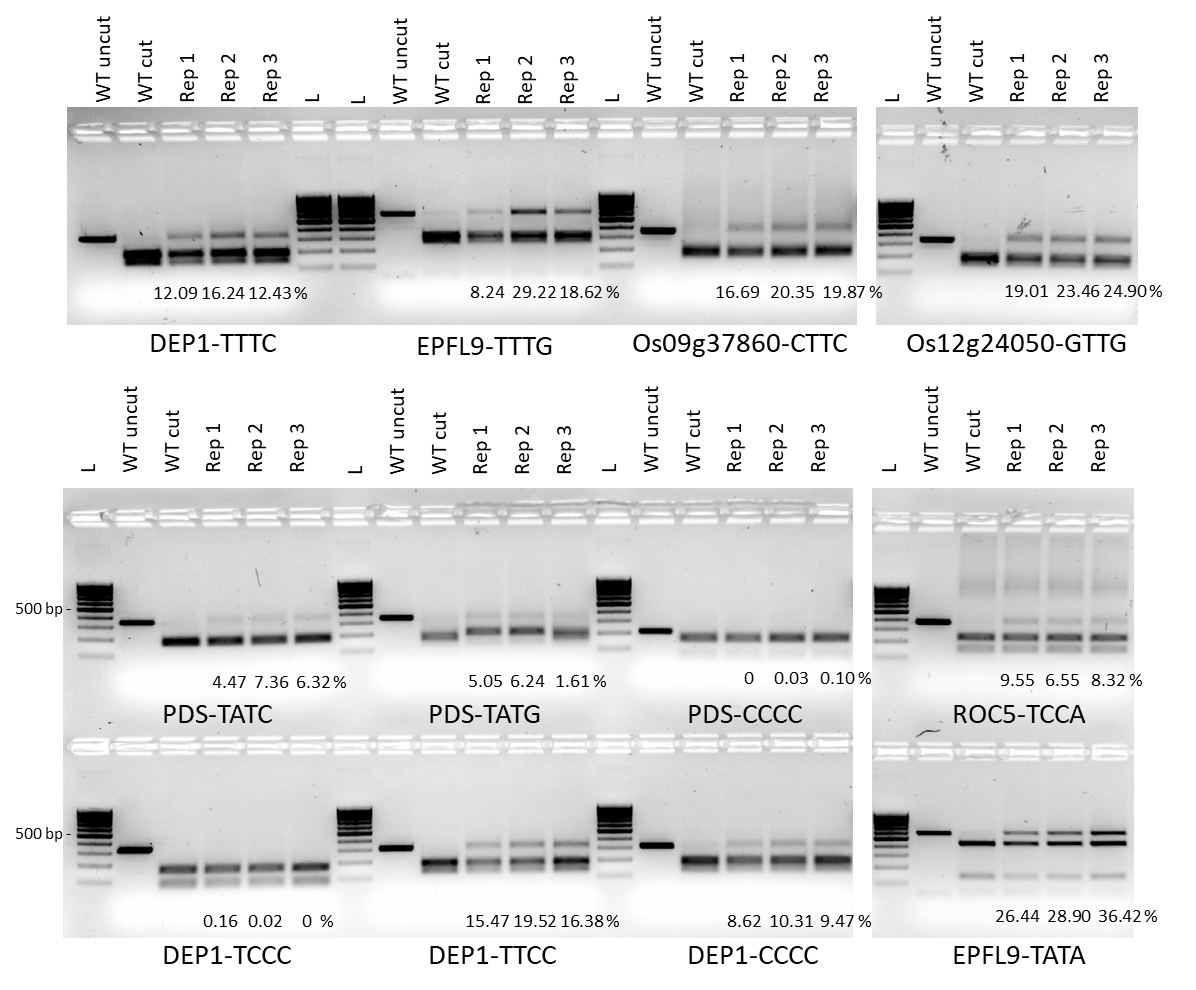


Panel 4 (Continued). Original gel images for Figure 2e. Agarose gels were stained using ethidium bromide and visualized using the Bio-Rad ChemiDoc Imaging System. L, NEB 100 bp DNA Ladder. WT, wild type rice protoplast. Uncut, PCR amplicon of target sites before restriction enzyme digestion. Cut, PCR amplicon of target sites after restriction enzyme digestion. Asterisks indicate non-specific amplifications. Editing efficiencies (%) shown under each lane were calculated by subtracting the editing efficiencies of WT. Each experiment includes three biological replicates.


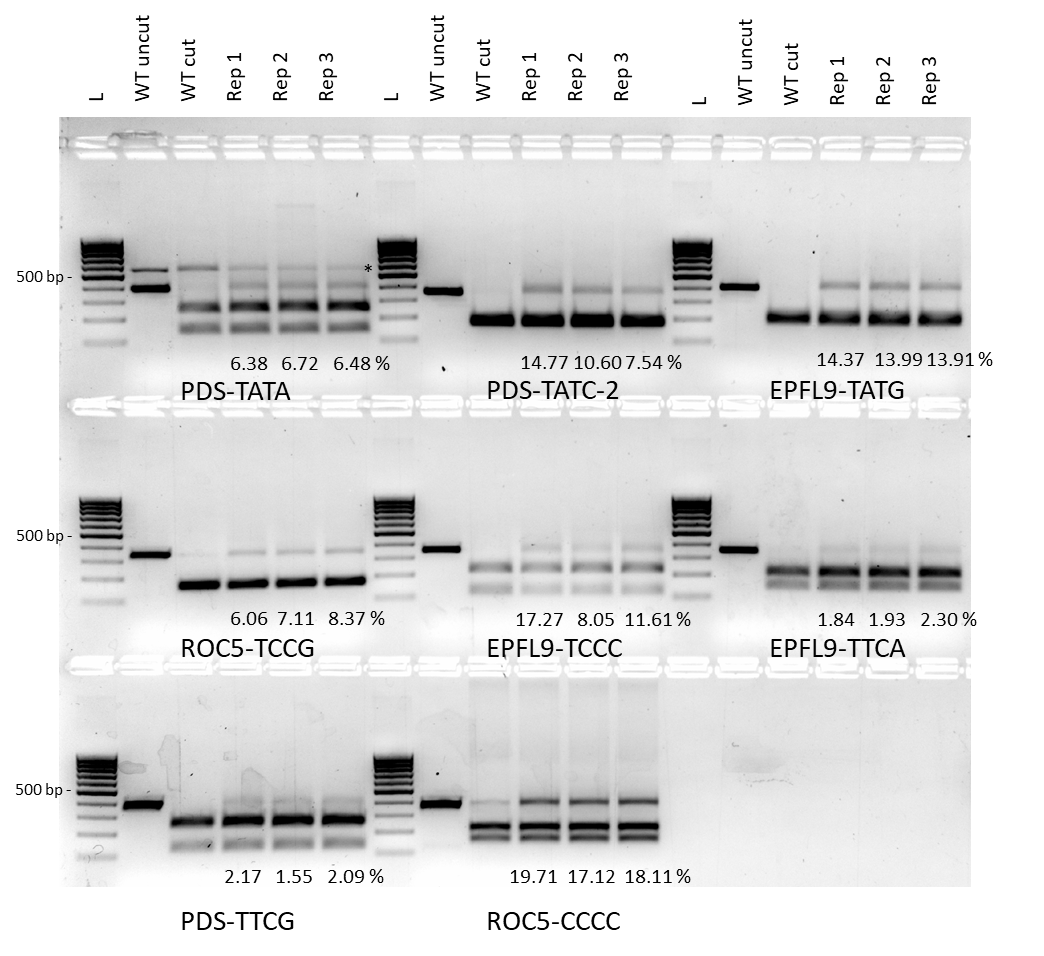


Panel 4 (Continued). Original gel images for Figure 2e. Agarose gels were stained using ethidium bromide and visualized using the Bio-Rad ChemiDoc Imaging System. L, NEB 100 bp DNA Ladder. WT, wild type rice protoplast. Uncut, PCR amplicon of target sites before restriction enzyme digestion. Cut, PCR amplicon of target sites after restriction enzyme digestion. Asterisks indicate non-specific amplifications. Editing efficiencies (%) shown under each lane were calculated by subtracting the editing efficiencies of WT. Each experiment includes three biological replicates.


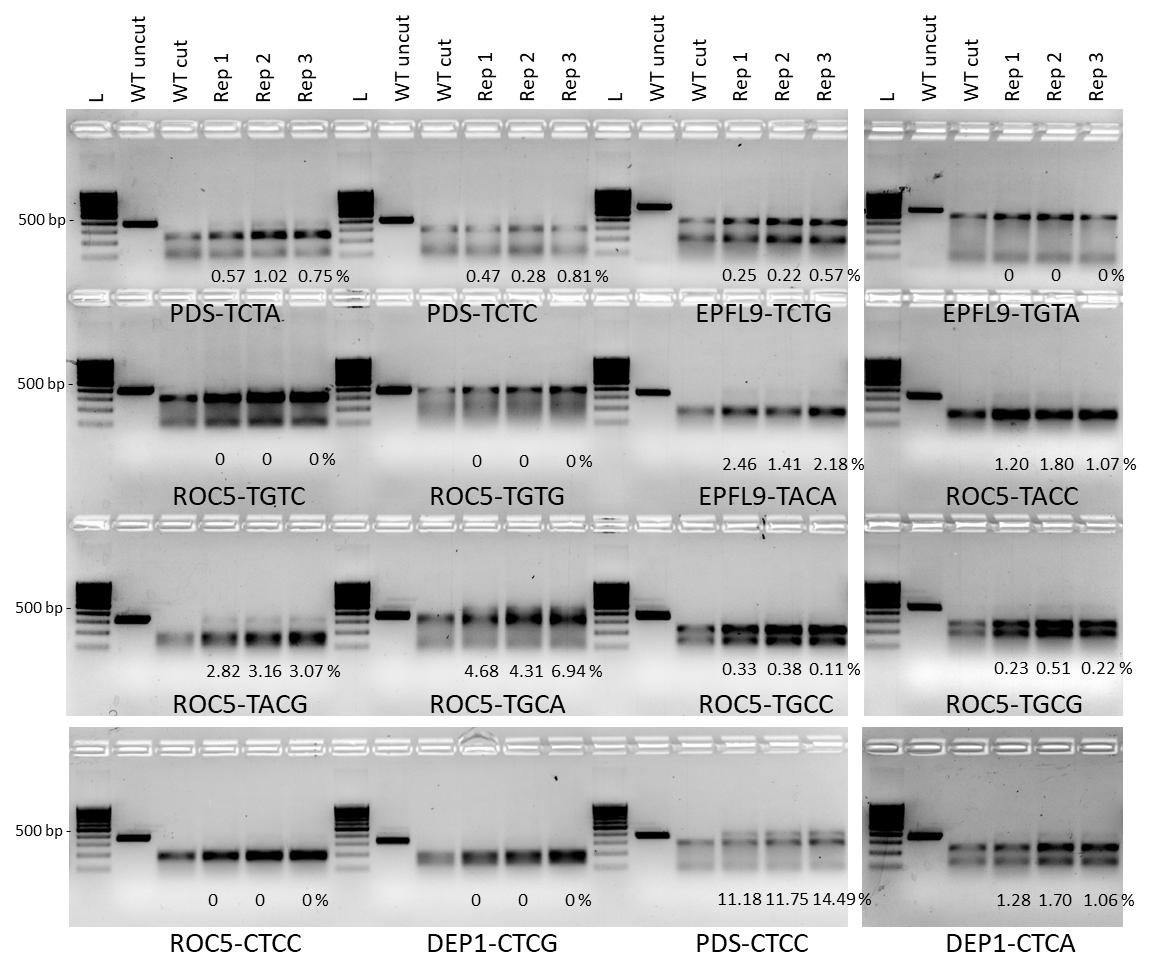


Panel 4 (Continued). Original gel images for Figure 2e. Agarose gels were stained using ethidium bromide and visualized using the Bio-Rad ChemiDoc Imaging System. L, NEB 100 bp DNA Ladder. WT, wild type rice protoplast. Uncut, PCR amplicon of target sites before restriction enzyme digestion. Cut, PCR amplicon of target sites after restriction enzyme digestion. Asterisks indicate non-specific amplifications. Editing efficiencies (%) shown under each lane were calculated by subtracting the editing efficiencies of WT. Each experiment includes three biological replicates.


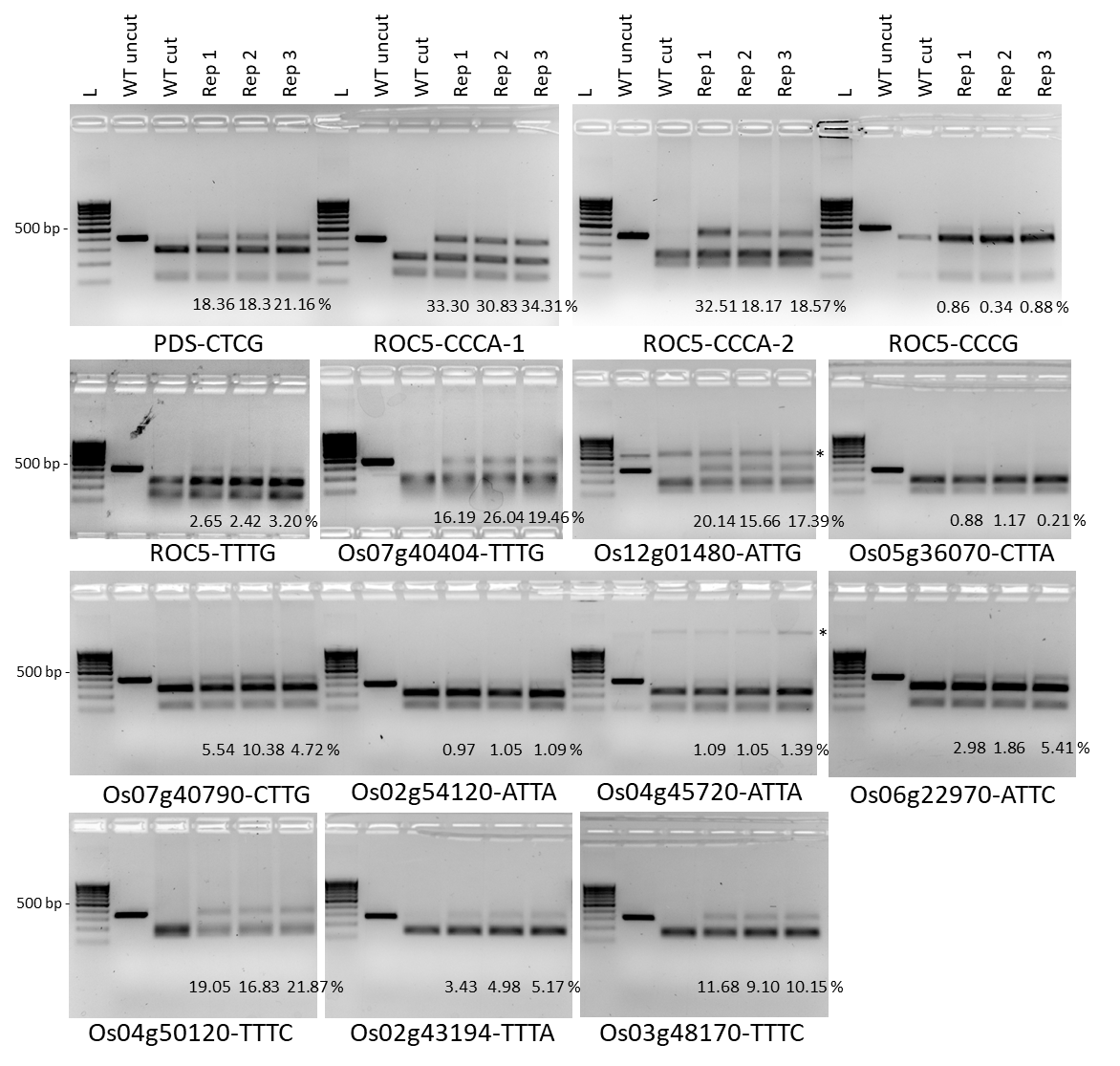


Panel 5. Original gel images for Figure 3d, e, f and Supplementary Figure 14. Agarose gels were stained using ethidium bromide and visualized using the Bio-Rad ChemiDoc Imaging System. L, NEB 100 bp DNA Ladder. WT, wild type rice plants. Uncut, PCR amplicon of target sites before restriction enzyme digestion. Cut, PCR amplicon of target sites after restriction enzyme digestion. N, non-transgenic plants.


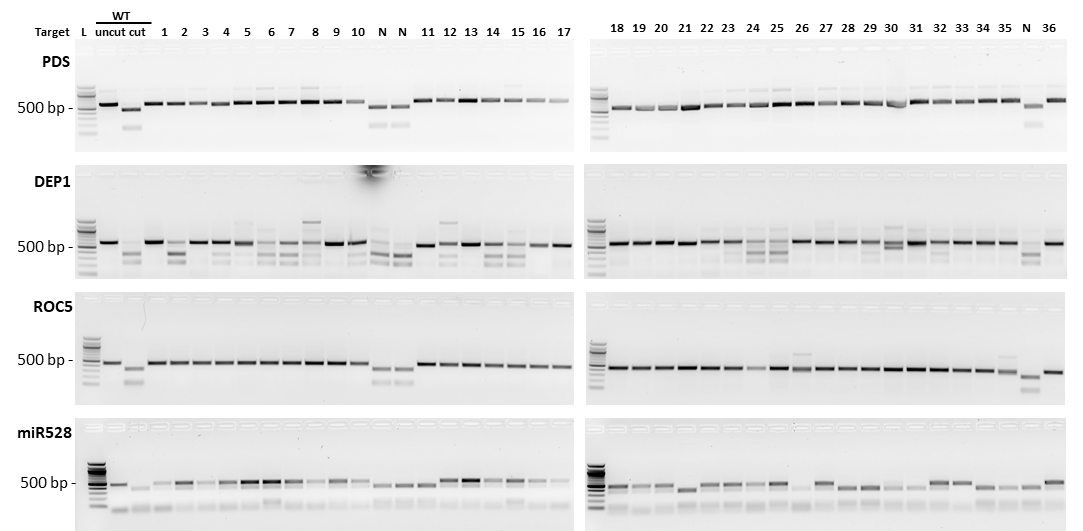


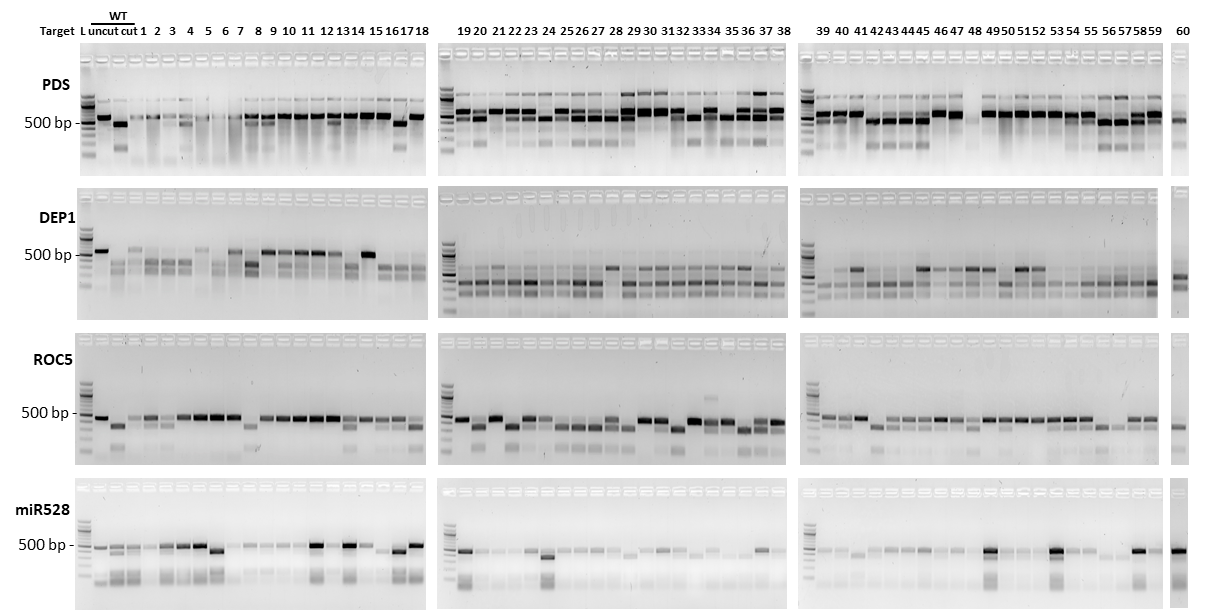
Panel 6. Original gel images for Figure 3d, e, f and Supplementary Figure 15. Agarose gels were stained using ethidium bromide and visualized using the Bio-Rad ChemiDoc Imaging System. L, NEB 100 bp DNA Ladder. WT, wild type rice plants. Uncut, PCR amplicon of target sites before restriction enzyme digestion. Cut, PCR amplicon of target sites after restriction enzyme digestion.


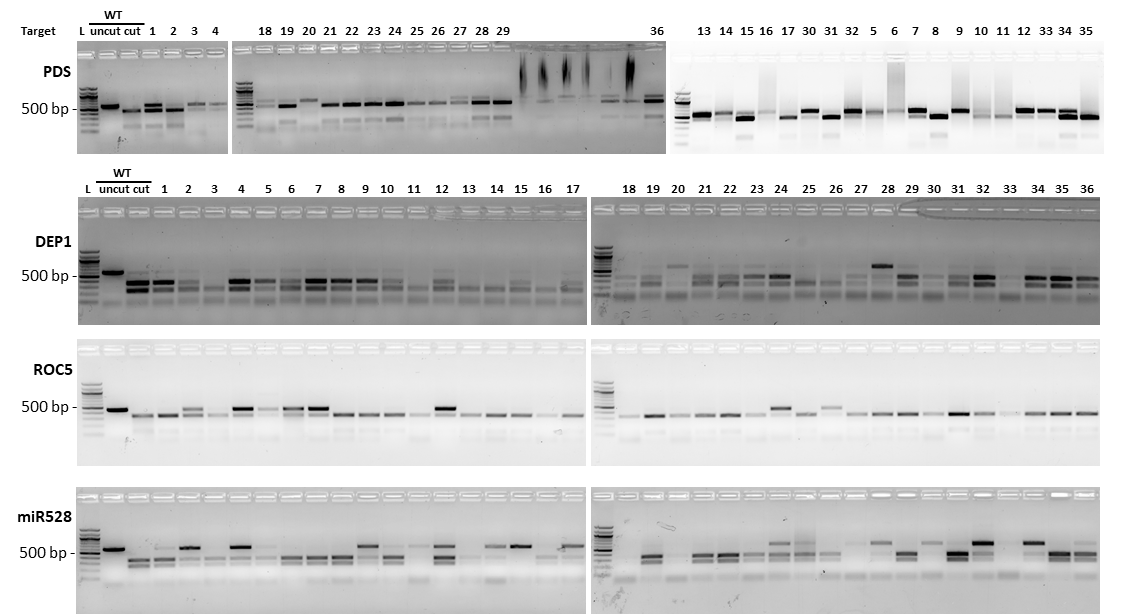
Panel 7. Original gel images for Figure 3d, e, f and Supplementary Figure 16. Agarose gels were stained using ethidium bromide and visualized using the Bio-Rad ChemiDoc Imaging System. L, NEB 100 bp DNA Ladder. WT, wild type rice plants. Uncut, PCR amplicon of target sites before restriction enzyme digestion. Cut, PCR amplicon of target sites after restriction enzyme digestion.

Panel 8. Original gel images for Figure 3d, e, f and Supplementary Figure 17. Agarose gels were stained using ethidium bromide and visualized using the Bio-Rad ChemiDoc Imaging System. L, NEB 100 bp DNA Ladder. WT, wild type rice plants. Uncut, PCR amplicon of target sites before restriction enzyme digestion. Cut, PCR amplicon of target sites after restriction enzyme digestion. The asterisk indicates non-specific amplifications.


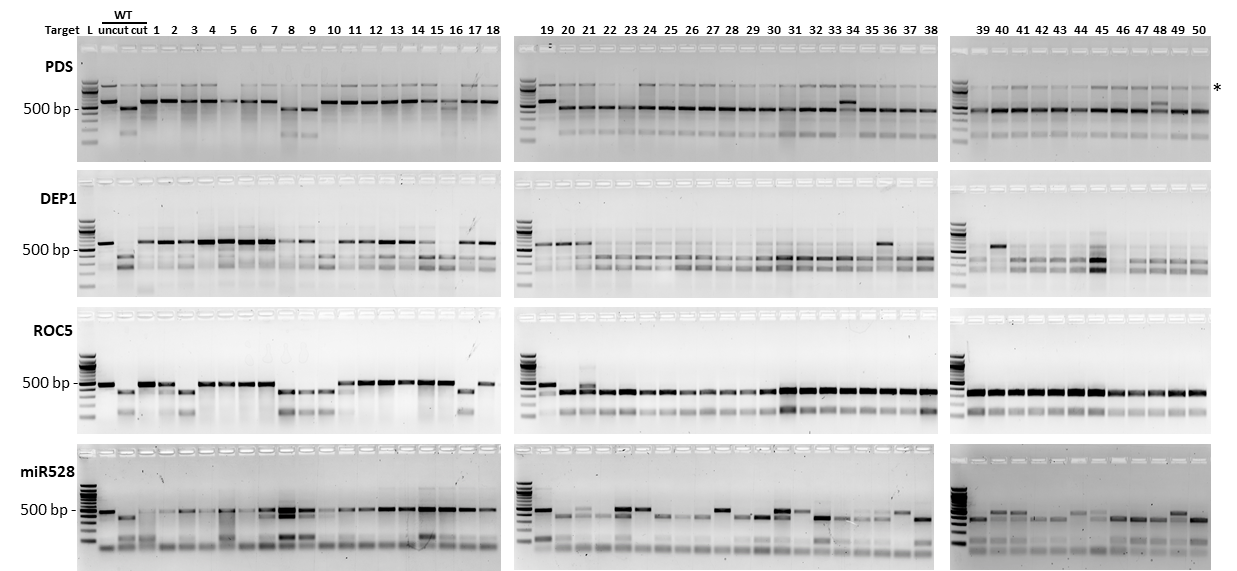


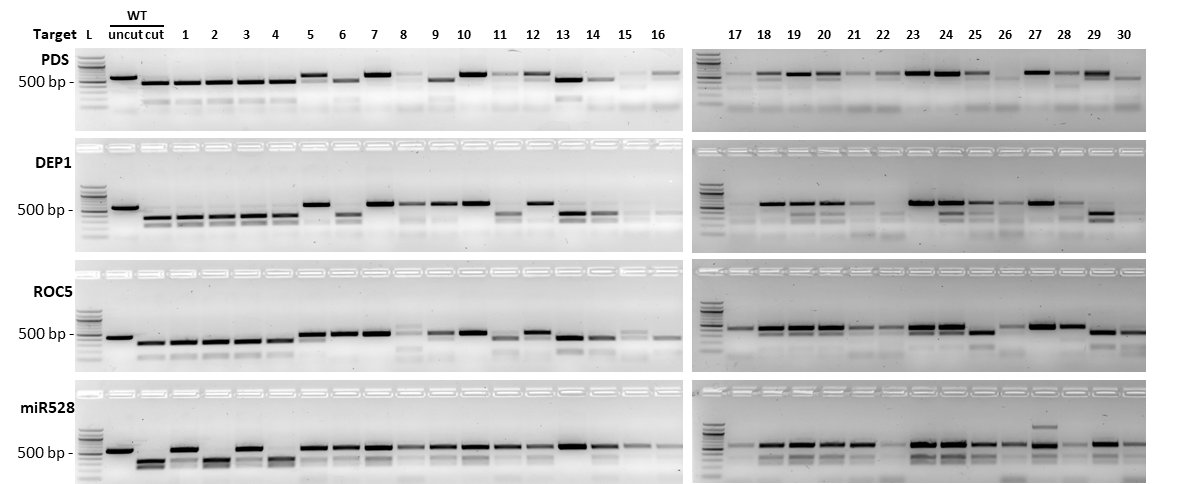
Panel 9. Original gel images for Figure 3d, e, f and Supplementary Figure 18. Agarose gels were stained using ethidium bromide and visualized using the Bio-Rad ChemiDoc Imaging System. L, NEB 100 bp DNA Ladder. WT, wild type rice plants. Uncut, PCR amplicon of target sites before restriction enzyme digestion. Cut, PCR amplicon of target sites after restriction enzyme digestion.


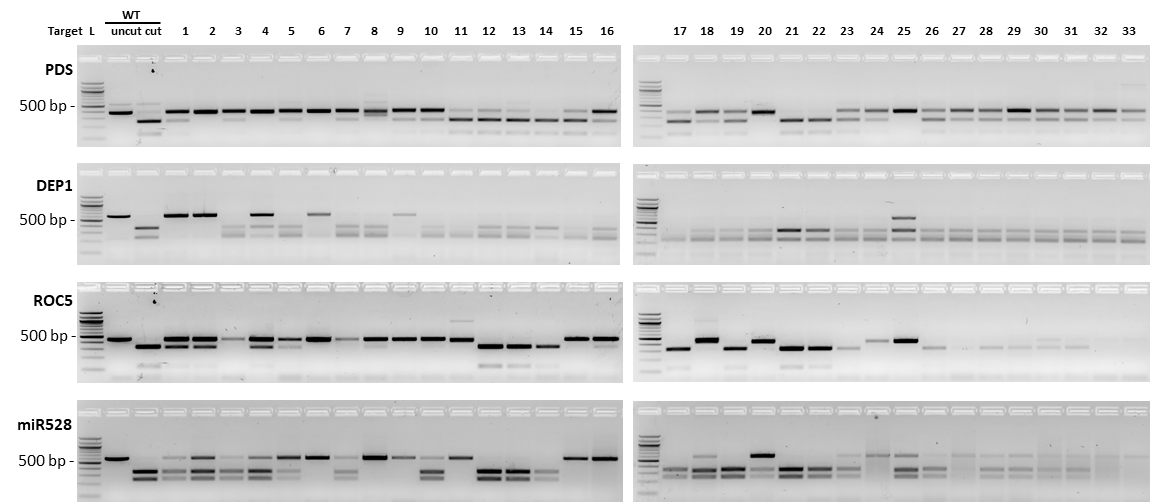
Panel 10. Original gel images for Figure 3d, e, f and Supplementary Figure 19. Agarose gels were stained using ethidium bromide and visualized using the Bio-Rad ChemiDoc Imaging System. L, NEB 100 bp DNA Ladder. WT, wild type rice plants. Uncut, PCR amplicon of target sites before restriction enzyme digestion. Cut, PCR amplicon of target sites after restriction enzyme digestion.

Panel 11. Original gel images for Figure 3d, e, f and Supplementary Figure 20. Agarose gels were stained using ethidium bromide and visualized using the Bio-Rad ChemiDoc Imaging System. L, NEB 100 bp DNA Ladder. WT, wild type rice plants. Uncut, PCR amplicon of target sites before restriction enzyme digestion. Cut, PCR amplicon of target sites after restriction enzyme digestion.


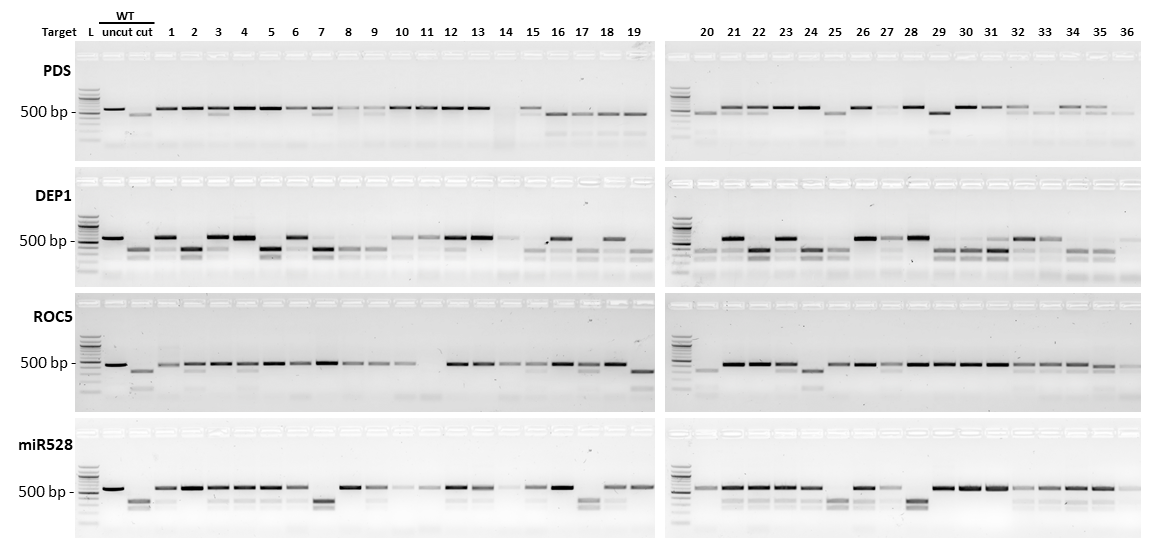


Panel 12. Original gel images for Supplementary Figure 23. Agarose gels were stained using ethidium bromide and visualized using the Bio-Rad ChemiDoc Imaging System. L, NEB 100 bp DNA Ladder. WT, wild type rice protoplast. Uncut, PCR amplicon of target sites before restriction enzyme digestion. Cut, PCR amplicon of target sites after restriction enzyme digestion. Asterisks indicate non-specific amplifications. Editing efficiencies (%) shown under each lane were calculated by subtracting the editing efficiencies of WT. Each experiment includes three biological replicates.


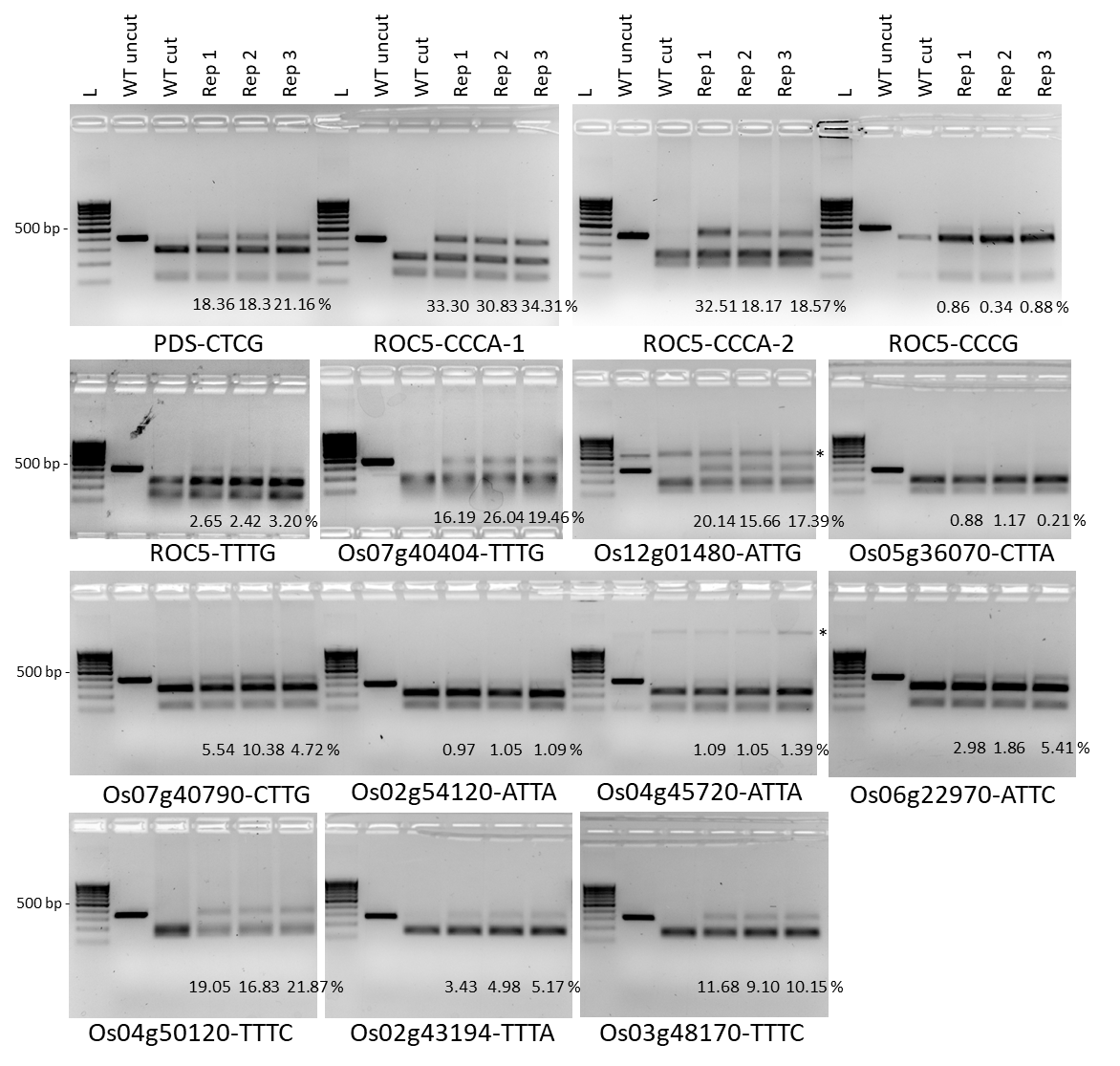


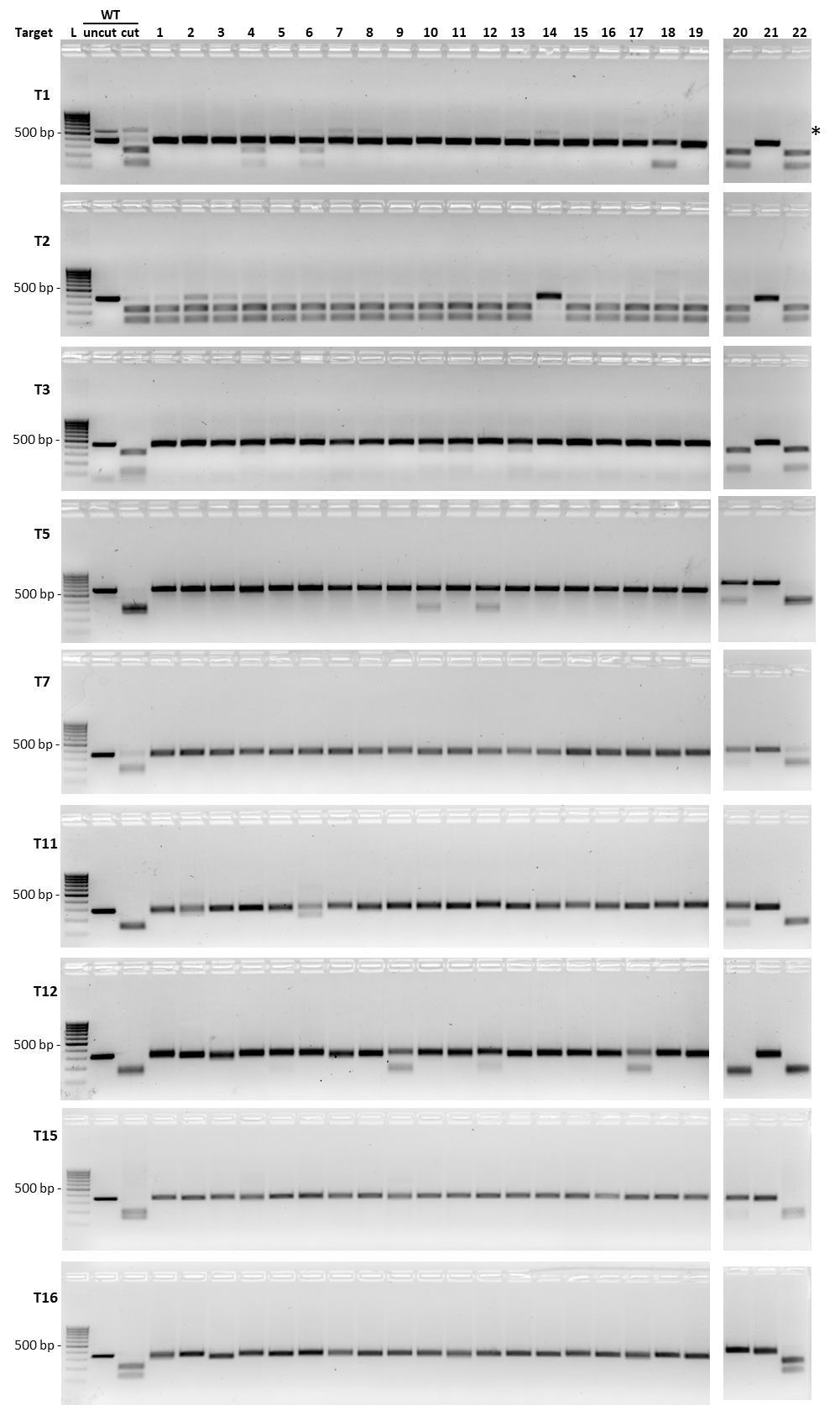
Panel 13. Original gel images for Supplementary Figure 26. Agarose gels were stained using ethidium bromide and visualized using the Bio-Rad ChemiDoc Imaging System. L, NEB 100 bp DNA Ladder. WT, wild type rice protoplast. Uncut, PCR amplicon of target sites before restriction enzyme digestion. Cut, PCR amplicon of target sites after restriction enzyme digestion. The asterisk indicates non-specific amplifications.
